# Reduced symmetric dimethylation stabilizes vimentin and promotes metastasis in MTAP‐deficient lung cancer

**DOI:** 10.15252/embr.202154265

**Published:** 2022-06-29

**Authors:** Wen‐Hsin Chang, Yi‐Ju Chen, Yi‐Jing Hsiao, Ching‐Cheng Chiang, Chia‐Yu Wang, Ya‐Ling Chang, Qi‐Sheng Hong, Chien‐Yu Lin, Shr‐Uen Lin, Gee‐Chen Chang, Hsuan‐Yu Chen, Yu‐Ju Chen, Ching‐Hsien Chen, Pan‐Chyr Yang, Sung‐Liang Yu

**Affiliations:** ^1^ Institute of Molecular Medicine College of Medicine, National Taiwan University Taipei Taiwan; ^2^ Institute of Chemistry Academia Sinica Taipei Taiwan; ^3^ Department of Clinical Laboratory Sciences and Medical Biotechnology College of Medicine, National Taiwan University Taipei Taiwan; ^4^ Institute of Statistical Science Academia Sinica Taipei Taiwan; ^5^ Graduate Institute of Oncology College of Medicine, National Taiwan University Taipei Taiwan; ^6^ Division of Chest Medicine, Department of Internal Medicine Taichung Veterans General Hospital Taichung Taiwan; ^7^ School of Medicine Chung Shan Medical University Taichung Taiwan; ^8^ Division of Pulmonary, Critical Care, and Sleep Medicine Department of Internal Medicine University of California Davis Davis CA USA; ^9^ Division of Nephrology, Department of Internal Medicine University of California Davis Davis CA USA; ^10^ Comprehensive Cancer Center University of California Davis Davis CA USA; ^11^ Department of Internal Medicine, College of Medicine National Taiwan University Taipei Taiwan; ^12^ Institute of Biomedical Sciences Academia Sinica Taipei Taiwan; ^13^ Institute of Medical Device and Imaging, College of Medicine National Taiwan University Taipei Taiwan; ^14^ Graduate Institute of Pathology, College of Medicine National Taiwan University Taipei Taiwan; ^15^ Graduate Institute of Clinical Medicine, College of Medicine National Taiwan University Taipei Taiwan; ^16^ Department of Laboratory Medicine National Taiwan University Hospital Taipei Taiwan

**Keywords:** Methylproteome, Methylthioadenosine (MTA), Post‐translational modification (PTM), Protein arginine methyltransferase 5 (PRMT5), Symmetric dimethylarginine (sDMA), Cancer, Cell Adhesion, Polarity & Cytoskeleton, Post-translational Modifications & Proteolysis

## Abstract

The aggressive nature and poor prognosis of lung cancer led us to explore the mechanisms driving disease progression. Utilizing our invasive cell‐based model, we identified methylthioadenosine phosphorylase (MTAP) and confirmed its suppressive effects on tumorigenesis and metastasis. Patients with low MTAP expression display worse overall and progression‐free survival. Mechanistically, accumulation of methylthioadenosine substrate in MTAP‐deficient cells reduce the level of protein arginine methyltransferase 5 (PRMT5)‐mediated symmetric dimethylarginine (sDMA) modification on proteins. We identify vimentin as a dimethyl‐protein whose dimethylation levels drop in response to MTAP deficiency. The sDMA modification on vimentin reduces its protein abundance but trivially affects its filamentous structure. In MTAP‐deficient cells, lower sDMA modification prevents ubiquitination‐mediated vimentin degradation, thereby stabilizing vimentin and contributing to cell invasion. MTAP and PRMT5 negatively correlate with vimentin in lung cancer samples. Taken together, we propose a mechanism for metastasis involving vimentin post‐translational regulation.

## Graphical abstract

Repression of MTAP‐dependent symmetric dimethylation mediated by PRMT5 increases vimentin protein stability and leads to invasion and metastasis in MTAP‐deficient lung cancer.

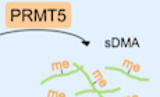

## Introduction

Highly aggressive in nature, lung cancer poses many challenges in both detection and management (Herbst *et al*, 2018; Siegel *et al*, 2020). Although a variety of treatments, including surgery, radiation, and chemotherapy, are well administered, ongoing complications involving cancer drug resistance and metastasis abate their initial efficacy; therefore, there is an urgent need to identify intrinsic mechanisms in order to develop more effective and viable therapies. We previously established a series of lung adenocarcinoma cell lines with various degrees of invasiveness as a platform to study mechanisms of metastatic process (Chu *et al*, 1997; Chen *et al*, 2001). Although we have thus far discovered dysregulation in multiple genes responsible for metastasis by conducting expression microarray assays (Chen *et al*, 2012, 2014, 2016; Chang *et al*, 2016; Hsu *et al*, 2018), many alterations are yet to be elucidated completely.

In the era of omics, genomic, transcriptomic and proteomic profiling have aided in delineating the molecular pathogenesis of cancer (Cancer Genome Atlas Research Network, 2014; Sanchez‐Vega *et al*, 2018). In conjunction with the detection of structural variations and phosphoproteome, those multi‐dimensional analyses further guide the development of genetic testing and therapeutic options (Sanchez‐Vega *et al*, 2018; Chen *et al*, 2020). However, signaling pathways are driven by post‐translational modifications (PTMs) on proteins under certain conditions. In addition to phosphorylation, other types of modification have recently gained attention given the crosstalk between different PTMs (Wu *et al*, 2019); therefore, a comprehensive screening for a specific PTM may shed new insight into novel therapeutic targets.

Methylthioadenosine phosphorylase (MTAP) is an enzyme responsible for catalyzing the phosphorylation of methylthioadenosine (MTA), a by‐product metabolite produced by the polyamine pathway, into methylthioribose‐1‐phosphate (MTR‐1‐P) and adenine for the salvage of methionine and adenine (Bertino *et al*, 2011). Clinically, approximately 15% of lung cancer patients carry MTAP gene deletion, and cancer cells with MTAP loss exhibit an increase of intracellular levels of MTA (Kryukov *et al*, 2016; Marjon *et al*, 2016; Mavrakis *et al*, 2016). Prior work reported that MTAP‐deficient cells are preferentially vulnerable to the depletion of protein arginine methyltransferase 5 (PRMT5), and MTA metabolite selectively inhibits the enzymatic activity of PRMT5 (Kryukov *et al*, 2016; Marjon *et al*, 2016; Mavrakis *et al*, 2016). PRMTs are classified into three types: all of the type I, II, and III PRMTs can catalyze the formation of monomethylarginine (mMA); the following generation of asymmetric dimethylarginine (aDMA) is catalyzed by type I PRMTs, and symmetric dimethylarginine (sDMA) is generated by type II PRMTs including PRMT5. The general biological functions of PRMTs include, but are not limited to, gene transcription, chromatin structure remodeling, RNA metabolism, and signal transduction, depending on the substrate proteins they methylate (Guccione & Richard, 2019). Although MTAP deletion sensitizes cancer cells to PRMT5 depletion, pharmacologic inhibition of PRMT5 by a specific small‐molecule inhibitor shows only modest selective tumor cytotoxicity (Kryukov *et al*, 2016; Marjon *et al*, 2016; Mavrakis *et al*, 2016). Thus, identification of viable molecular targets is of utmost importance for developing effective therapeutic options for MTAP‐loss cancer.

Herein, we investigated the role of MTAP in lung cancer metastasis, and elucidated its underlying mechanisms by using a methylproteomic screen. We further identified vimentin as a downstream metastatic executor in MTAP‐deficient cells. The aim of this study is to provide potential therapeutic targets for lung cancer.

## Results

### MTAP deficiency confers the tumorigenesis and metastatic ability of lung adenocarcinoma

**Figure 1:**
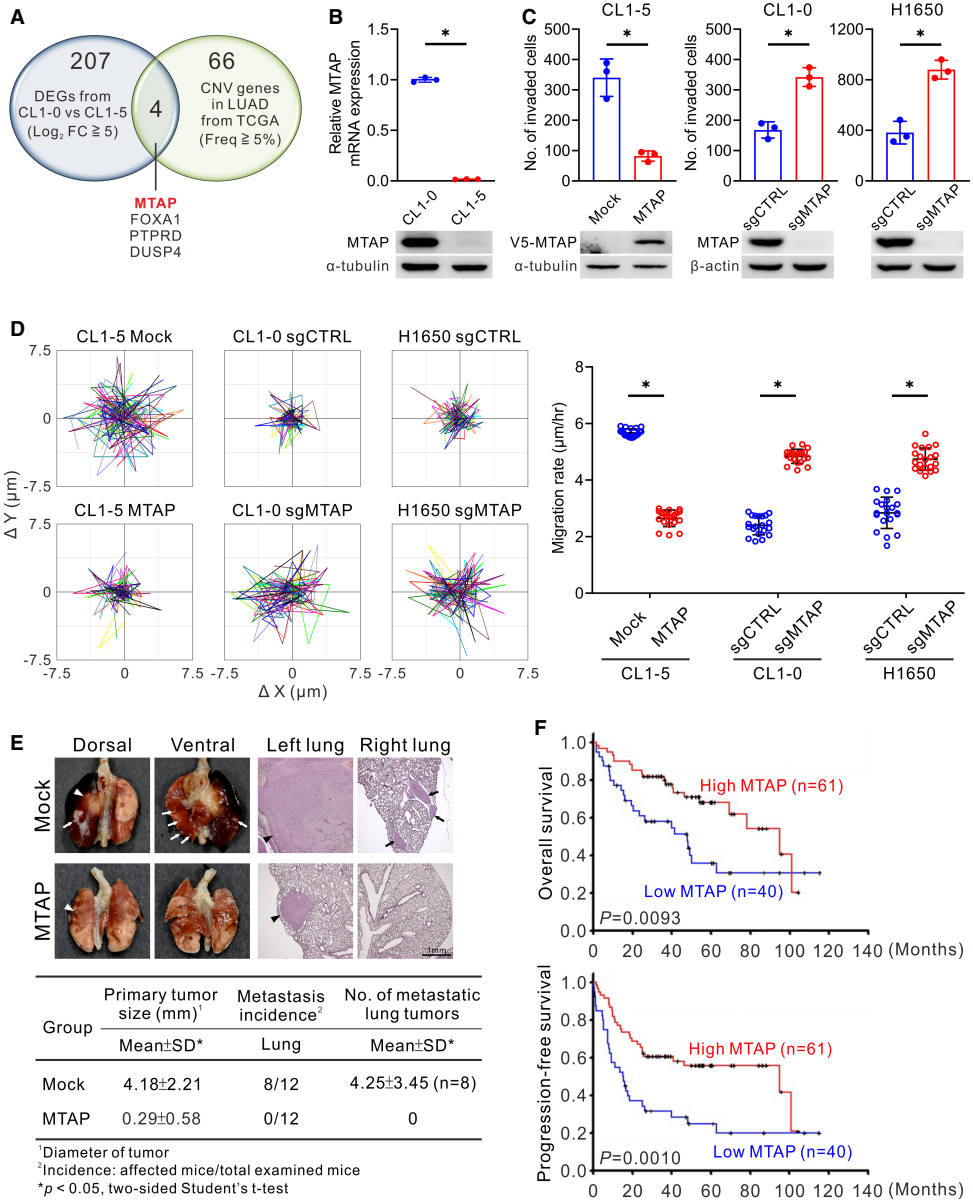
MTAP loss promotes invasion/metastasis of lung adenocarcinoma AThe Venn diagram showing 207 differentially expressed genes (DEGs) between CL1‐0 and CL1‐5 cells (fold change greater than 5‐fold in Log_2_ scale) and 66 genes with copy number variation (CNV, Frequency ≧5%) in lung adenocarcinoma (LUAD) from TCGA database (PanCancer Atlas) and the 4 intersected genes.BComparisons of *MTAP* mRNA and protein expression between CL1‐0 and CL1‐5 cells detected by RT‐qPCR (top, mean ± SD, Student *t* test, *n* = 3, biological replicates, *P < 0.05) and Western blot assays using anti‐MTAP antibody (bottom, representative of three independent experiments).CCell invasion abilities of MTAP‐overexpressing CL1‐5 or MTAP‐knockout CL1‐0 and H1650 were determined by Boyden chamber invasion assays (mean ± SD, Student *t* test, *n* = 3, biological replicates, *P < 0.05). sgCTRL indicates cells transduced with a nontargeting control sgRNA, and sgMTAP indicates cells transduced with a sgRNA targeting MTAP. Bottom: the protein expression levels of V5‐MTAP and endogenous MTAP were detected by Western blots.DMigration assays using a single‐cell tracking, time‐lapse video microscopy system. Representative trajectories and quantification of averaged velocity of cells (mean ± SD, Student *t* test, *n* = 20, technical replicates, *P < 0.05). Data shown are representative of three independent experiments.EEffect of MTAP overexpression on lung cancer metastasis *in vivo* was demonstrated by orthotopic implantation assays. Top: representative photographs of lungs and H&E staining of the lung sections. The primary tumors are indicated by arrowheads and the metastatic nodules are indicated by arrows. Bottom: quantification of averaged primary tumor sizes, metastatic incidence and nodule number.FKaplan–Meier analyses of overall survival (top) and progression‐free survival (bottom) for 101 patients with lung adenocarcinoma grouped into high‐ or low‐MTAP mRNA expression measured by RT‐qPCR. *P* values were obtained by log‐rank test.

**Figure EV1:**
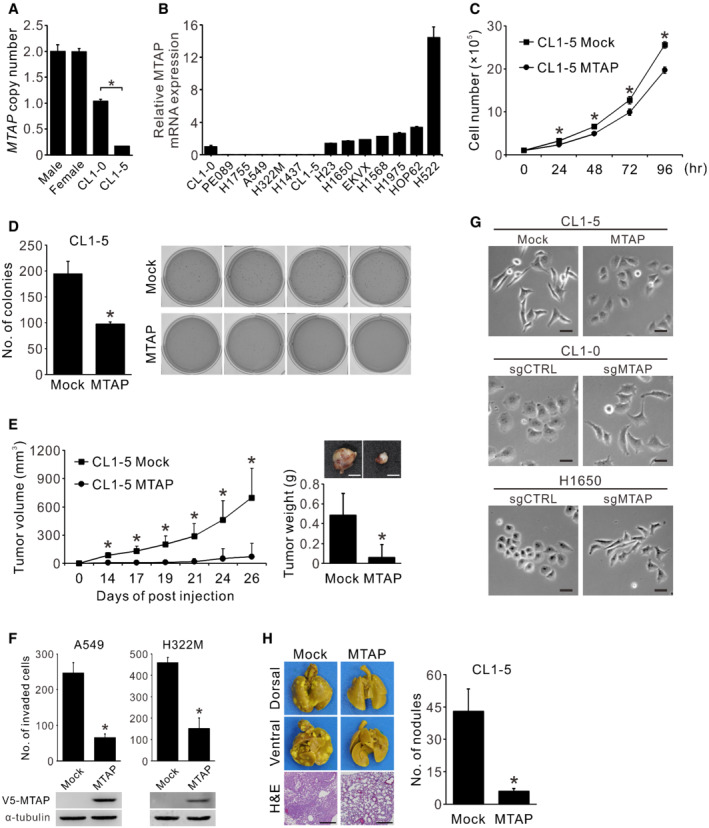
Expression and effect of MTAP on cell proliferation, colony forming, tumorigenesis, invasion, morphology, and metastasis in lung cancer cells AComparison of *MTAP* DNA copy number between PBMCs from healthy donors, CL1‐0, and CL1‐5 cells detected by real‐time genomic PCR (mean ± SD, Student *t* test, *n* = 3, biological replicates, *P < 0.05). Ribonuclease P (*RNaseP*) served as the internal control.BRelative mRNA expression of *MTAP* was detected in 14 lung cancer cell lines assayed by RT‐qPCR (mean ± SD, *n* = 3, biological replicates). TATA‐binding protein (*TBP*) served as the internal control.CEffect of MTAP on cell proliferation was determined by cell collection and counting at indicated time points (mean ± SD, Student *t* test, *n* = 3, technical replicates, *P < 0.05). Data shown are representative of three independent experiments.DEffect of MTAP on anchorage‐independent colony formation. Left: quantification of colonies stained by crystal violet and counted under phase microscopy (mean ± SD, Student *t* test, *n* = 4, biological replicates, *P < 0.05). Right: images of anchorage‐independent colony formation assays of CL1‐5 Mock control and MTAP‐overexpressing cells.ECL1‐5 Mock and MTAP‐overexpressing transfectants were subcutaneously injected into the NOD/SCID mice to determine the tumorigenesis ability. Left: the tumor volumes measured at indicated days (mean ± SD, Student *t* test, *n* = 8, biological replicates, *P < 0.05). Right: representative images of tumors and the tumor weights measured at 27 days postinjection (mean ± SD, Student *t* test, *n* = 8, biological replicates, *P < 0.05). Scale bar, 5 mm.FCell invasion abilities of MTAP‐overexpressing A549 and H322M were determined by Matrigel invasion assays (mean ± SD, Student *t* test, *n* = 3, biological replicates, *P < 0.05). Bottom: the protein expression levels of V5‐MTAP were detected by Western blots.GEffect of MTAP expression on cell morphology. Cells were examined by a phase contrast microscope. Scale bar, 50 μm. Data shown are representative of three independent experiments.HMicrometastatic analysis of MTAP‐overexpressing cells. NOD/SCID mice were intravenously injected with CL1‐5 Mock control and MTAP‐overexpressing cells, and sacrificed at 10 weeks post injection. Left: the appearances and the representative hematoxylin and eosin (H&E) staining of the lung sections from mice injected with CL1‐5 Mock and MTAP transfectants. Scale bar, 50 μm. Right: the gross pulmonary metastasis nodules were quantified under dissecting microscope (Mock, *n* = 11; MTAP, *n* = 9; biological replicates, mean ± SE, Student *t* test, *P < 0.05).

To unveil invasion/metastasis‐related gene expression alterations, we utilized our previous transcriptomic profiles from an isogenic invasion cell model (Chen *et al*, 2001) and copy number variation data from the Cancer Genome Atlas (TCGA) to identify potential gene candidates whose altered expression levels are associated with cancer cell invasion ability and copy number variations. Among the intersected genes, *MTAP* was downregulated (log_2_ ratio = −6.09) in highly invasive CL1‐5 compared to less invasive CL1‐0 cells, and its deletion frequency is 12.1% (62/511) in a TCGA cohort (Fig 1A). We confirmed decreased expression levels of *MTAP* mRNA and protein in CL1‐5 cells (Fig 1B), and found that compared to the PBMCs from healthy individuals, CL1‐0 displayed half level of copy number, whereas CL1‐5 had a severe copy number loss (Fig EV1A). In addition, *MTAP* mRNA expression was almost undetectable in 42.9% (6/14) of all tested lung cancer cell lines in our lab (Fig EV1B). Although a high percentage of *MTAP* deletion was found in several clinicopathologic cancer studies (Bertino *et al*, 2011; Su *et al*, 2014; Kryukov *et al*, 2016; Woollard *et al*, 2016), *MTAP* gene deletion associated with the invasive and/or metastatic processes is the first time to be mentioned.

Despite a previous report showing an inhibitory effect of MTAP on breast cancer cell growth (Christopher *et al*, 2002), the functional roles of MTAP in lung cancer remain unclear. We found that MTAP overexpression suppressed both cell proliferation and colonization capacity (Fig EV1C and D), and mice bearing subcutaneous MTAP‐overexpressing tumors exhibited smaller size and weight than those in mock control group (Fig EV1E). To prove that MTAP functions as a metastasis suppressor, we established both MTAP‐knockout and ‐overexpressing cells to examine their invasive and migratory abilities. As we expected, overexpression of MTAP inhibited both phenotypes, while MTAP knockout elevated invasion and migration abilities compared to control cells (Figs 1C and D, and EV1F). Moreover, the morphology of MTAP‐deficient cells tended to be mesenchymal‐like (Fig EV1G). We further carried out intravenous injection and orthotopic implantation assays to determine the role of MTAP in metastasis. An intravenous tail injection assay showed mice injected with MTAP‐overexpressing cells had less pulmonary metastasis nodules than those with mock control cells did (Fig EV1H). In an orthotopic xenograft model, smaller primary tumors and less metastatic tumor nodules were observed in mice receiving MTAP‐overexpressing cells (Fig 1E), indicating that MTAP inhibits the metastatic potential of lung cancer.

In light of an inhibitory effect of MTAP on tumorigenesis and metastasis, we next evaluated the clinical relevance of *MTAP* gene in patient outcome. MTAP expression in tumor specimens from 101 Taiwanese diagnosed with lung adenocarcinoma was detected by using RT‐qPCR assays. The clinical characteristics of these patients were summarized in Appendix Table S1. Patients with low MTAP expression showed worse overall survival and progression‐free survival as compared to those patients with high MTAP expression (Fig 1F and Appendix Table S2). Multivariate Cox regression analysis further confirmed that MTAP expression is an independent prognostic factor of overall survival (Appendix Table S3). These results suggest that MTAP functions as a tumor and metastasis suppressor.

### MTAP deletion inhibits PRMT5‐mediated sDMA of vimentin to promote cancer invasion

**Figure EV2:**
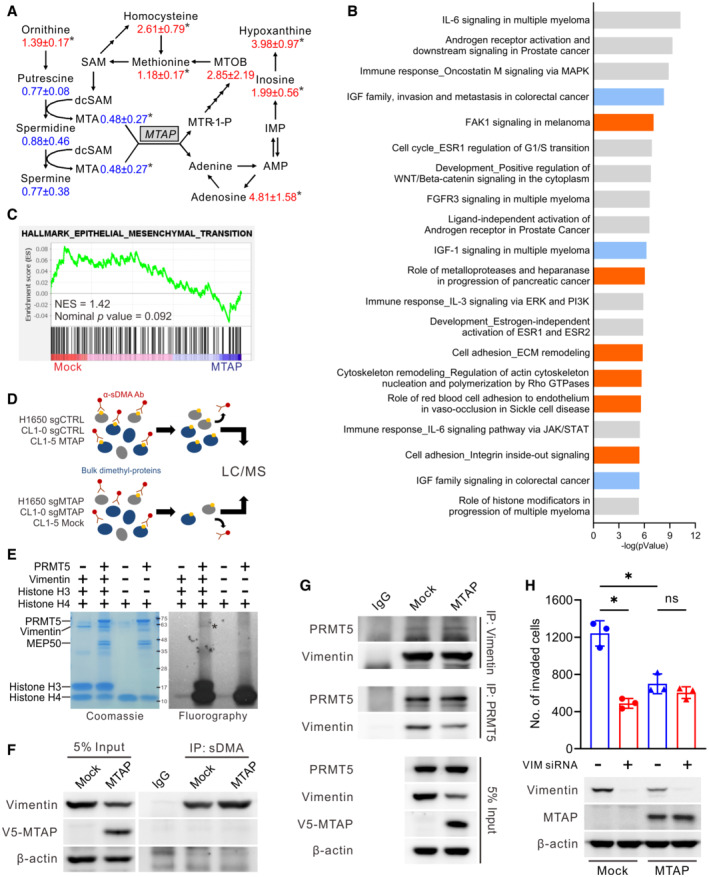
MTAP‐mediated metabolism, signaling pathways, and methylproteome AMTAP‐mediated metabolic alterations in polyamine, methionine, and adenine salvage pathways. The numbers are the average fold change intensities and associated errors for metabolites of CL1‐5 MTAP/CL1‐5 Mock (Student *t* test, *n* = 3, biological replicates, *P < 0.05).BTop 20 ranking MTAP‐altered pathways and cellular processes identified by the MetaCore analytical suite (version 20.4 build 70,300). Blue bars: IGF‐related pathways; Orange bars: cell adhesion/cytoskeleton remodeling and invasion‐related pathways.CGene Set Enrichment Analysis (GSEA) of expression microarray data from CL1‐5 Mock and MTAP‐overexpressing cells were performed using the gene set of HALLMARK_EPITHELIAL_MESENCHYMAL_TRANSITION.DSchematic diagram of the identification of differentially symmetrically dimethylated proteins.E
*In vitro* methylation of vimentin by PRMT5. Tritiated proteins were separated by SDS‐PAGE, stained with Coomassie blue (left), dried and analyzed by fluorography (right). Histone H3 and H4 proteins were used as positive controls. *: tritiated vimentin.FImmunoprecipitation analysis for vimentin dimethylation in CL1‐5 Mock and MTAP‐overexpressing cells. Data shown are representative of three independent experiments.GImmunoprecipitation analysis of the association between endogenous vimentin and endogenous PRMT5 in CL1‐5 Mock and MTAP‐overexpressing cells. Data shown are representative of three independent experiments.HCL1‐5 MTAP‐overexpressing and Mock cells were transfected with vimentin siRNAs for 72 h and analyzed by Boyden chamber invasion assays (mean ± SD, Student *t* test, *n* = 3, biological replicates, *P < 0.05). The silence efficiency of vimentin was examined by Western blot. Source data are available online for this figure.

We next performed targeted metabolomics analysis to verify the catalytic activity of ectopically expressed MTAP in lung cancer cells. The metabolic profiling of MTAP‐overexpressing and mock control CL1‐5 cells showed that MTA level was diminished by abundant MTAP, concomitant with an increase of downstream metabolites in methionine and adenine salvage cycles (Fig EV2A) and variations in metabolites involved in other pathways (Appendix Table S4), implying that ectopic expression of MTAP is functional. To investigate the molecular mechanism of MTAP‐mediated suppression of lung cancer metastasis, 1,042 differentially expressed genes with 2‐fold change from the mRNA profiling of mock and MTAP‐overexpressing CL1‐5 cells were subjected to pathway analysis. In addition to IGF‐related pathways as we described previously (Xu *et al*, 2019), cell adhesion/cytoskeleton remodeling and invasion‐related pathways were significantly affected by MTAP (Fig EV2B), but this transcriptomic data were not enriched in the epithelial–mesenchymal transition (EMT) signature (Fig EV2C).

**Figure 2:**
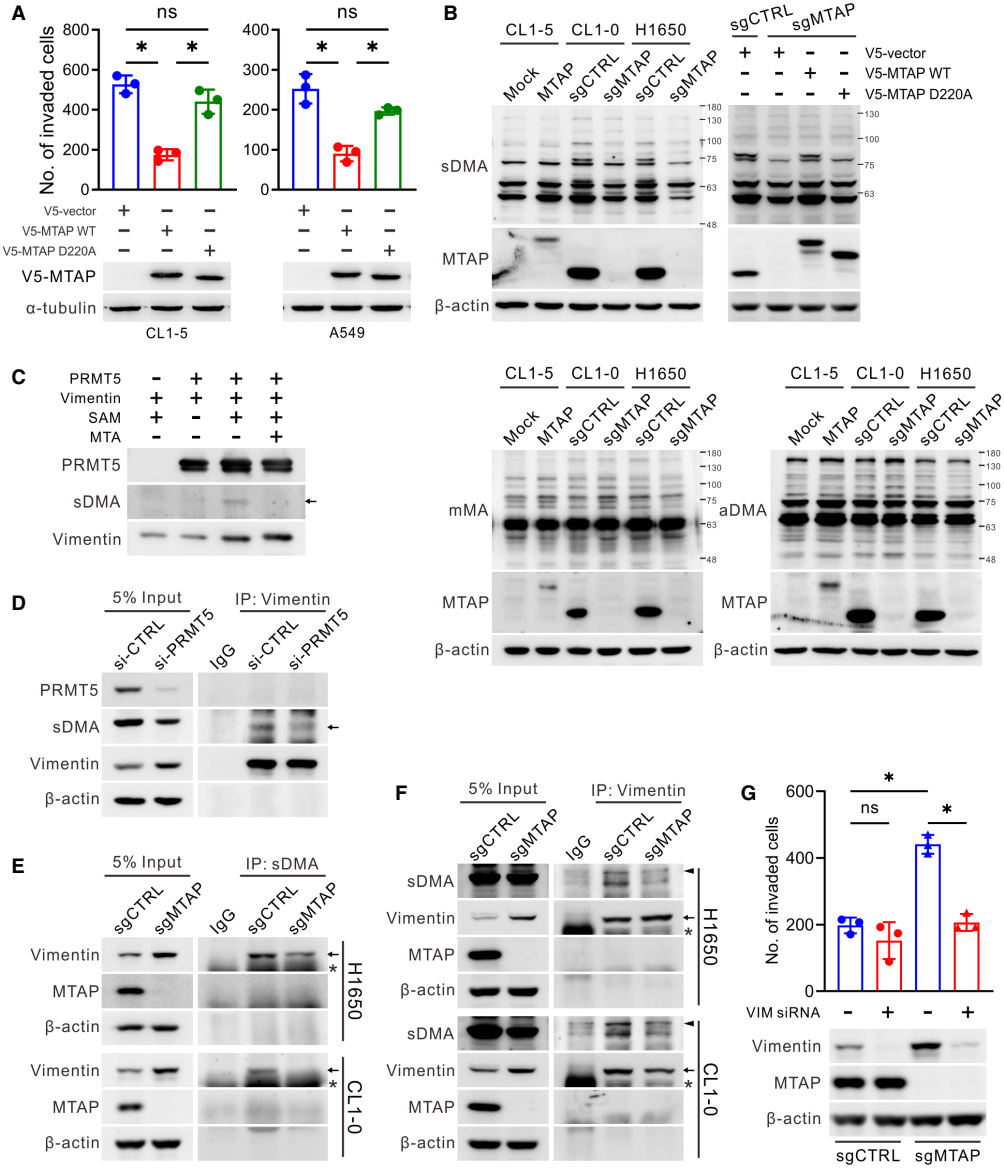
Vimentin is less dimethylated and involved in cancer invasion induced by MTAP deletion AThe necessity of MTAP activity in cell invasion. CL1‐5 and A549 cells were introduced with indicated plasmids and subjected to Boyden chamber invasion assays (mean ± SD, Student *t* test, *n* = 3, biological replicates, *P < 0.05 compared to the vector control group). The protein expression levels of exogenous wild‐type and mutant V5‐MTAP were detected by Western blots.BWestern blot analysis of the symmetric dimethylarginine (sDMA), monomethylarginine (mMA) and asymmetric dimethylarginine (aDMA) levels in MTAP‐overexpressing and MTAP‐knockout cells. V5‐MTAP wild‐type and D220A mutant were restored in H1650 MTAP‐knockout cells. Data shown are representative of three independent experiments.C
*In vitro* methyltransferase assays of vimentin by PRMT5 analyzed by Western blots with anti‐sDMA antibody. Arrow marks dimethyl‐vimentin.DImmunoprecipitation analysis for vimentin dimethylation in response to PRMT5 knockdown in H1650 cells. Arrow marks dimethyl‐vimentin. Data shown are representative of three independent experiments.E, FImmunoprecipitation analysis for vimentin dimethylation in control and MTAP‐knockout cells. Arrows mark vimentin, asterisks mark heavy chains, and arrowheads mark nonspecific bands. Data shown are representative of three independent experiments.GVimentin‐knockdown counteracts MTAP‐loss‐induced effect on cell invasion. MTAP‐knockout and control H1650 cells were transfected with vimentin‐specific siRNAs for 72 h and analyzed by Boyden chamber invasion assays (mean ± SD, Student *t* test, *n* = 3, biological replicates, *P < 0.05). The silence efficiency of vimentin was examined by Western blots.

We noticed that the invasion‐suppressive ability of enzyme‐defective D220A mutant MTAP which fails to catalyze MTA (Appleby *et al*, 1999; Xu *et al*, 2019) was attenuated (Fig 2A), suggesting that the enzymatic activity of MTAP is critical for its cancer suppressive function. Given the altered enzymatic activity of PRMTs in response to *MTAP* gene deletion and in view of MTA accumulation in MTAP‐deficient cells (Kryukov *et al*, 2016; Marjon *et al*, 2016; Mavrakis *et al*, 2016), the steady‐state levels of the three types of arginine methylation in *MTAP* gene‐modified cells were measured by Western blots. Among them, sDMA level, catalyzed by type II PRMTs including PRMT5, was significantly diminished in MTAP‐knockout CL1‐0 and H1650 cells due to MTA‐mediated inhibition of PRMT5 activity. Conversely, elevation of sDMA level was seen in CL1‐5 cells with ectopic expression of V5‐tagged MTAP (Fig 2B, top left), whereas no changes on the levels of mMA and aDMA occurred in the context of MTAP expression (Fig 2B, bottom). Overexpression of wild‐type MTAP but not D220A mutant MTAP almost rescued MTAP loss‐induced sDMA downregulation (Fig 2B, top right). Accordingly, we hypothesized that the tumor‐suppressive activity of MTAP is mediated through the MTA/PRMT5/protein dimethylation axis, and thus we identified those differentially symmetrically dimethylated proteins in response to MTAP alteration by mass spectrometric analyses.

Bulk dimethyl‐proteins were immunoprecipitated from cells of CL1‐5 Mock/MTAP, CL1‐0 sgCTRL/sgMTAP, and H1650 sgCTRL/sgMTAP by using anti‐sDMA antibody and then subjected to LC–MS/MS, and proteins with 2‐fold altered abundance were identified (Fig EV2D). Ninety‐nine proteins were identified in CL1‐5 MTAP/Mock, 213 proteins in CL1‐0 sgCTRL/sgMTAP, and 64 proteins in H1650 sgCTRL/sgMTAP. Notably, 24 proteins were commonly found in all three cell pairs, and 6 out of 24 had detectable arginine methylation site(s) in which vimentin showed the greatest changes in dimethyl‐protein abundance (Table 1 and Appendix Table S5). Although vimentin is a well‐known mesenchymal marker and functions in metastasis (Strouhalova *et al*, 2020), our understanding of this protein methylation is still limited. To determine whether vimentin is a substrate of PRMT5, we performed an *in vitro* methyltransferase assays in which the recombinant PRMT5 and vimentin were incubated with methyl donor S‐adenosylmethionine (SAM). We found that vimentin was directly methylated by PRMT5 and the sDMA level of vimentin was mostly abolished by MTA, which is an analog of SAM and competitive inhibitor of methyltransferases (Kryukov *et al*, 2016; Figs 2C and EV2E).

**Table 1:** Identification of differentially symmetrically dimethylated proteins.

Accession	Gene	Description	MW [kDa]	Fold in CL1‐5^a^	Fold in CL1‐0^b^	Fold in H1650^b^	Arginine Methylation site
P08670	VIM	Vimentin	53.6	5.4E+00	9.1E+05	1.7E+06	Methyl [R64; R69; R196; R321; R345; R378]
Q92804	TAF15	TATA‐binding protein‐associated factor 2N	61.8	3.0E+00	1.2E+07	6.0E+00	Methyl [R562; R570]
Q7Z406	MYH14	Myosin‐14	227.7	2.7E+01	2.4E+00	1.6E+07	Methyl [R189; R1912]
P14678	SNRPB	Small nuclear ribonucleoprotein‐associated proteins B and B′	24.6	1.7E+01	2.6E+00	6.3E+06	Methyl [R108]
P60709	ACTB	Actin, cytoplasmic 1	41.7	6.2E+00	1.7E+01	2.4E+01	Methyl [R62; R256; R372]
P35579	MYH9	Myosin‐9	226.4	1.6E+01	2.1E+00	6.9E+00	Methyl [R104; R165; R341; R358; R775; R842; R867; R930; R1051; R1107; R1191; R1912]
P06733	ENO1	Alpha‐enolase	47.1	2.6E+07	1.4E+07	2.6E+07	
Q96P63	SERPINB12	Serpin B12	46.2	5.1E+06	8.8E+06	3.3E+06	
P11182	DBT	Lipoamide acyltransferase component of branched‐chain alpha‐keto acid dehydrogenase complex, mitochondrial	53.5	9.5E+05	3.2E+07	8.0E+05	
P38159	RBMX	RNA‐binding motif protein, X chromosome	42.3	2.4E+05	9.2E+06	3.3E+06	
P08621	SNRNP70	U1 small nuclear ribonucleoprotein 70 kDa	51.5	4.8E+05	1.5E+07	2.7E+00	
P12956	XRCC6	X‐ray repair cross‐complementing protein 6	69.8	1.2E+07	2.5E+00	4.7E+06	
P25311	AZGP1	Zinc‐alpha‐2‐glycoprotein	34.2	1.6E+06	3.1E+00	3.2E+06	
Q96DI7	SNRNP40	U5 small nuclear ribonucleoprotein 40 kDa protein	39.3	5.3E+00	1.4E+06	7.0E+07	
P81605	DCD	Dermcidin	11.3	2.8E+07	3.4E+00	2.3E+00	
P04406	GAPDH	Glyceraldehyde‐3‐phosphate dehydrogenase	36.0	6.0E+06	5.5E+00	2.4E+00	
P19338	NCL	Nucleolin	76.6	6.3E+00	8.5E+06	3.5E+00	
O43172	PRPF4	U4/U6 small nuclear ribonucleoprotein Prp4	58.4	2.2E+00	8.2E+07	2.1E+00	
Q00839	HNRNPU	Heterogeneous nuclear ribonucleoprotein U	90.5	5.4E+00	2.5E+00	2.2E+00	
P09012	SNRPA	U1 small nuclear ribonucleoprotein A	31.3	3.6E+00	2.4E+00	6.1E+00	
P07355	ANXA2	Annexin A2	38.6	2.7E+00	9.0E+00	6.2E+00	
P61626	LYZ	Lysozyme C	16.5	2.6E+00	3.4E+00	2.2E+00	
P42704	LRPPRC	Leucine‐rich PPR motif‐containing protein, mitochondrial	157.8	2.2E+00	5.0E+00	2.3E+00	
P23246	SFPQ	Splicing factor, proline‐ and glutamine‐rich	76.1	2.1E+00	6.6E+01	2.1E+00	

^a^ MTAP/Mock.

^b^ sgCTRL/sgMTAP.

To understand whether vimentin is methylated by PRMT5 in cells, vimentin was immunoprecipitated from PRMT5‐knockdown cell lysates. Western blots demonstrated that the dimethylation level of vimentin was correspondingly decreased (Fig 2D). To further determine the contribution of MTAP to vimentin dimethylation, bulk dimethyl‐proteins from MTAP‐manipulated cells were immunoprecipitated by using anti‐sDMA antibody and then blotted by using anti‐vimentin antibody. Consistent with the results from mass spectrometric analysis, dimethyl‐vimentin was detected in MTAP‐overexpressing CL1‐5 cells and control cells of H1650 and CL1‐0, whereas this dimethyl abundance was decreased in CL1‐5 mock control cells and MTAP‐knockout cells despite that their inputs showed higher level of vimentin protein (Figs 2E and EV2F). In reverse immunoprecipitation assays using anti‐vimentin antibody, less dimethylated level was found in vimentin precipitated from MTAP‐knockout cells compared to that from control cells (Fig 2F). Collectively, the above observations suggest that vimentin is indeed a symmetrically dimethylated protein, and its sDMA level is positively regulated by MTAP. However, as shown in Fig EV2G, the differential methylation level of vimentin was not associated with its affinity with PRMT5. To determine whether the elevated invasiveness of MTAP‐loss cells is attributed to the less level of dimethylated vimentin, cells were transfected with vimentin‐specific siRNAs and then subjected to invasion assays. Consistently, MTAP‐deficient cells displayed higher invasion ability than MTAP‐expressing cells did. However, knockdown of vimentin greatly attenuated MTAP deficiency‐mediated invasion in both MTAP‐knockout and MTAP‐overexpressing cell models (Figs 2G and EV2H). Therefore, these results suggest that MTAP‐mediated sDMA on vimentin contributes to invasion inhibition in lung cancer.

### Decreased sDMA levels on vimentin enhances cancer cell motility

**Figure 3:**
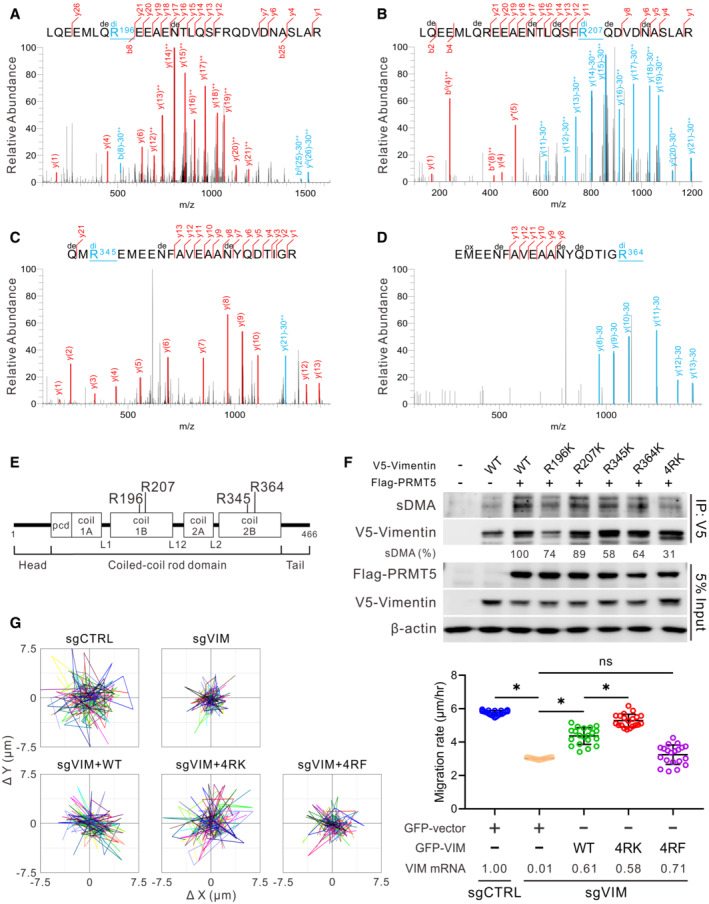
Suppression of vimentin sDMA level promotes cancer cell motility A–DIdentification of symmetrically dimethylated arginine residues on vimentin by mass spectrometry analysis. MS/MS spectra evidence of sDMA at R196 (A), R207 (B), R345 (C), and R364 (D) of vimentin protein in CL1‐5 cells. The sDMA‐modified fragments with neutral loss 30.034 at the residue arginine (R) were annotated by blue color. Di, dimethyl at R; de, deamidated at N or Q; ox, oxidation at M; ++, fragment with double charge.EIllustration of vimentin protein domains and four dimethylated arginine residues are highlighted.FValidation of the PRMT5‐catalyzed methylation sites on V5‐vimentin. HEK293T cells were transfected with indicated constructs and subjected to immunoprecipitation analysis. The intensity of sDMA signals was quantified by ImageJ software, normalized to the intensity of corresponding immunoprecipitated V5‐vimentin, then relative to the V5‐vimentin wild‐type group and represented as percentage. Data were the averaged percentage from three independent experiments.GSingle‐cell tracking migration assays. H1650 control (sgCTRL) and vimentin‐knockout (sgVIM) cells were transfected with GFP vector or GFP‐vimentin wild‐type, 4RK or 4RF mutant for 48 h. Cells expressing GFP fluorescence were tracked by the time‐lapse video microscopy system. Representative trajectories (left) and quantification of averaged velocity of cells (right, mean ± SD, Student *t* test, *n* = 20, technical replicates, *P < 0.05). 4RK indicates R196/207/345/364K; 4RF indicates R196/207/345/364F. Data shown are representative of three independent experiments.

**Figure EV3:**
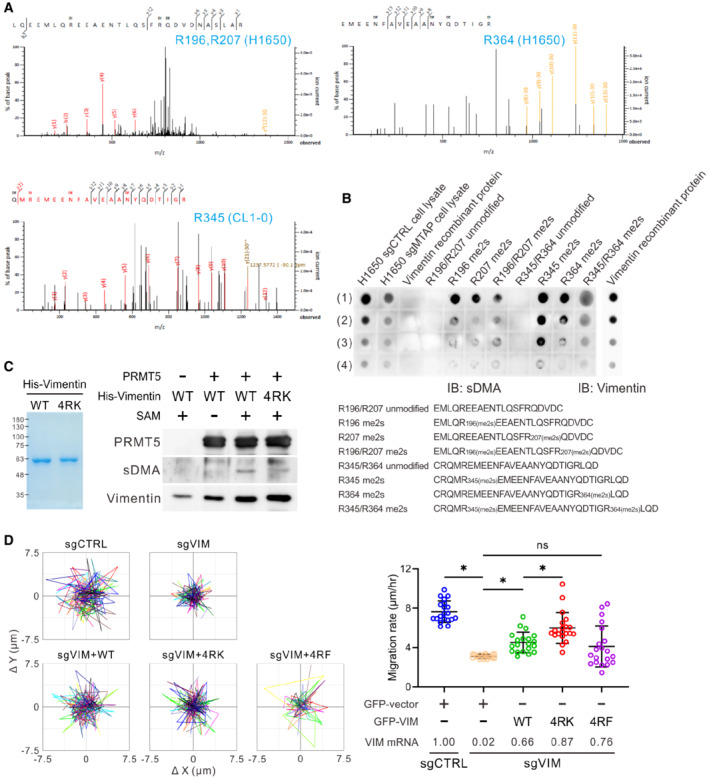
Effect of PRMT5‐mediated sDMA on vimentin‐governed cell motility AMS/MS spectra evidence of sDMA at R196, R207, and R364 from H1650 vimentin and R345 from CL1‐0 vimentin.BDot blot analysis showing specificity of sDMA antibody. H1650 cell lysates were used as positive controls. The sequences of non‐methylated (unmodified) and dimethylated (me2s) vimentin peptides were shown in the bottom. H1650 cell lysates: (1) 6.5 μg, (2) 3.2 μg, (3) 1.6 μg, (4) 0.8 μg. Vimentin recombinant protein: (1) 0.5 μg, (2) 0.25 μg, (3) 0.125 μg, (4) 0.0625 μg. Vimentin peptides: (1) 5 μg, (2) 2.5 μg, (3) 1.25 μg, (4) 0.625 μg.C
*In vitro* methyltransferase assays of vimentin wild‐type and 4RK mutant by PRMT5 analyzed by Western blots with anti‐sDMA antibody (right). Left: purified His‐vimentin proteins were stored in methylation reaction buffer, fractionated by SDS‐PAGE, and visualized by Coomassie blue stain. Data shown are representative of two independent experiments.DSingle‐cell tracking migration assays. CL1‐5 control and vimentin‐knockout cells were transfected with GFP vector or GFP‐vimentin for 48 h. Cells expressing GFP fluorescence were tracked by the time‐lapse video microscopy system. Representative trajectories and quantification of averaged velocity of cells (mean ± SD, Student *t* test, *n* = 20, technical replicates, *P < 0.05). 4RK indicates R196/207/345/364K; 4RF indicates R196/207/345/364F. Data shown are representative of three independent experiments.

To further identify the sDMA sites on vimentin in natural context, we utilized mass spectrometry to analyze endogenous vimentin immunopurified from CL1‐5, CL1‐0, and H1650 cells, and identified four dimethylated arginines, R196, R207, R345, and R364, on the coiled‐coil rod domain of vimentin (Figs 3A–E and EV3A). The dimethylated forms of the four arginine residues can be recognized by an anti‐sDMA antibody (Fig EV3B). To verify whether these four arginines are the key dimethylation sites of vimentin, these arginines were individually or entirely mutated to lysines (non‐arginine methylatable). Given that PRMT5 is the main enzyme responsible for sDMA production, PRMT5 was co‐transfected with wild type or mutant vimentin into HEK293T cells. As we expected, the sDMA level of wild‐type vimentin was enhanced by PRMT5, while R196K, R207K, R345K, and R364K mutants manifest varied reduction of vimentin dimethylation, and the dimethylation signal was dramatically diminished in the 4RK (R196/207/345/364K) quadruple mutant (Fig 3F). We also confirmed that these four arginines are the major targets in response to PRMT5‐mediated vimentin dimethylation by performing *in vitro* methyltransferase assays as the dimethylation signal of 4RK mutant was evidently attenuated (Fig EV3C).

To investigate if the functionality of vimentin is mediated by its sDMA level, endogenous vimentin in H1650 and CL1‐5 cells was knocked out by CRISPR/Cas9 approaches and the effect of wild‐type, 4RK, and 4RF (R196/207/345/364F, bulky hydrophobic form) mutant vimentin on cell motility was evaluated. Data from single‐cell tracking migration assays showed that the migration rate of vimentin‐knockout cells was reduced and rescued by restoration of wild‐type vimentin. Surprisingly, 4RF mutant failed to rescue cell migration, while 4RK mutant exhibited greater migration ability than wild‐type vimentin (Figs 3G and EV3D), indicating that the four arginine residues identified govern the migration promotion of vimentin.

### PRMT5‐mediated sDMA negatively regulates vimentin protein abundance

**Figure 4:**
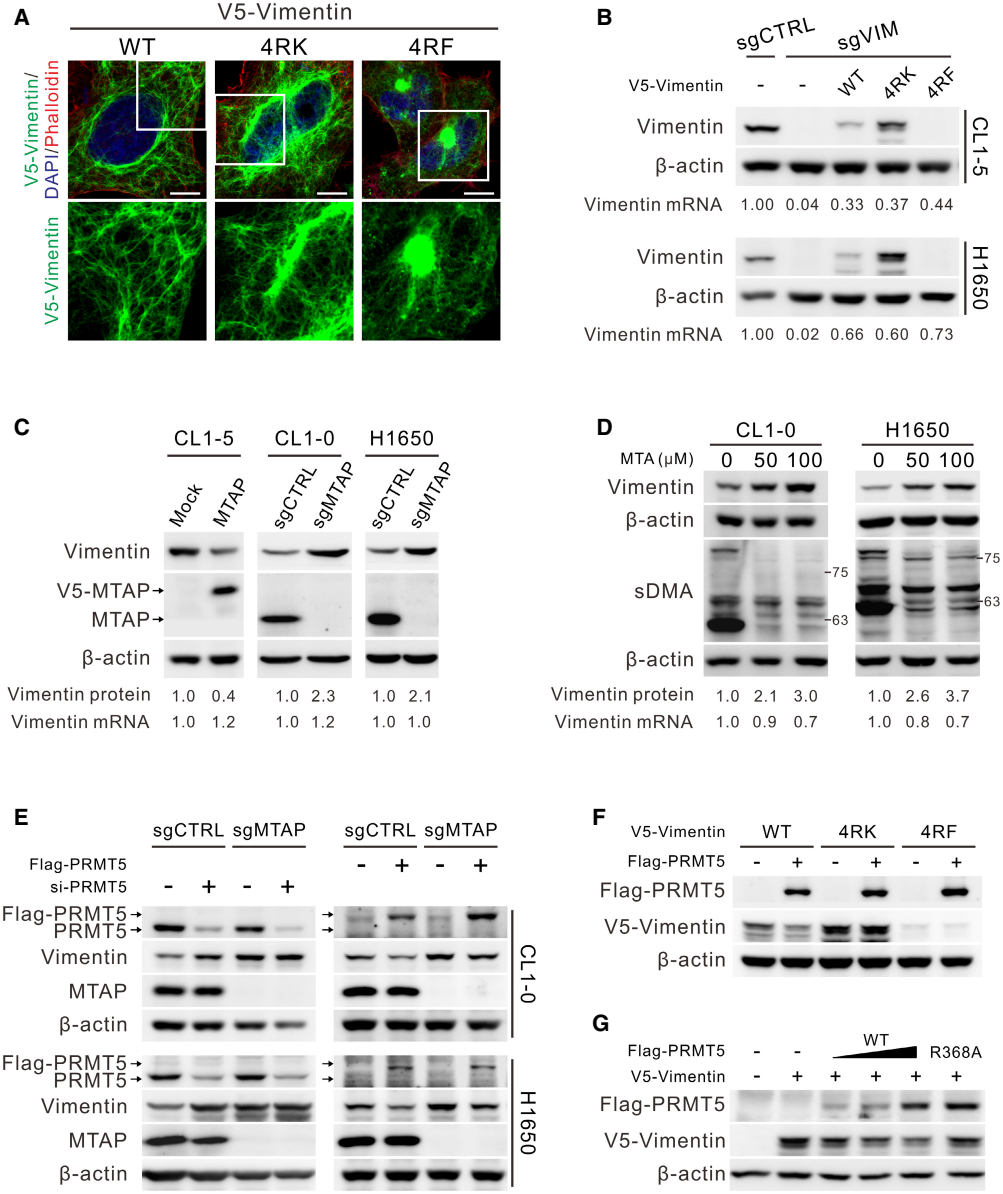
Vimentin protein level is upregulated by inhibition of PRMT5‐mediated sDMA AImmunofluorescence staining of V5‐vimentin wild‐type, 4RK and 4RF mutants expressed in CL1‐0 cells. F‐actin was stained with phalloidin, and nucleus was stained with DAPI. Scale bars, 10 μm. Data shown are representative of three independent experiments.BReconstitution of vimentin‐knockout cells with wild‐type, 4RK‐ and 4RF‐mutated V5‐vimentin. Both protein and mRNA levels of vimentin were determined by Western blots and RT‐qPCR. Data shown are representative of three independent experiments.CVimentin protein and mRNA levels in indicated paired cells were measured by Western blots and RT‐qPCR. Data shown are representative of three independent experiments.DEffect of MTA on vimentin protein and mRNA levels determined by Western blots and RT‐qPCR. Data shown are representative of three independent experiments.EWestern blot analysis of vimentin protein levels in MTAP‐knockout and control cells transfected with PRMT5 siRNAs (left) or Flag‐PRMT5 (right). Arrows mark the site of Flag‐PRMT5 and endogenous PRMT5. Data shown are representative of three independent experiments.FEffect of PRMT5 on the levels of ectopically expressed wild‐type, 4RK‐ and 4RF‐mutated V5‐vimentin in HEK293T cells assessed by Western blots. Data shown are representative of three independent experiments.GEffects of wild‐type and catalytically dead R368A mutant PRMT5 on the level of V5‐vimentin in HEK293T cells detected by Western blot assays. Data shown are representative of three independent experiments.

**Figure EV4:**
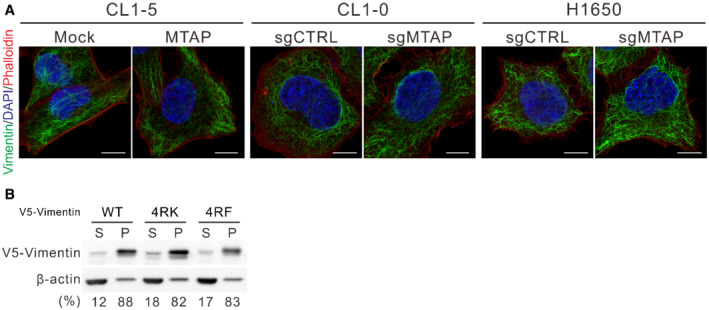
Effect of MTAP‐dependent sDMA on vimentin polymerization AImmunofluorescence staining of vimentin in indicated cell lines. F‐actin was stained with phalloidin, and nucleus was stained with DAPI. Scale bar, 10 μm. Data shown are representative of three independent experiments.BFractionation of soluble and insoluble V5‐vimentin. CL1‐0 cells were transiently expressed with V5‐vimentin wild‐type, 4RK and 4RF mutants for 48 h. The cells were fractionated to supernatants (S) and pellets (P). After pellets were resolved by sonication, equal portions of lysates were subjected to immunoblotting. The ratio of V5‐vimentin in the supernatant and pellet fractions was measured by ImageJ software. Data shown are representative of three independent experiments.

To gain insight into the effect of PRMT5‐mediated dimethylation on vimentin, immunofluorescence staining was utilized to observe the structure of vimentin cytoskeleton. The vimentin filament of MTAP‐overexpressing CL1‐5 cells and MTAP‐knockout CL1‐0 and H1650 cells seemed indistinguishable from their control cells (Fig EV4A). In cells ectopically expressing V5‐vimentin, wild‐type and 4RK mutant formed filaments except 4RF mutant with some aggregates (Fig 4A). We also examined vimentin solubility and found that the ratio of wild‐type and mutant vimentin in both supernatant and pellet fractions was not obviously different (Fig EV4B), suggesting that sDMA has a trivial effect on vimentin polymerization. Of note, the fluorescence intensity of vimentin in MTAP‐deficient cells or V5‐vimentin 4RK mutant was stronger than that in MTAP‐expressing cells or that of V5‐vimentin wild type, prompting us to measure the protein abundance by Western blots. The steady‐state protein level of 4RK mutant vimentin was more abundant than that of wild type, but 4RF mutant protein was almost undetectable even though its mRNA level was slightly higher than that of wild‐type and 4RK mutant vimentin (Fig 4B), supporting the notion that 4RF mutant vimentin fails to enhance cell motility (Fig 3G). Likewise, endogenous vimentin protein level in CL1‐5 mock cells and MTAP‐knockout cells of CL1‐0 and H1650 was more abundant than that in their isogenic cells (Fig 4C), explaining their morphological changes (Fig EV1G). Given no significant difference in vimentin mRNA expression between MTAP‐expressing and MTAP‐deficient cells (Fig 4C), we hypothesized that sDMA modification negatively regulates vimentin protein level.

To test this hypothesis, we manipulated intracellular sDMA levels by addition of MTA. Fig 4D showed accumulation of vimentin protein abundance upon a decrease of sDMA levels by increased MTA concentration. We also directly silenced the expression of PRMT5 by using PRMT5‐specific siRNAs and observed an elevation in vimentin protein in control cells, but the vimentin protein level was relatively insensitive to PRMT5 knockdown in MTAP‐knockout cells (Fig 4E, left), probably due to an existing inhibition of PRMT5‐mediated sDMA by accumulated MTA upon MTAP loss. In contrast, ectopic expression of PRMT5 led to a more significant drop of vimentin protein level in control cells than that in MTAP‐knockout cells (Fig 4E, right), supporting the importance of PRMT5 activity for vimentin protein regulation. To understand whether PRMT5 regulates vimentin protein level through R196/R207/R345/R364 methylation, we expressed V5‐vimentin of wild‐type, 4RK, or 4RF mutant alone or with Flag‐PRMT5 in cells and observed downregulation of wild‐type vimentin in response to PRMT5 overexpression, whereas mutant vimentin proteins seemed nearly unresponsive (Fig 4F). In contrast to PRMT5 wild‐type, the catalytically dead PRMT5 R368A mutant (Marjon *et al*, 2016) was unable to diminish vimentin protein level (Fig 4G). These results demonstrate that PRMT5‐mediated sDMA downregulates vimentin protein level.

### PRMT5‐mediated sDMA facilitates proteasomal degradation of vimentin

**Figure 5:**
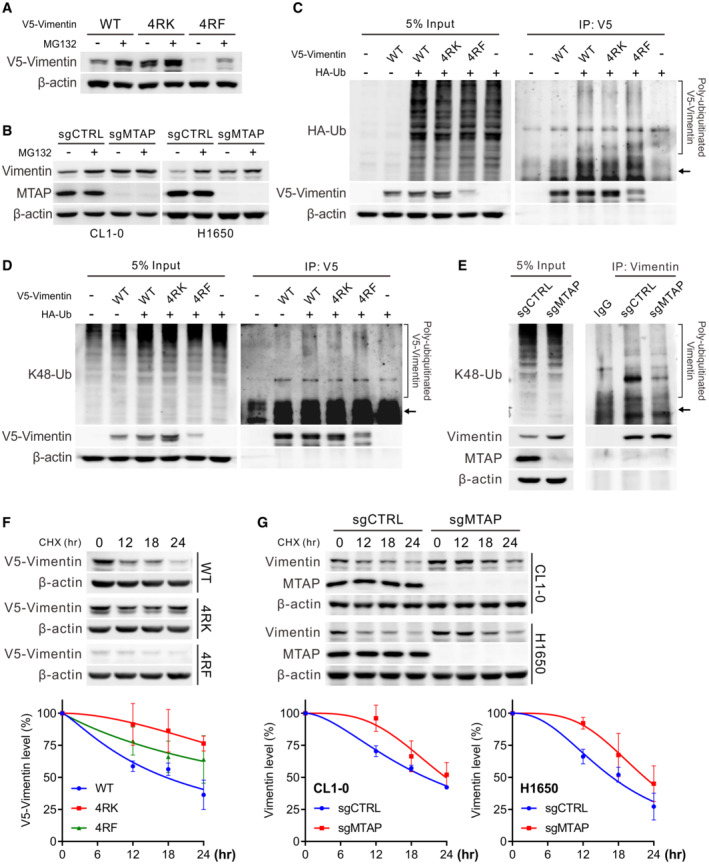
Reduced sDMA levels stabilize vimentin AMeasurement of V5‐vimentin wild‐type, 4RK and 4RF mutant protein levels in CL1‐5 vimentin‐knockout cells treated with 10 μM MG132 by Western blots. Data shown are representative of three independent experiments.BWestern blot analysis of vimentin protein levels in control and MTAP‐knockout cells treated with 10 μM of MG132. Data shown are representative of three independent experiments.C, DImmunoprecipitation analysis of ubiquitination (C) and K48‐linked polyubiquitination (D) of V5‐vimentin in HEK293T cells transfected with wild‐type, 4RK‐ or 4RF‐mutated vimentin in the presence of HA‐tagged ubiquitin. Arrows mark the sites of V5‐vimentin. Data shown are representative of three independent experiments.EImmunoprecipitation analysis for K48‐linked polyubiquitination of vimentin in CL1‐0 control and MTAP‐knockout cells. Arrow marks the site of vimentin. Data shown are representative of three independent experiments.F, GWestern blot analysis of V5‐vimentin wild‐type, 4RK and 4RF mutant levels in CL1‐0 cells (F) or endogenous vimentin levels in control and MTAP‐knockout cells (G) treated with 50 μg/ml cycloheximide (CHX). Bottom: the intensity of V5‐vimentin and vimentin signals was quantified by ImageJ software, normalized to the internal control β‐actin, then normalized to zero time point, and plotted against time points (mean ± SE, *n* = 3, biological replicates).

**Figure EV5:**
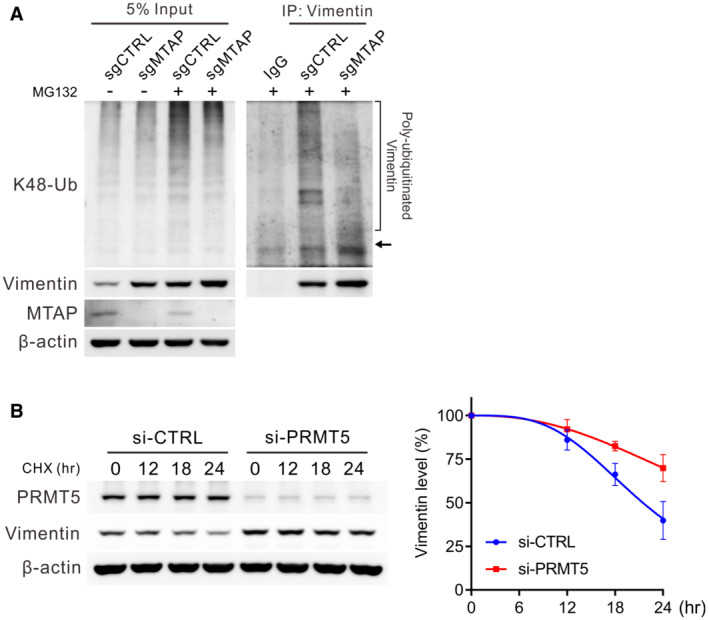
Effect of sDMA on vimentin ubiquitination and protein stability AImmunoprecipitation analysis for K48‐linked polyubiquitination of vimentin in CL1‐0 control and MTAP‐knockout cells pretreated with 10 μM of MG132 for 3 h. Arrow marks the site of vimentin. Data shown are representative of three independent experiments.BWestern blot analysis of endogenous vimentin levels in H1650 control and PRMT5‐silencing cells treated with 50 μg/ml cycloheximide (CHX). Right: the intensity of vimentin signals was quantified by ImageJ software, normalized to the internal control β‐actin, then normalized to zero time point, and plotted against time points (mean ± SE, *n* = 4, biological replicates).

To clarify whether sDMA‐mediated vimentin protein downregulation is through degradation pathways, cells were first treated with a proteasome inhibitor, MG132. Western blots demonstrated that the protein expression level of V5‐vimentin wild‐type was rescued to the similar extent as 4RK mutant, and 4RF mutant was also partially restored (Fig 5A). Simultaneously, the abundance of vimentin protein in MTAP‐expressing control cells was also mostly rescued comparable to that in MTAP‐knockout cells by pretreatment with MG132 (Fig 5B), implying that sDMA modification promotes vimentin toward degradation. Next, we examined the ubiquitination level of immunoprecipitated V5‐vimentin co‐expressed with HA‐ubiquitin in HEK293T cells by Western blot assays. The ubiquitination level of 4RK mutant was less than wild‐type vimentin, while less amount of precipitated 4RF mutant showed similar ubiquitination level to that of wild‐type vimentin (Fig 5C). A similar pattern regarding K48‐linked polyubiquitination, the specific tag for proteins toward proteasomal degradation, was also observed in V5‐vimentin mutants (Fig 5D). In MTAP‐knockout cells, the less dimethylated vimentin was conjugated with less K48‐linked polyubiquitin chains as compared to that in control cells (Fig 5E); strikingly, the difference in K48‐linked ubiquitination between control and MTAP‐knockout cells was enhanced in the presence of MG132 treatment (Fig EV5A), suggesting that sDMA modification potentiates vimentin to undergo ubiquitination and proteasome‐mediated degradation. The vimentin protein degradation was next monitored after cells were treated with cycloheximide. As shown in Fig 5F, the unmethylatable 4RK mutant vimentin had a prolonged half‐life compared with wild‐type and 4RF mutant. In PRMT5‐silencing and MTAP‐knockout cells, the less dimethylated vimentin protein was more stable than that in control cells (Figs. 5G and EV5B). In sum, these data suggest PRMT5‐mediated sDMA modification negatively regulates the stability of vimentin, and the stabilized vimentin compels the invasion of MTAP‐deleted cancer.

### MTAP/PRMT5 axis is inversely associated with vimentin protein level in lung cancer

**Figure 6:**
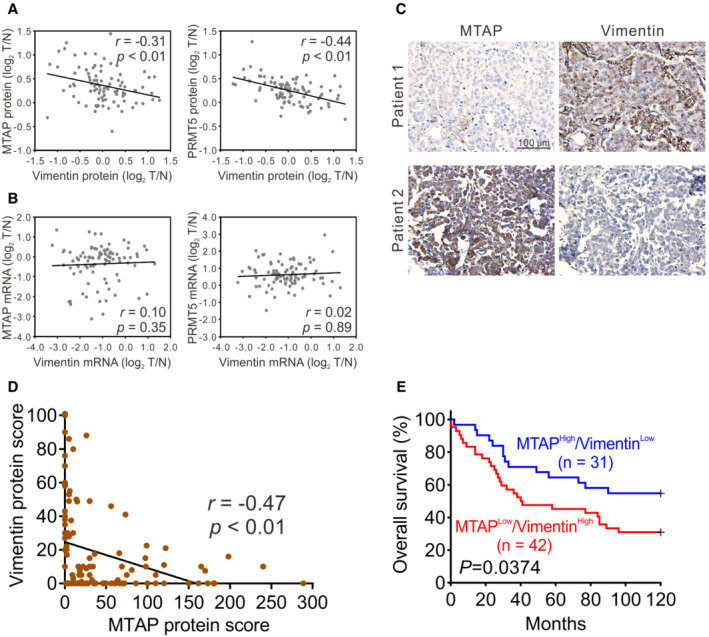
Negative association between MTAP/PRMT5 and vimentin is confirmed in lung cancer patients A, BThe correlation of MTAP and vimentin or PRMT5 and vimentin at protein (A) and mRNA (B) levels from 89 lung adenocarcinoma patients. *r* is the Spearman's rank correlation coefficient.CRepresentative images of IHC staining.DAn association between MTAP and vimentin levels in 124 lung cancer patients was examined by Spearman's rank correlation.EKaplan–Meier analyses of overall survival for lung cancer patients grouped by MTAP and vimentin levels from IHC staining. *P* value was obtained by log‐rank test.

This data support our hypothesis in which MTAP downregulates vimentin protein abundance by elevating PRMT5‐mediated sDMA levels. To further address the clinical relevance of this hypothesis, we first utilized our proteomic and transcriptomic data from a prospectively collected lung adenocarcinoma cohort (Appendix Table S6) of Taiwan Cancer Moonshot (Chen *et al*, 2020) to examine the correlation between MTAP/vimentin and PRMT5/vimentin. Consistent with the *in vitro* cell line data, vimentin protein level was negatively associated with MTAP or PRMT5 expression (Fig 6A), and their mRNA expression levels showed no correlations (Fig 6B). Moreover, the negative correlation of PRMT5 and vimentin was stronger than the negative association between MTAP and vimentin, supporting the cascade of MTAP‐PRMT5‐vimentin signaling pathway. Furthermore, immunohistochemical (IHC) staining of commercial tissue microarrays with survival data were performed to be another validation cohort (Fig 6C). The clinical characteristics of 124 lung cancer patients were summarized in Appendix Tables S7 and S8. In agreement with the aforementioned results, a negative association between MTAP and vimentin was observed in 124 lung cancer patients (Fig 6D). One hundred and twenty‐one patients in this cohort with survival data were further divided into high‐ and low‐expression categories and the Kaplan–Meier survival analyses further demonstrated that compared to those patients with high MTAP/low vimentin levels, patients with low MTAP/high vimentin levels were associated with worse overall survival (Fig 6E). These results indicate the significance of MTAP/PRMT5/vimentin axis in the regulation of lung cancer progression and metastasis.

## Discussion

**Figure 7:**
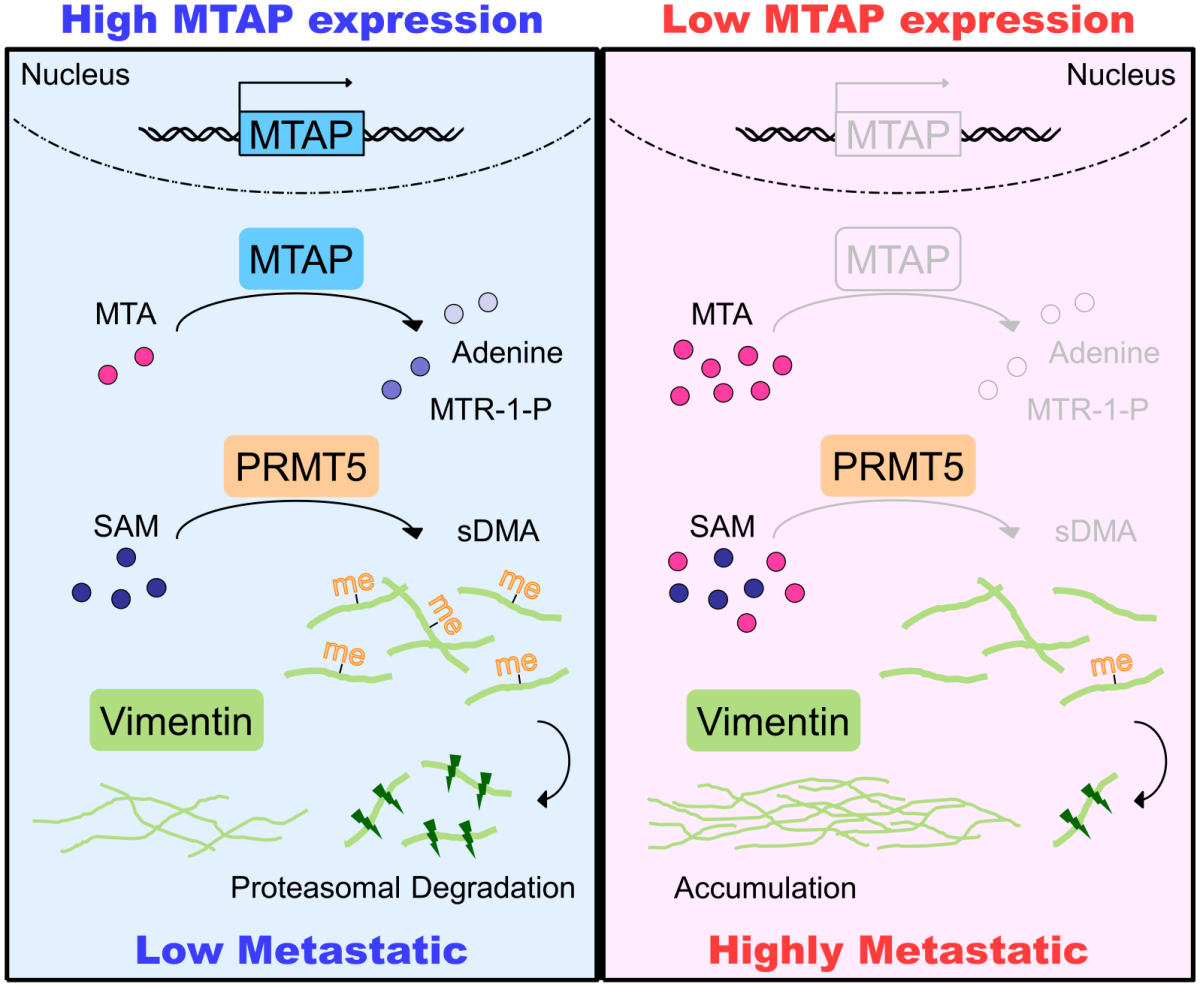
Hypothetical model for the mechanism of MTAP‐mediated suppression of lung cancer metastasis Our invasion cell‐based model demonstrated that in high MTAP‐expressing low metastatic cancer cells, MTA level is relatively low, facilitating PRMT5 to dimethylate vimentin, leading to vimentin degradation. Conversely, in highly metastatic cancer cells with MTAP loss, accumulated MTA inhibits PRMT5 activity so that low dimethylated vimentin is stabilized and responsible for lung cancer metastasis.

This study elucidates the mechanism underlying MTAP‐mediated metastasis (Fig 7). We first characterized the significance of MTAP abundance and activity in metastasis suppression. Next, we profiled the MTAP/MTA/PRMT5‐mediated methylproteome, and identified vimentin as a novel symmetrically dimethylated protein modulated by MTAP and PRMT5 activity and as the functional effector of MTAP‐loss‐induced invasion. Moreover, the MTAP‐ and PRMT5‐mediated sDMA modification negatively regulates the stability of vimentin protein via proteasomal degradation, as evidenced by the observations from two independent cohorts, Taiwan Cancer Moonshot proteogenomic data and tissue microarray IHC staining data.

Genome instability, metabolic reprogramming, and activating metastasis are three of the cancer hallmarks, and the interplay between each hallmark facilitates tumor progression and metastatic dissemination (Hanahan & Weinberg, 2011). *MTAP* gene is often found deleted in various cancer types and not necessarily co‐deleted with *CDKN2A* (Bertino *et al*, 2011; Su *et al*, 2014; Kryukov *et al*, 2016; Woollard *et al*, 2016). The physiological role of MTAP is to metabolize MTA (Bertino *et al*, 2011) and execute tumor suppressive functions (Christopher *et al*, 2002; Xu *et al*, 2019) including metastasis inhibition in this study, suggesting that MTAP contributes to cancer hallmarks from many aspects and serves as a promising prognostic biomarker. Unfortunately, tumor suppressor gene abnormalities are not currently clinically actionable, justifying the development of therapeutic strategies for targeting the vulnerability in MTAP‐deficient cells. We previously uncovered that MTAP‐deficient renal carcinoma cells become addicted to IGF1R activity to drive oncogenic pathways, so the selective IGF1R inhibitor preferentially impairs their viability and invasiveness (Xu *et al*, 2019). In this study, we verify the significance of vimentin in metastasis promotion of MTAP‐deficient lung cancer. MTAP was found to have an inhibitory effect on the cell invasion of hepatocellular carcinoma (HCC) (Hellerbrand *et al*, 2006), and previous studies on proteome and methylproteome alterations triggered by MTAP overexpression further demonstrated vimentin protein downregulation and increased monomethylation on vimentin in HCC cells (Bigaud & Corrales, 2016). These observations suggest that the MTAP/PRMT5/vimentin axis includes but is not limited to lung cancer and provide a new therapeutic target against MTAP‐deleted tumors.

Metastases account for about 90% of cancer‐associated deaths (Lambert *et al*, 2017), showing the urgency of understanding pathways that drive cancer metastasis. We established an invasion cell model to identify metastasis‐related genes (Chu *et al*, 1997; Chen *et al*, 2001), and we discovered that *MTAP* is deleted during an increased invasive and metastatic potential, and the invasive ability of MTAP‐deleted cells is promoted by stabilized vimentin with less PRMT5‐mediated sDMA modification. It has been known that PRMT5 acts as oncogene to promote cell proliferation and invasion and is associated with worse survival in cancer patients (Xiao *et al*, 2019). PRMT5 coordinates invasive phenotypes by methylating H3R2me1 for transcriptional activation and H4R3me2s for transcriptional repression, leading to FGFR3 pathway activation (Jing *et al*, 2018) and TGFβ‐induced EMT (Chen *et al*, 2017) in lung cancer cells. Surprisingly, our current work has confirmed that PRMT5 is involved in MTAP‐mediated metastatic suppression, contradictory to the general understanding of PRMT5. We have shown that vimentin protein is upregulated when sDMA level is inhibited by MTA. Direct manipulations of PRMT5 level by either silencing or overexpression lead to inverse expression level of vimentin. Moreover, PRMT5 downregulates wild‐type vimentin only but not 4RK methylation‐resistant mutant. Ectopic expression of catalytically dead PRMT5 cannot diminish vimentin protein abundance. Convincingly, a negative correlation between PRMT5 and vimentin is also noted in clinical lung specimens. Since PRMT5 acts as the master regulator of sDMA and governs multi‐processes depending on the substrates it methylates, the influence of PRMT5 in tumor progression might be dissimilar in different spatiotemporal situations.

Post‐translational modifications (PTMs) have been demonstrated to participate in many cellular biochemical and physiological processes, and therapeutic targeting of specific PTMs that drive oncogenic signaling aids in combating the cancer malignancy (Wu *et al*, 2019). Recent studies have bridged the relationship between PTMs and metabolites, especially those acting as donor for functional group transfer including acetyl‐CoA for acetylation, S‐adenosylmethionine (SAM) for methylation, and uridine diphosphate N‐acetylglucosamine (UDP‐GlcNAc) for glycosylation (Campbell & Wellen, 2018). Cancer cells exhibit metabolic reprogramming to overcome challenges within the tumor microenvironment, and the aberrant metabolite abundance leads to the alteration of PTMs on proteins that allow cancer cells to survive and progress. On the other hand, nutrient‐responsive PTMs on proteins or chromatin modulate the expression of genes and proteins involved in metabolism, forming a feedback or feedforward loop (Campbell & Wellen, 2018). In this study, we found that MTA accumulation in MTAP‐deleted cells restrains PRMT5‐mediated sDMA on vimentin and other proteins. Clinically, MTAP is highly frequently deleted, and MTA is significantly elevated in lung adenocarcinoma tissues compared to matched nonmalignant tissues (Wikoff *et al*, 2015; Fahrmann *et al*, 2017), highlighting the potential of MTAP/MTA as diagnostic markers and the unmet needs to explore the metabolomics and PTMomics of MTAP‐loss cancers for targeted therapy development.

In addition to the interplay with metabolites, the crosstalk between PTMs also influences the signal transduction and biological functions. Recently, there are a number of studies focusing on protein methylation‐dependent ubiquitination and degradation. After being methylated by methyltransferase, the stability of substrate proteins would be either upregulated or downregulated, thereby strengthening or alleviating the involved pathways and functions such as alterations in lipogenesis, angiogenesis, metastasis, genome stability, and carcinogenesis (Hu *et al*, 2015; Kim *et al*, 2016; Liu *et al*, 2016, 2020; Leng *et al*, 2018; Li *et al*, 2020). Therefore, protein methylation appears to be a new hallmark for protein stability. How can methylation modification be a signal for protein degradation? Since PTMs can lead to protein conformational change, the interaction accessibility of methylated proteins to E3 ligases or deubiquitinases would be altered (Yang *et al*, 2009). Two E3 ligases, TRIM16 and RNF208, were previously reported as negative regulators of vimentin (Marshall *et al*, 2010; Pang *et al*, 2019; Tian *et al*, 2020), but it is unclear whether their interaction with vimentin would be interfered by sDMA modification. Moreover, their targeting sites for ubiquitin conjugation on vimentin is outside of the coiled‐coil rod domain, which is the major dimethylated region and responsible for arginine methylation‐dependent ubiquitination in this study. The detailed regulation mechanisms could be elucidated by exploring the ubiquitome in the context of wild‐type or 4RK vimentin.

PRMT5, the major type II PRMT, plays an indispensable role in the regulation of developmental and physiological processes as the global deletion of PRMT5 in mice results in embryonic lethality (Guccione & Richard, 2019). Overexpression or elevation in enzymatic activity of PRMT5 has been observed in various types of cancers, exacerbating the progression of tumors (Xiao *et al*, 2019). Based on these reports, PRMT5 seems an attractive therapeutic target in disease treatment and there are several potential selective inhibitors undergoing clinical trials (Li *et al*, 2019). Nevertheless, most inhibitors designed to alter PTM signaling are against PTM writers (e.g. kinases, acetyltransferases, methyltransferases) but not PTM readers or substrates. A PTM writer coordinates numerous signaling pathways and works on diverse types of substrate proteins simultaneously, so general inhibition of an upstream PTM writer may also hinder other desired biological processes, thereby bringing in unwanted adverse effects. For example, PRMT5 is also crucial for the maintenance of invariant natural killer T cells, CD4^+^ T cells, and CD8^+^ T cells (Inoue *et al*, 2018). PRMT5 inhibitors might exert beneficial effects on tumor cytotoxicity (Kim *et al*, 2020) but also disrupt the tumor immunosurveillance (Strobl *et al*, 2020), possibly explaining why current PRMT5 inhibitors have no selectivity for MTAP‐loss tumors but instead are broadly anti‐proliferative. Herein, we provide another possibility to address this issue. Under the condition of MTA accumulation, PRMT5 is less active but there are still symmetrically dimethylated proteins in MTAP‐knockout cells (Fig 2B), implying that either PRMT5 possesses minimal activity or other type II PRMTs compensate the role of PRMT5 to execute methylation on certain substrate proteins that facilitate cellular growth and survival in the context of MTAP deletion. Identification of those symmetrically dimethylated proteins with reduced sDMA levels required for the malignancy of MTAP‐deleted cancers may provide an opportunity for tailored cancer therapy. For a proof of concept, we demonstrated that less dimethylated vimentin drives the metastasis of MTAP‐knockout cells, being an attractive therapeutic target.

The intermediate filament vimentin is widely used as a canonical marker of EMT and associated with cancer metastasis (Strouhalova *et al*, 2020). Metastasis is a multiphase process, and generally considered to be initiated by EMT of neoplastic cells. Conventionally, EMT activation is accompanied by reprogramming of gene expression in which EMT‐activating transcription factors (EMT‐TFs, such as SNAIL1/2, ZEB1/2 and TWIST1/2) repress the expression of epithelial markers and raise the expression of mesenchymal markers, leading to morphological change and metastasis (Strouhalova *et al*, 2020). Intriguingly, we noticed that MTAP negatively regulates vimentin abundance, further inhibiting EMT and metastasis. This EMT regulation is possibly through PTM‐mediated protein stability, not through canonical EMT‐TFs transcriptional regulation. The critical role of sDMA modification for vimentin in regulating EMT is, we believe, a particularly novel paradigm. While vimentin has been known to be functionally regulated by several PTMs (Snider & Omary, 2014), this is the first demonstration, to our knowledge, that vimentin is substantiated to be symmetrically dimethylated by PRMT5 and impaired its protein stability. Owing to the diverse role of vimentin in regulating cell adhesion, polarization, stiffness, and cell‐extracellular matrix interaction (Strouhalova *et al*, 2020), whether sDMA modification of vimentin affects these cellular functions and intracellular signaling needs further detailed investigations. The importance of vimentin in cancer progression also makes it an attractive druggable target. Withaferin A, a plant‐derived drug, was found to inhibit tumor growth and have pro‐apoptotic activity by directly binding vimentin, but it has also been shown to potently inhibit a variety of proteins (Bargagna‐Mohan *et al*, 2007; Thaiparambil *et al*, 2011; Hsu *et al*, 2019; Hassannia *et al*, 2020). Targeting cell‐surface vimentin (CSV) on tumor cells by monoclonal antibody 86C induces cancer cell apoptosis and CSV can serve as a biomarker to distinguish circulating tumor cells from other mesenchymal cells in blood of cancer patients (Satelli *et al*, 2015; Noh *et al*, 2016, 2018; Li *et al*, 2017; Batth *et al*, 2020), but the CSV antibody is currently for research use only. Taken together, the discovery of potent vimentin‐targeting drugs is imperative in order to increase the translational potential of vimentin targeting as a therapy for cancers.

In summary, we elucidate the mechanism of MTAP deficiency‐driven metastasis and define the significance of a novel post‐translational modification (symmetric dimethylation) on vimentin. Moreover, the approach that recognizes differentially symmetrically dimethylated proteins by methylproteomic screening seems a promising strategy to identify potential therapeutic targets and to address the unmet needs of combating MTAP‐deficient cancers. The remaining dimethyl proteins also merit further investigation for the development of effective precision diagnosis and treatment for lung cancer.

## Materials and Methods

### Reagents and antibodies

RPMI‐1640 medium, Dulbecco's modified Eagle's medium (DMEM), fetal bovine serum and penicillin–streptomycin were purchased from Life Technologies (Carlsbad, CA). Lipofectamine 2000, TRITC‐conjugated phalloidin, anti‐V5 and Alexa 488‐conjugated secondary antibodies were purchased from Invitrogen (Carlsbad, CA). VECTASTAIN® Elite ABC Kit (Rabbit IgG), VECTOR® Hematoxylin QS nuclear counterstain, and DAB solution were purchased from VECTOR Laboratories Inc. (Burlingame, CA). 5’‐Deoxy‐5′‐(methylthio)adenosine (MTA) was purchased from Sigma‐Aldrich (St Louis, MO). Anti‐vimentin (V9) antibody, goat anti‐mouse IgG‐HRP, and goat anti‐rabbit IgG‐HRP secondary antibodies were purchased from Santa Cruz Biotechnology (Santa Cruz, CA). Anti‐vimentin (#VIM‐V9‐L‐CE) antibody for immunohistochemical staining was purchased from Leica Biosystems (Wetzlar, Germany). Anti‐MTAP (2G4) antibody was purchased from Abnova (Taipei, Taiwan). Cyclohexamide (CHX) and anti‐monomethylarginine (mMA) (D5A12) antibody were purchased from Cell Signaling Technology (Danvers, MA). Anti‐β‐actin (GT5512) antibody was purchased from GeneTex (Hsinchu, Taiwan). Anti‐PRMT5 (#07–405), anti‐HA (#05–904), anti‐Flag (2EL‐1B11), anti‐α‐tubulin (DM1A), anti‐Lys48‐Specific Ubiquitin (K48‐Ub) (Apu2), and anti‐dimethylarginine, symmetric (sDMA) (SYM10) and asymmetric (aDMA) (ASYM25) antibodies were purchased from Merck Millipore (Burlington, MA).

### Plasmid constructs and primers

pcDNA3.1/V5‐His TOPO‐MTAP wild‐type and D220A mutant as well as pcDNA3.1/3xHA‐ubiquitin and LentiCRISPRv2‐sgMTAP for MTAP knockout have been previously described (Chen *et al*, 2014; Xu *et al*, 2019). The plasmid pET‐21b(+)‐vimentin wild‐type was kindly provided by Dr. Hong‐Chen Chen (Yang *et al*, 2019). For generation of V5‐tagged and GFP‐tagged vimentin, VIM coding region was amplified by PCR from cDNAs of CL1‐5 cells using the forward primer: 5′‐GGATCCATGTCCACCAGGTCCGTGTCCTCG‐3′, which introduced a BamHI site, and the reverse primer: 5′‐TCTAGACTTTCAAGGTCATCGTGATGCTGAG‐3′, which introduced a XbaI site. The amplified product was cloned into the constitutive mammalian expression vector pcDNA3.1/V5‐His TOPO and pEGFP‐C1. The resulting plasmids were then fully sequenced to ensure that no mutations were introduced during the PCR amplification. The vimentin mutants were generated using site‐directed mutagenesis by overlapping extension using PCR with mutagenic primers. The primer sequences for site direct mutagenesis are listed as follows: R196K forward: 5′‐G GAG ATG CTT CAG AAA GAG GAA GCC‐3′ and reverse: 5’‐TTC GGC TTC CTC TTT CTG AAG CAT C‐3′; R196F forward: 5′‐G GAG ATG CTT CAG TTT GAG GAA GCC‐3′ and reverse: 5’‐TTC GGC TTC CTC AAA CTG AAG CAT C‐3′; R207K forward: 5’‐CTG CAA TCT TTC AAA CAG GAT GTT G‐3′ and reverse: 5’‐GTC AAC ATC CTG TTT GAA AGA TTG C‐3′; R207F forward: 5’‐CTG CAA TCT TTC TTT CAG GAT GTT G‐3′ and reverse: 5’‐GTC AAC ATC CTG AAA GAA AGA TTG C‐3′; R345K forward: 5′‐GAA CGC CAG ATG AAA GAA ATG GAA G‐3′ and reverse: 5′‐GTT CTC TTC CAT TTC TTT CAT CTG‐3′; R345F forward: 5′‐GAA CGC CAG ATG TTT GAA ATG GAA G‐3′ and reverse: 5′‐GTT CTC TTC CAT TTC AAA CAT CTG‐3′; R364K forward: 5’‐CAA GAC ACT ATT GGC AAA CTG CAG G‐3′ and reverse: 5′‐AT CTC ATC CTG CAG TTT GCC AAT AG‐3′; R364F forward: 5’‐CAA GAC ACT ATT GGC TTT CTG CAG G‐3′ and reverse: 5′‐AT CTC ATC CTG CAG AAA GCC AAT AG‐3′. The resulting mutations were confirmed by Sanger DNA sequencing. PRMT5 cDNA was derived from pLVX‐IRES‐blasti‐PRMT5 kindly provided by Dr. Kevin M. Marks (Marjon *et al*, 2016) and subcloned into pCMV‐Tag 2B expression vector with EcoRI and XhoI cutting sites. For generation of sgRNA for VIM knockout, the insert oligonucleotides, oligo 1: 5′‐CACCGCGCAGCCTTACTTCTCCCGG‐3′ and oligo 2: 5′‐AAACCCGGGAGAAGTAAGGCTGCGC‐3′, were synthesized, annealed, and cloned into the LentiCRISPRv2 expression vector according to the manufacturer's protocol.

### Cell culture and transfection

The low invasive and highly invasive human lung adenocarcinoma cell lines, CL1‐0 and CL1‐5, were established and characterized as previously described (Chen *et al*, 2001). HEK293T, A549, H1755, H1437, H1650, H1568, and H1975 cells were purchased from American Type Culture Collection (ATCC). NCI‐H322M, NCI‐H23, EKVX, HOP‐62, and NCI‐H522 cells were purchased from the National Cancer Institute's Developmental Therapeutics Program (NCI, Bethesda, MD). All were authenticated and tested for mycoplasma contamination, and passaged in our laboratory for fewer than 6 months after receipt. Cells were cultured in the recommended medium at 37°C in a humidified atmosphere of 5% CO_2_. For ectopic expression of V5‐tagged MTAP in MTAP‐deleted cells, CL1‐5, A549, and H322M cells were transfected with pcDNA3.1/V5‐His TOPO‐MTAP wild‐type, pcDNA3.1/V5‐His TOPO‐MTAP D220A mutant, or pcDNA3.1/V5‐His TOPO vector using Lipofectamine 2000 reagent (Invitrogen, Carlsbad, CA), according to the manufacturer's protocol. CL1‐5 transfectants were further selected in medium containing 800 μg/ml G418 (Sigma‐Aldrich, St Louis, MO) for 2–3 weeks, and maintained in medium containing 500 μg/ml G418 for further investigation. For establishment of MTAP‐ or VIM‐knockout stable cell lines, we utilized lentiviruses that were generated by co‐transfection of HEK293T cells with the appropriate sgRNA‐containing lentiviral vector and a packing DNA mix, using Lipofectamine 2000. Cells were infected at three different Multiplicities of Infection (MOIs) in polybrene (8 μg/ml)‐containing medium. Twenty‐four hours after infection, the cells were treated with 2 μg/ml puromycin and puromycin‐resistant clones were selected and sequenced to confirm the gene‐editing results. For siRNAs transfection, SMARTpool of ON‐TARGETplus VIM and PRMT5 siRNAs and control siRNA (Dharmacon, Lafayette, CO) were transfected using DharmaFECT reagent according to the manufacturer's protocol.

### Real‐time quantitative RT‐PCR

Total RNAs from the tumor specimens and cultured cell lines were extracted by the TRIzol reagent (Life Technologies, Carlsbad, CA), and 1 μg of total RNAs was used in cDNA synthesis with random hexamer primers using Superscript III reverse transcriptase (Life Technologies, Carlsbad, CA). 20 ng RNA or 10 ng cDNA were served as the template for mRNA expression detection in real‐time quantitative RT‐PCR on ABI prism 7900 sequence detection system (Life Technologies, Carlsbad, CA). For TaqMan real‐time quantitative RT‐PCR, the primers sets for MTAP (Hs00559618_m1) and the internal control, TBP (Hs00427621_m1), were purchased from Life Technologies (Carlsbad, CA). For SYBR Green real‐time quantitative RT‐PCR, the primers used in this study were described below. The relative mRNA expression of target genes was determined as −∆CT = −[CT_target_−CT_TBP_]. The target/TBP mRNA ratio was calculated as 2^−∆CT^ × K, in which K is a constant. All experiments were performed in triplicate.

MTAP forward primer: 5′‐CAA TGG CTG AGC CGT TTT G‐3′; MTAP reverse primer: 5′‐GAT TGT GAC CAT TGT CCC CTT T‐3′; VIM forward primer: 5′‐GAG AAC TTT GCC GTT GAA GC‐3′; VIM reverse primer: 5′‐GCT TCC TGT AGG TGG CAA TC‐3′; TBP forward primer: 5′‐CAC GAA CCA CGG CAC TGA TT‐3′; TBP reverse primer: 5′‐TTT TCT TGC TGC CAG TCT GGA C‐3′.

### Western blot and immunoprecipitation assays

The preparations of whole‐cell lysates and immunoblotting analyses have been previously described (Chang *et al*, 2016;Chen *et al*, 2016; Xu *et al*, 2019). For immunoprecipitation analysis, cells were first lysed in lysis buffer (50 mM of Tris–HCl (pH 7.4), 1% NP‐40, 150 mM of NaCl, 1 mM of EDTA) containing protease and phosphatase inhibitor cocktails (Roche, Basel, Switzerland), and the lysates were cleaned by preincubation with Protein A/G PLUS‐Agarose beads (Santa Cruz Biotechnology, Santa Cruz, CA) to remove nonspecifically bound proteins. After precipitation with the appropriate antibodies and Protein A/G PLUS‐Agarose beads, the immunoprecipitated complexes were washed, separated by SDS‐PAGE and followed by Western blot assays. Immunoblotting was conducted with appropriate antibodies, visualized by chemiluminescence assay kit (Merck Millipore, Burlington, MA), and detected by FUJIFILM LAS‐3000 ECL system.

### *In vitro* methyltransferase assay

In each reaction, 0.375 μg of recombinant human PRMT5 (SRP0145, Sigma‐Aldrich), 0.5 μg of recombinant His‐tagged vimentin, 160 μM of methyl donor S‐adenosylmethionine (SAM, B9003S, New England Biolabs), and 160 μM of 5’‐Deoxy‐5′‐(methylthio)adenosine (MTA) were incubated in methylation reaction buffer (40 mM of Tris–HCl (pH 8.0), 110 mM of NaCl, 2.2 mM of KCl, 3 mM of dithiothreitol (DTT)) for 90 min at 37°C in 30 μl volume. Reactions were terminated with the addition of 10 μl of 4x Laemmli Sample Buffer. Arginine methylation of vimentin was detected by immunoblotting. For fluorography, an *in vitro* methyltransferase assay using radioactive SAM (NET155V250UC, Perkin Elmer) was also carried out as follows. One microgram of PRMT5 and 1 μg of substrate proteins (Histone H3, ab198757; Histone H4, ab198115; Abcam) were incubated in the presence of 3 μCi‐tritiated SAM (18.0 Ci/mmol from a 1.0 mCi/ml stock solution) for 1 h at 30°C in a final volume of 30 μl methylation reaction buffer. Subsequently, tritiated protein was separated by SDS‐PAGE and then the gel was treated with EN^3^HANCE (6NE9701, Perkin Elmer) and exposed to a film at −80°C for 7 days.

### Immunohistochemical staining

The formalin‐fixed and paraffin‐embedded specimens were performed immunohistochemical staining for analysis of the expression of MTAP and vimentin. Detailed experimental procedures were modified from the paraffin immunohistochemistry protocol supplied by the manufacturer (Cell Signaling, Danvers, MA). The slides were de‐paraffinized in xylene and rehydrated in graded alcohol and water. An antigen retrieval step (10 nM sodium citrate (pH 6.0) at a sub‐boiling temperature) was used for each primary antibody. Endogenous peroxidase activity was blocked by 3% hydrogen peroxide. The slides were blocked by serum and incubated with appropriate antibodies (MTAP, 1:100; vimentin, 1:200) overnight at 4°C. Detection of immunostaining was carried out by using the VECTASTAIN® ABC system, according to the manufacturer's instructions (Vector Laboratories, Burlingame, CA). The H‐score system was devised to confirm the relative expression of MTAP or vimentin in specimens (Chen *et al*, 2016; Xu *et al*, 2019). The final scores range from 0 to 300, and the specimens were classified into two groups according to the final scores. These results were also reviewed and scored independently by two pathologists.

### Immunofluorescent staining

Cells were first cultured on the 8‐well Lab‐Tek II chamber slides (Thermo Fisher Scientific, Waltham, MA) at 37°C for 48 h. The cells were fixed in PBS containing 4% paraformaldehyde and 2% sucrose for 15 min, permeabilized in PBS containing 0.1% Triton X‐100 and 1% BSA for 15 min, and blocked in PBS containing 3% BSA for 1 h. Then, the cells were stained with primary antibodies against vimentin, V5‐tag, and Alexa Fluor 488 conjugated anti‐mouse secondary antibody. F‐actin was stained with TRITC‐conjugated phalloidin, and nuclei were demarcated with DAPI staining. Fluorescent images were taken by a confocal microscope (Carl Zeiss LSM880, Oberkochen, Germany) with Airyscan detector and were processed by ZEISS ZEN2 image software.

### Invasion and single‐cell tracking migration assays

Transwell chambers (8 μm pore size; BD Biosciences, Franklin Lake, NJ) were utilized to perform *in vitro* invasion assays. First, upper chambers were coated with Matrigel (R&D Systems), and 2 × 10^4^ cells suspended in serum free medium were seeded onto the Matrigel‐coated upper chambers and 1 ml medium containing 10% FBS was placed in the lower chambers. After 20 h of incubation, upper chambers were swabbed with a cotton swab, fixed with methanol, and then stained with Giemsa solution (Sigma‐Aldrich, St. Louis, MO). The cells attached to the lower surface of the chambers were counted under a light microscope. All experiments were assayed in triplicate.

For single‐cell tracking migration assays (Hsu *et al*, 2018), cells were seeded into CellCarrier‐96 Ultra Microplates (1,000 cells/well) (#6055302, PerkinElmer, Waltham, MA) and incubated overnight. Then, cells were stained with Cyto‐ID Red (ENZO Life Sciences, Plymouth Meeting, PA) and the cell movements were monitored at 30 min intervals using a high content screening system (Molecular Devices, Sunnyvale, CA). Video images were collected and stored as image stacks using MetaMorph software (Molecular Devices).

### Anchorage‐independent colony formation

A 6‐well plates were coated with a base layer of 1 ml of 0.7% agar (Agar Noble, BD Biosciences) in PBS. In the second layer, the cells were seeded in 1 ml of DMEM medium at a density of 500 cells per well supplemented with 10% FBS of 0.35% agar. Three milliliters of DMEM medium supplemented with 10% FBS were covered onto the second layer. After incubated at 37°C for 14 days, the cells were fixed with 4% paraformaldehyde, stained with 0.05% crystal violet and photographed. Colonies with a diameter greater than 1 mm were counted under an inverted microscope.

### Fractionation of soluble and insoluble vimentin

Cells were lysed at 4°C with a lysis buffer containing 1% Nonidet P‐40, 20 mM of Tris–HCl (pH 8.0), 137 mM of NaCl, 10% (v/v) glycerol, 1 mM of Na_3_VO_4,_ and protease inhibitor cocktails, and immediately centrifuged at 15,500 *g* in 4°C for 10 min. The resulting supernatant is soluble fraction. The insoluble pellet was suspended in RIPA lysis buffer (1% Nonidet P‐40, 50 mM of Tris–HCl (pH 7.4), 1% Na‐deoxycholate, 0.1% SDS, 2 mM of EDTA, 100 mM of NaF, 1 mM of Na_3_VO_4_) containing protease inhibitor cocktails, sonicated (30% power, 10 s pulses), and centrifuged at 15,500 *g* in 4°C for 10 min. Equal portions of soluble and insoluble fractions were then analyzed by Western blots (Yang *et al*, 2019).

### Patient tumor specimens

The first cohort of 101 patients who underwent surgical resection and with histologically confirmed LUAD were obtained from the Taichung Veterans General Hospital (Taichung, Taiwan) from January 2000 to September 2009. None of the patients had received neoadjuvant chemotherapy or radiation therapy. The second cohort of 124 lung tumor specimens were obtained from patients with histologically confirmed lung tumors who underwent surgical resection at the UC Davis Medical Center. None of the patients had received preoperative adjuvant chemotherapy or radiation therapy. These investigations were approved by the Institutional Review Board of the UC Davis Health System and Taichung Veterans General Hospital. Written informed consent was obtained from all patients.

### *In vivo* animal experiments

All mice experiments were approved by the Institutional Animal Care and Use Committee of National Taiwan University (No. 20160287). In each experiment, six‐week‐old NOD severe combined immunodeficiency (SCID) mice (supplied by BioLASCO, Taiwan) were housed four to six mice per cage and fed autoclaved food *ad libitum*. For tumorigenicity assay, 1 × 10^6^ cells were suspended in 100 μl of PBS and implanted subcutaneously into the dorsal region of mice. Tumor growth was examined thrice a week, and tumor volume was estimated by the formula *LW* ^2^/2, where *L* is the length and *W* is the width of the tumor. After 27 days, the mice were sacrificed and the tumor xenografts were removed, weighted, and photographed. For metastasis assay, 1 × 10^5^ cells were suspended in 100 μl of PBS and intravenously injected into the lateral tail vein of mice. All mice were sacrificed 10 weeks after injection, and the mouse lungs were removed and fixed in 10% formalin. The lung surface tumor foci were counted under a dissecting microscope. The mouse lungs were further embedded in paraffin, sectioned into 4 μm layers, and stained with hematoxylin–eosin for histological analysis. For orthotopic implantation assay, 1 × 10^5^ cells suspended in 10 μl of PBS mixed with Matrigel purchased from CORNING (#356231, Corning, NY) were inoculated into the left lungs of mice. After implantation of 28 days, the lungs of the mice were removed and fixed in 4% paraformaldehyde and the number of lung micrometastatic lesions was counted under a dissecting microscope. Embedded tissues were sliced and stained with hematoxylin–eosin for histological analysis.

### Identification of dimethyl‐proteins and sites by LC–MS/MS analysis

To identify differentially symmetrically dimethylated proteins, dimethyl‐proteins were immunoprecipitated from CL1‐5 Mock/MTAP, CL1‐0 sgCTRL/sgMTAP and H1650 sgCTRL/sgMTAP by anti‐sDMA antibody and the samples were subjected to SDS‐PAGE and Coomassie Brilliant Blue staining. Gel areas separated into 5 pieces by molecular weight in each sample were excised and destained, followed by reduction with final 5 mM of TCEP at 37°C for 30 min in the dark, alkylation in the dark with 20 mM of iodoacetamide at 37°C for 1 h, in‐gel trypsin digestion, and LC–MS/MS. The raw data were processed using Proteome Discoverer 2.1 (Thermo Fisher Scientific, Waltham, MA), and peptide identification was performed by search engines Mascot (Matrix Science, London, UK; version 2.3.2) and SEQUEST against the Swiss‐Prot database (v2015_12). The modifications including Carbamidomethylation at cysteine, oxidation at methionine, deamidation at asparagine or glutamine, and dimethylation at arginine were selected. To reduce false positive identification results, a decoy database containing the reverse sequences was appended to the database. Label‐free quantification was performed using the peak area of each precursor ion calculated from extracted ion chromatogram during data processing using the Precursor Ions Area Detector node with mass precision 2 ppm. The abundance of identified protein was calculated from the top three of all unique and razor peptides in Peptide and Protein Quantifier node, and was used to calculate the relative protein abundance between experimental samples. Proteins that were 2‐fold or more abundant in the CL1‐5 MTAP, CL1‐0 sgCTRL, or H1650 sgCTRL sample compared with CL1‐5 Mock, CL1‐0 sgMTAP, or H1650 sgMTAP sample were considered dimethyl‐protein candidates.

To identify the dimethyl site(s) on vimentin, vimentin was isolated by immunoprecipitation with anti‐vimentin antibody and subjected to SDS‐PAGE and Coomassie Brilliant Blue staining. The protein band corresponding to vimentin was excised and subjected to in‐gel digestion with trypsin. The peptides were extracted, desalted by C18 ziptip, and analyzed by LC–MS/MS. All MS/MS files were further processed by search engine Mascot against the Swiss‐Prot database (v2015_12). The common modifications including Carbamidomethylation at cysteine, oxidation at methionine, deamidation at asparagine, or glutamine were selected. In addition, the dimethyl with neutral losses 30.0344 or 44.0500 at arginine was set up to identify the symmetric and asymmetric dimethylation on vimentin, respectively. A decoy database containing the reverse sequences was appended to the database to reduce false positive identification results. The mass spectrometry proteomics data have been deposited to the ProteomeXchange Consortium via the PRIDE (Perez‐Riverol *et al*, 2022) partner repository with the dataset identifier PXD031192 and 10.6019/PXD031192.

### Statistical analysis

For *in vitro* or *in vivo* studies, the Student *t* test was used to compare the difference between two groups. Overall survival and progression‐free survival curves were calculated by the Kaplan–Meier analysis, and the log‐rank test was performed to test the difference between survival curves. Cox proportional hazards regression analysis was used to evaluate independent prognostic factors. Covariates of the regression model were MTAP, gender, age, and stage. The difference in patient characteristics between the high‐expression and low‐expression groups was examined using either Student *t* test or Chi‐square test. All analyses were performed using SPSS software (v23.0; SPSS, Inc., Chicago, IL) and GraphPad Prism 9 (GraphPad Software, San Diego, CA). All tests were two‐tailed, and *P* values < 0.05 were considered significant.

## Author contributions

**Wen‐Hsin Chang:** Conceptualization; data curation; formal analysis; validation; investigation; visualization; methodology; writing – original draft; writing – review and editing. **Yi‐Ju Chen:** Data curation; formal analysis; investigation; visualization; methodology. **Yi‐Jing Hsiao:** Formal analysis; validation; investigation; methodology. **Ching‐Cheng Chiang:** Formal analysis; validation; investigation. **Chia‐Yu Wang:** Formal analysis; validation; investigation. **Ya‐Ling Chang:** Investigation. **Qi‐Sheng Hong:** Investigation. **Chien‐Yu Lin:** Formal analysis. **Shr‐Uen Lin:** Investigation. **Gee‐Chen Chang:** Resources. **Hsuan‐Yu Chen:** Formal analysis. **Yu‐Ju Chen:** Resources; formal analysis. **Ching‐Hsien Chen:** Conceptualization; supervision; funding acquisition; writing – original draft; project administration; writing – review and editing. **Pan‐Chyr Yang:** Conceptualization; supervision; writing – original draft; project administration; writing – review and editing. **Sung‐Liang Yu:** Conceptualization; resources; supervision; funding acquisition; writing – original draft; project administration; writing – review and editing.

## Disclosure and competing interests statement

The authors declare that they have no conflict of interest.

## Supporting information



Appendix S1

Expanded View Figures PDF

Source Data for Expanded View

## Data Availability

The datasets produced in this study are available in the following databases:
Microarray data: Gene Expression Omnibus GSE160522 (https://www.ncbi.nlm.nih.gov/geo/query/acc.cgi?acc=GSE160522) Methylproteomic data: PRIDE PXD031192 (http://www.ebi.ac.uk/pride/archive/projects/PXD031192).
