# Supplementary material for: Reduced symmetric dimethylation stabilizes vimentin and promotes metastasis in MTAP‐deficient lung cancer
Source: EMBO Rep. 2022 Jun 29;23(8):e54265. doi: 10.15252/embr.202154265 (PMC9346486; doi:10.15252/embr.202154265)
Supplement: Supplementary file 1 — Appendix S1 [file EMBR-23-e54265-s002.docx]

**Reduced symmetric dimethylation stabilizes vimentin and promotes metastasis in MTAP-deficient lung cancer**

Wen-Hsin Chang, Yi-Ju Chen, Yi-Jing Hsiao, Ching-Cheng Chiang, Chia-Yu Wang, Ya-Ling Chang, Qi-Sheng Hong, Chien-Yu Lin, Shr-Uen Lin, Gee-Chen Chang, Hsuan-Yu Chen, Yu-Ju Chen, Ching-Hsien Chen, Pan-Chyr Yang, Sung-Liang Yu

**content**

Appendix Table S1.........page 2

Appendix Table S2.........page 3

Appendix Table S3.........page 4

Appendix Table S4.........page 5

Appendix Table S5.........page 6

Appendix Table S6.........page 16

Appendix Table S7.........page 17

Appendix Table S8.........page 18

**Appendix Table S1.** Clinicopathologic characteristics of 101 lung adenocarcinoma patients in relation to MTAP expression

| **Characteristic** | **Total patients** | **High MTAP**  Patient No. (%) | **Low MTAP**  Patient No. (%) | ***P* value** |
| --- | --- | --- | --- | --- |
| **Patient No.** | n=101 | n=61 | n=40 |  |
| **Age (meanSD)**  | 62.9±12.3 | 61.5±12.4 | 64.9±12.1 | 0.177^†^ |
| **Gender** |  |  |  | 0.213^‡^ |
| Male | 58 | 32 (52.46%) | 26 (65.00%) |  |
| Female | 43 | 29 (47.54%) | 14 (35.00%) |  |
| **Stage*** |  |  |  | 0.457^‡^ |
| I | 58 | 39 (65.00%) | 19 (48.72%) |  |
| II | 12 | 6 (10.00%) | 6 (15.38%) |  |
| III | 23 | 12 (20.00%) | 11 (28.21%) |  |
| IV | 6 | 3 (5.00%) | 3 (7.69%) |  |

^†^Student *t* test

^‡^Chi-square test

*Two patients without stage information

**Appendix Table S2.** Prognostic capability of MTAP on the overall survival of 101 lung adenocarcinoma patients

| **Cut-off** | **High MTAP** Patient No. | **Low MTAP** Patient No. | **Log-rank *P* value** |
| --- | --- | --- | --- |
| 20% | 80 | 21 | **0.0030** |
| 25% | 76 | 25 | **0.0112** |
| 30% | 71 | 30 | **0.0424** |
| 35% | 65 | 36 | **0.0194** |
| 40% | 61 | 40 | **0.0093** |
| 45% | 55 | 46 | **0.0479** |
| 50% | 50 | 51 | 0.2179 |
| 55% | 45 | 56 | 0.9237 |
| 60% | 40 | 61 | 0.5762 |
| 65% | 36 | 65 | 0.3672 |
| 70% | 30 | 71 | 0.7769 |
| 75% | 26 | 75 | 0.2877 |
| 80% | 20 | 81 | 0.2377 |

**Appendix Table S3.** Multivariate Cox regression analysis of MTAP expression and overall survival

| **Variable** | **Hazard Ratio (95% CI)** | ***P* value** |
| --- | --- | --- |
| High MTAP expression level | 0.53 (0.29-0.98) | 0.0422 |
| Male | 2.18 (1.03-4.61) | 0.0413 |
| Age | 1.00 (0.97-1.02) | 0.7302 |
| Late stage (stage III-IV) | 2.59 (1.37-4.89) | 0.0035 |

**Appendix Table S4.** Alteration of metabolite levels upon MTAP overexpression in CL1-5 cells

| **Metabolite** | **Fold change (MTAP/Mock)** | **Pathway** |
| --- | --- | --- |
| Phenylalanine | 2.44 | Amino acid |
| Tryptophan | 2.21 | Amino acid |
| Isoleucine | 1.40 | Amino acid |
| Leucine | 1.40 | Amino acid |
| Valine | 1.26 | Amino acid |
| Glutamic acid | 1.04 | Amino acid |
| Threonine | 0.98 | Amino acid |
| D-Serine | 0.55 | Amino acid |
| Alanine | 0.44 | Amino acid |
|  |  |  |
| Malic acid | 1.92 | TCA Cycle |
| Succinic acid | 0.70 | TCA Cycle |
|  |  |  |
| Aspartate | 1.53 | Urea Cycle |
| Citrulline | 0.87 | Urea Cycle |
|  |  |  |
| Taurine | 2.27 |  |
| 3-Hydroxybutyrate | 1.23 |  |
| Acetylcarnitine | 1.19 |  |
| Creatine | 1.13 |  |
| Trimethylamine-N-Oxide | 1.00 |  |
| Orotic acid | 0.90 |  |
| L-alpha-aminobutyric acid | 0.64 |  |
| Carnitine | 0.63 |  |

**Appendix Table S5.** Identification of differentially symmetrically dimethylated proteins

| Accession | Gene | Description | MW [kDa] | Fold in CL1-5^a^ | Fold in CL1-0^b^ | Fold in H1650^b^ | Arginine Methylation site |
| --- | --- | --- | --- | --- | --- | --- | --- |
| Q15149 | PLEC | Plectin | 531.5 | 4.6E+00 | 2.6E+07 |  |  |
| Q9Y520 | PRRC2C | Protein PRRC2C | 316.7 | 3.1E+00 | 4.3E+00 |  |  |
| Q13813 | SPTAN1 | Spectrin alpha chain, non-erythrocytic 1 | 284.4 | 8.8E+05 | 3.7E+07 |  |  |
| O75369 | FLNB | Filamin-B | 278.0 | 4.1E+00 | 4.7E+06 |  |  |
| O75643 | SNRNP200 | U5 small nuclear ribonucleoprotein 200 kDa helicase | 244.4 | 7.2E+00 | 9.7E+05 |  | Methyl [R427] |
| Q15154 | PCM1 | Pericentriolar material 1 protein | 228.4 | 2.9E+06 | 6.4E+07 |  |  |
| Q07157 | TJP1 | Tight junction protein ZO-1 | 195.3 | 3.3E+00 | 1.2E+01 |  |  |
| O43795 | MYO1B | Unconventional myosin-Ib | 131.9 | 3.8E+00 | 2.0E+00 |  |  |
| Q9UPN4 | CEP131 | Centrosomal protein of 131 kDa | 122.1 | 3.4E+00 | 7.4E+07 |  |  |
| Q9P0K7 | RAI14 | Ankycorbin | 110.0 | 6.7E+00 | 3.4E+00 |  |  |
| O43707 | ACTN4 | Alpha-actinin-4 | 104.8 | 3.5E+01 | 5.6E+07 |  |  |
| P43243 | MATR3 | Matrin-3 | 94.6 | 3.7E+00 | 1.7E+07 |  |  |
| P02768 | ALB | Serum albumin | 69.3 | 2.5E+00 | 1.7E+02 |  | Methyl [R452] |
| P27694 | RPA1 | Replication protein A 70 kDa DNA-binding subunit | 68.1 | 2.7E+07 | 5.7E+02 |  |  |
| Q6UWP8 | SBSN | Suprabasin | 60.5 | 7.6E+00 | 2.1E+07 |  |  |
| P04040 | CAT | Catalase | 59.7 | 4.0E+00 | 1.9E+07 |  | Methyl [R354] |
| P25705 | ATP5A1 | ATP synthase subunit alpha, mitochondrial | 59.7 | 5.1E+00 | 1.1E+07 |  |  |
| Q8WWY3 | PRPF31 | U4/U6 small nuclear ribonucleoprotein Prp31 | 55.4 | 2.7E+05 | 2.5E+02 |  |  |
| Q15233 | NONO | Non-POU domain-containing octamer-binding protein | 54.2 | 1.3E+01 | 2.7E+08 |  | Methyl [R251] |
| Q15517 | CDSN | Corneodesmosin | 51.5 | 3.3E+05 | 2.6E+00 |  |  |
| Q13748 | TUBA3C | Tubulin alpha-3C/D chain | 49.9 | 4.6E+07 | 1.6E+07 |  | Methyl [R79; R84] |
| P31943 | HNRNPH1 | Heterogeneous nuclear ribonucleoprotein H | 49.2 | 1.1E+07 | 6.9E+07 |  |  |
| O75635 | SERPINB7 | Serpin B7 | 42.9 | 1.7E+07 | 2.9E+07 |  |  |
| P51991 | HNRNPA3 | Heterogeneous nuclear ribonucleoprotein A3 | 39.6 | 6.1E+05 | 7.9E+01 |  |  |
| P04083 | ANXA1 | Annexin A1 | 38.7 | 2.4E+05 | 3.4E+06 |  |  |
| P22626 | HNRNPA2B1 | Heterogeneous nuclear ribonucleoproteins A2/B1 | 37.4 | 1.0E+06 | 2.0E+01 |  | Methyl [R190; R213] |
| P06748 | NPM1 | Nucleophosmin | 32.6 | 1.5E+06 | 4.1E+07 |  |  |
| Q6ZVX7 | NCCRP1 | F-box only protein 50 | 30.8 | 3.2E+06 | 4.2E+00 |  |  |
| P09234 | SNRPC | U1 small nuclear ribonucleoprotein C | 17.4 | 1.0E+06 | 1.2E+01 |  |  |
| P12273 | PIP | Prolactin-inducible protein | 16.6 | 2.5E+00 | 3.9E+00 |  |  |
| P06702 | S100A9 | Protein S100-A9 | 13.2 | 3.8E+06 | 8.4E+00 |  |  |
| Q9NVI7 | ATAD3A | ATPase family AAA domain-containing protein 3A | 71.3 |  | 1.7E+07 | 5.5E+00 |  |
| Q53GS9 | USP39 | U4/U6.U5 tri-snRNP-associated protein 2 | 65.3 |  | 2.1E+07 | 2.6E+00 |  |
| P28799 | GRN | Granulins | 63.5 |  | 2.3E+02 | 1.1E+06 |  |
| P35637 | FUS | RNA-binding protein FUS | 53.4 |  | 4.6E+07 | 2.1E+06 | Methyl [R422] |
| P07437 | TUBB | Tubulin beta chain | 49.6 |  | 6.7E+07 | 8.6E+06 | Methyl [R2] |
| Q07666 | KHDRBS1 | KH domain-containing, RNA-binding, signal transduction-associated protein 1 | 48.2 |  | 1.2E+07 | 5.0E+05 |  |
| P29508 | SERPINB3 | Serpin B3 | 44.5 |  | 1.5E+07 | 3.2E+06 |  |
| Q12905 | ILF2 | Interleukin enhancer-binding factor 2 | 43.0 |  | 4.0E+07 | 1.1E+07 |  |
| P68133 | ACTA1 | Actin, alpha skeletal muscle | 42.0 |  | 2.5E+09 | 2.4E+00 |  |
| P68032 | ACTC1 | Actin, alpha cardiac muscle 1 | 42.0 |  | 8.1E+05 | 1.6E+07 |  |
| Q562R1 | ACTBL2 | Beta-actin-like protein 2 | 42.0 |  | 1.7E+01 | 8.8E+00 |  |
| P08754 | GNAI3 | Guanine nucleotide-binding protein G(k) subunit alpha | 40.5 |  | 1.1E+08 | 4.9E+00 |  |
| Q9NYL9 | TMOD3 | Tropomodulin-3 | 39.6 |  | 3.6E+07 | 6.6E+06 |  |
| P62873 | GNB1 | Guanine nucleotide-binding protein G(I)/G(S)/G(T) subunit beta-1 | 37.4 |  | 1.4E+08 | 2.6E+00 |  |
| P01857 | IGHG1 | Ig gamma-1 chain C region | 36.1 |  | 5.5E+00 | 2.6E+00 |  |
| P67809 | YBX1 | Nuclease-sensitive element-binding protein 1 | 35.9 |  | 4.1E+07 | 7.8E+06 | Methyl [R239] |
| P05089 | ARG1 | Arginase-1 | 34.7 |  | 3.0E+00 | 1.4E+01 |  |
| P06753 | TPM3 | Tropomyosin alpha-3 chain | 32.9 |  | 6.2E+00 | 2.4E+01 | Methyl [R179] |
| P52907 | CAPZA1 | F-actin-capping protein subunit alpha-1 | 32.9 |  | 3.7E+01 | 2.9E+06 |  |
| Q16637 | SMN1 | Survival motor neuron protein | 31.8 |  | 4.0E+00 | 4.4E+00 |  |
| P67936 | TPM4 | Tropomyosin alpha-4 chain | 28.5 |  | 7.1E+00 | 6.9E+06 |  |
| P09661 | SNRPA1 | U2 small nuclear ribonucleoprotein A' | 28.4 |  | 2.9E+00 | 5.6E+06 |  |
| P09211 | GSTP1 | Glutathione S-transferase P | 23.3 |  | 4.7E+00 | 4.0E+00 | Methyl [R101] |
| O60814 | HIST1H2BK | Histone H2B type 1-K | 13.9 |  | 3.7E+07 | 2.4E+00 |  |
| P20930 | FLG | Filaggrin | 434.9 | 3.4E+00 |  |  |  |
| O95613 | PCNT | Pericentrin | 377.8 | 3.2E+00 |  |  |  |
| P15924 | DSP | Desmoplakin | 331.6 | 5.4E+00 |  |  | Methyl [R734] |
| Q86YZ3 | HRNR | Hornerin | 282.2 | 3.1E+00 |  |  |  |
| Q6P2Q9 | PRPF8 | Pre-mRNA-processing-splicing factor 8 | 273.4 | 3.5E+00 |  |  |  |
| P35580 | MYH10 | Myosin-10 | 228.9 | 1.2E+01 |  |  | Methyl [R108; R163; R1198] |
| P35749 | MYH11 | Myosin-11 | 227.2 | 3.7E+01 |  |  | Methyl [R1471] |
| P0C0L4 | C4A | Complement C4-A | 192.7 | 1.2E+01 |  |  |  |
| P01024 | C3 | Complement C3 | 187.0 | 3.9E+00 |  |  |  |
| Q08378 | GOLGA3 | Golgin subfamily A member 3 | 167.3 | 1.1E+06 |  |  |  |
| Q92626 | PXDN | Peroxidasin homolog | 165.2 | 4.6E+00 |  |  |  |
| O75533 | SF3B1 | Splicing factor 3B subunit 1 | 145.7 | 6.2E+06 |  |  |  |
| Q69YQ0 | SPECC1L | Cytospin-A | 124.5 | 3.4E+06 |  |  |  |
| O43896 | KIF1C | Kinesin-like protein KIF1C | 122.9 | 2.4E+00 |  |  |  |
| P57678 | GEMIN4 | Gem-associated protein 4 | 120.0 | 3.7E+06 |  |  |  |
| Q02413 | DSG1 | Desmoglein-1 | 113.7 | 3.1E+00 |  |  | Methyl [R219; R227] |
| Q15029 | EFTUD2 | 116 kDa U5 small nuclear ribonucleoprotein component | 109.4 | 6.7E+00 |  |  |  |
| Q9Y2W1 | THRAP3 | Thyroid hormone receptor-associated protein 3 | 108.6 | 3.0E+00 |  |  | Methyl [R188; R572] |
| O94906 | PRPF6 | Pre-mRNA-processing factor 6 | 106.9 | 3.5E+00 |  |  | Methyl [R23; R31; R254] |
| Q9NYF8 | BCLAF1 | Bcl-2-associated transcription factor 1 | 106.1 | 3.1E+00 |  |  |  |
| Q15424 | SAFB | Scaffold attachment factor B1 | 102.6 | 5.2E+00 |  |  |  |
| Q9BXP5 | SRRT | Serrate RNA effector molecule homolog | 100.6 | 2.1E+00 |  |  |  |
| Q13435 | SF3B2 | Splicing factor 3B subunit 2 | 100.2 | 2.2E+00 |  |  |  |
| Q08554 | DSC1 | Desmocollin-1 | 99.9 | 5.6E+00 |  |  |  |
| Q14574 | DSC3 | Desmocollin-3 | 99.9 | 5.5E+00 |  |  |  |
| Q14974 | KPNB1 | Importin subunit beta-1 | 97.1 | 7.7E+00 |  |  |  |
| Q9BUQ8 | DDX23 | Probable ATP-dependent RNA helicase DDX23 | 95.5 | 3.5E+00 |  |  |  |
| P13639 | EEF2 | Elongation factor 2 | 95.3 | 8.8E+05 |  |  |  |
| Q99459 | CDC5L | Cell division cycle 5-like protein | 92.2 | 3.2E+00 |  |  |  |
| Q9UHI6 | DDX20 | Probable ATP-dependent RNA helicase DDX20 | 92.2 | 5.4E+06 |  |  |  |
| O43290 | SART1 | U4/U6.U5 tri-snRNP-associated protein 1 | 90.2 | 2.0E+00 |  |  |  |
| Q15459 | SF3A1 | Splicing factor 3A subunit 1 | 88.8 | 2.4E+00 |  |  |  |
| Q86TB9 | PATL1 | Protein PAT1 homolog 1 | 86.8 | 1.9E+06 |  |  |  |
| P08238 | HSP90AB1 | Heat shock protein HSP 90-beta | 83.2 | 6.2E+05 |  |  |  |
| Q13835 | PKP1 | Plakophilin-1 | 82.8 | 4.4E+00 |  |  |  |
| P14923 | JUP | Junction plakoglobin | 81.7 | 4.8E+00 |  |  | Methyl [R477] |
| Q08188 | TGM3 | Protein-glutamine gamma-glutamyltransferase E | 76.6 | 3.8E+00 |  |  |  |
| P11142 | HSPA8 | Heat shock cognate 71 kDa protein | 70.9 | 4.2E+00 |  |  |  |
| Q8WVV4 | POF1B | Protein POF1B | 68.0 | 1.5E+01 |  |  |  |
| Q8TF66 | LRRC15 | Leucine-rich repeat-containing protein 15 | 64.3 | 5.1E+07 |  |  |  |
| Q9UI42 | CPA4 | Carboxypeptidase A4 | 47.3 | 2.4E+07 |  |  |  |
| Q5VU13 | VSIG8 | V-set and immunoglobulin domain-containing protein 8 | 43.9 | 2.0E+07 |  |  |  |
| P63104 | YWHAZ | 14-3-3 protein zeta/delta | 27.7 | 6.8E+04 |  |  |  |
| P68871 | HBB | Hemoglobin subunit beta | 16.0 | 2.2E+01 |  |  |  |
| P21333 | FLNA | Filamin-A | 280.6 |  | 3.1E+07 |  |  |
| Q01082 | SPTBN1 | Spectrin beta chain, non-erythrocytic 1 | 274.4 |  | 3.8E+07 |  |  |
| Q92614 | MYO18A | Unconventional myosin-XVIIIa | 233.0 |  | 1.1E+08 |  |  |
| Q9Y4I1 | MYO5A | Unconventional myosin-Va | 215.3 |  | 2.9E+07 |  |  |
| Q00610 | CLTC | Clathrin heavy chain 1 | 191.5 |  | 1.7E+07 |  |  |
| Q9BZF9 | UACA | Uveal autoantigen with coiled-coil domains and ankyrin repeats | 162.4 |  | 5.6E+06 |  |  |
| Q9UM54 | MYO6 | Unconventional myosin-VI | 149.6 |  | 1.4E+07 |  |  |
| Q13045 | FLII | Protein flightless-1 homolog | 144.7 |  | 1.6E+07 |  |  |
| Q08211 | DHX9 | ATP-dependent RNA helicase A | 140.9 |  | 1.4E+07 |  |  |
| Q9P2M7 | CGN | Cingulin | 136.3 |  | 4.5E+07 |  |  |
| Q7L2E3 | DHX30 | Putative ATP-dependent RNA helicase DHX30 | 133.9 |  | 2.6E+07 |  |  |
| Q92900 | UPF1 | Regulator of nonsense transcripts 1 | 124.3 |  | 2.7E+07 |  |  |
| O00159 | MYO1C | Unconventional myosin-Ic | 121.6 |  | 4.7E+00 |  |  |
| Q6WCQ1 | MPRIP | Myosin phosphatase Rho-interacting protein | 116.5 |  | 2.1E+06 |  |  |
| O14974 | PPP1R12A | Protein phosphatase 1 regulatory subunit 12A | 115.2 |  | 1.1E+07 |  |  |
| Q8WWM7 | ATXN2L | Ataxin-2-like protein | 113.3 |  | 4.1E+07 |  |  |
| P12036 | NEFH | Neurofilament heavy polypeptide | 112.4 |  | 2.8E+07 |  |  |
| Q02241 | KIF23 | Kinesin-like protein KIF23 | 110.0 |  | 8.9E+06 |  |  |
| O95782 | AP2A1 | AP-2 complex subunit alpha-1 | 107.5 |  | 1.2E+07 |  |  |
| P12814 | ACTN1 | Alpha-actinin-1 | 103.0 |  | 2.6E+07 |  |  |
| P07197 | NEFM | Neurofilament medium polypeptide | 102.4 |  | 6.4E+07 |  |  |
| Q96SB3 | PPP1R9B | Neurabin-2 | 89.1 |  | 5.2E+07 |  |  |
| Q14694 | USP10 | Ubiquitin carboxyl-terminal hydrolase 10 | 87.1 |  | 1.4E+07 |  |  |
| Q9UHB6 | LIMA1 | LIM domain and actin-binding protein 1 | 85.2 |  | 4.5E+00 |  |  |
| Q14444 | CAPRIN1 | Caprin-1 | 78.3 |  | 5.2E+07 |  | Methyl [R633; R640] |
| P51116 | FXR2 | Fragile X mental retardation syndrome-related protein 2 | 74.2 |  | 1.9E+07 |  |  |
| Q5T9A4 | ATAD3B | ATPase family AAA domain-containing protein 3B | 72.5 |  | 2.7E+07 |  |  |
| Q8NBJ5 | COLGALT1 | Procollagen galactosyltransferase 1 | 71.6 |  | 2.5E+07 |  |  |
| Q16643 | DBN1 | Drebrin | 71.4 |  | 1.8E+01 |  |  |
| Q06787 | FMR1 | Synaptic functional regulator FMR1 | 71.1 |  | 1.5E+07 |  |  |
| Q9H0H5 | RACGAP1 | Rac GTPase-activating protein 1 | 71.0 |  | 1.7E+07 |  |  |
| P11940 | PABPC1 | Polyadenylate-binding protein 1 | 70.6 |  | 2.4E+08 |  |  |
| Q9UJV9 | DDX41 | Probable ATP-dependent RNA helicase DDX41 | 69.8 |  | 1.6E+07 |  |  |
| O60506 | SYNCRIP | Heterogeneous nuclear ribonucleoprotein Q | 69.6 |  | 2.1E+07 |  |  |
| P17844 | DDX5 | Probable ATP-dependent RNA helicase DDX5 | 69.1 |  | 5.6E+07 |  |  |
| Q6NYC8 | PPP1R18 | Phostensin | 67.9 |  | 1.4E+07 |  |  |
| O00425 | IGF2BP3 | Insulin-like growth factor 2 mRNA-binding protein 3 | 63.7 |  | 3.0E+07 |  |  |
| Q9NZI8 | IGF2BP1 | Insulin-like growth factor 2 mRNA-binding protein 1 | 63.4 |  | 2.3E+07 |  |  |
| P07196 | NEFL | Neurofilament light polypeptide | 61.5 |  | 2.1E+08 |  |  |
| P10809 | HSPD1 | 60 kDa heat shock protein, mitochondrial | 61.0 |  | 1.1E+07 |  |  |
| P07948 | LYN | Tyrosine-protein kinase Lyn | 58.5 |  | 5.1E+06 |  |  |
| Q9NP81 | SARS2 | Serine--tRNA ligase, mitochondrial | 58.2 |  | 3.9E+07 |  |  |
| P26599 | PTBP1 | Polypyrimidine tract-binding protein 1 | 57.2 |  | 3.0E+07 |  |  |
| P06576 | ATP5B | ATP synthase subunit beta, mitochondrial | 56.5 |  | 2.0E+07 |  |  |
| Q8NCA5 | FAM98A | Protein FAM98A | 55.4 |  | 1.4E+07 |  |  |
| Q9Y3I0 | RTCB | tRNA-splicing ligase RtcB homolog | 55.2 |  | 2.9E+07 |  |  |
| Q16658 | FSCN1 | Fascin | 54.5 |  | 3.4E+07 |  |  |
| Q9UN86 | G3BP2 | Ras GTPase-activating protein-binding protein 2 | 54.1 |  | 6.5E+07 |  |  |
| Q9ULV4 | CORO1C | Coronin-1C | 53.2 |  | 5.0E+07 |  |  |
| Q13283 | G3BP1 | Ras GTPase-activating protein-binding protein 1 | 52.1 |  | 8.0E+07 |  | Methyl [R429; R435] |
| P55084 | HADHB | Trifunctional enzyme subunit beta, mitochondrial | 51.3 |  | 1.5E+07 |  |  |
| P61978 | HNRNPK | Heterogeneous nuclear ribonucleoprotein K | 50.9 |  | 2.1E+07 |  |  |
| Q9BTD8 | RBM42 | RNA-binding protein 42 | 50.4 |  | 1.5E+06 |  |  |
| P68363 | TUBA1B | Tubulin alpha-1B chain | 50.1 |  | 5.4E+07 |  |  |
| P68371 | TUBB4B | Tubulin beta-4B chain | 49.8 |  | 2.0E+07 |  | Methyl [R2] |
| P49411 | TUFM | Elongation factor Tu, mitochondrial | 49.5 |  | 1.4E+07 |  |  |
| P61158 | ACTR3 | Actin-related protein 3 | 47.3 |  | 4.0E+07 |  |  |
| O75955 | FLOT1 | Flotillin-1 | 47.3 |  | 4.6E+07 |  |  |
| P48730 | CSNK1D | Casein kinase I isoform delta | 47.3 |  | 1.2E+07 |  |  |
| Q8IW75 | SERPINA12 | Serpin A12 | 47.1 |  | 9.6E+06 |  |  |
| Q14254 | FLOT2 | Flotillin-2 | 47.0 |  | 2.6E+07 |  |  |
| P38919 | EIF4A3 | Eukaryotic initiation factor 4A-III | 46.8 |  | 2.2E+07 |  |  |
| P52597 | HNRNPF | Heterogeneous nuclear ribonucleoprotein F | 45.6 |  | 2.0E+07 |  |  |
| P68400 | CSNK2A1 | Casein kinase II subunit alpha | 45.1 |  | 7.1E+06 |  |  |
| P61160 | ACTR2 | Actin-related protein 2 | 44.7 |  | 4.6E+07 |  |  |
| P07339 | CTSD | Cathepsin D | 44.5 |  | 2.9E+00 |  |  |
| O76081 | RGS20 | Regulator of G-protein signaling 20 | 43.7 |  | 1.1E+07 |  |  |
| P17302 | GJA1 | Gap junction alpha-1 protein | 43.0 |  | 1.7E+07 |  |  |
| P29992 | GNA11 | Guanine nucleotide-binding protein subunit alpha-11 | 42.1 |  | 1.0E+07 |  |  |
| P04899 | GNAI2 | Guanine nucleotide-binding protein G(i) subunit alpha-2 | 40.4 |  | 1.4E+08 |  |  |
| P63096 | GNAI1 | Guanine nucleotide-binding protein G(i) subunit alpha-1 | 40.3 |  | 1.1E+08 |  | Methyl [R161] |
| Q12792 | TWF1 | Twinfilin-1 | 40.3 |  | 6.9E+06 |  |  |
| P09471 | GNAO1 | Guanine nucleotide-binding protein G(o) subunit alpha | 40.0 |  | 1.3E+07 |  |  |
| Q8ND76 | CCNY | Cyclin-Y | 39.3 |  | 8.7E+06 |  |  |
| P09651 | HNRNPA1 | Heterogeneous nuclear ribonucleoprotein A1 | 38.7 |  | 3.1E+01 |  | Methyl [R194; R196] |
| P62136 | PPP1CA | Serine/threonine-protein phosphatase PP1-alpha catalytic subunit | 37.5 |  | 4.8E+07 |  |  |
| P62879 | GNB2 | Guanine nucleotide-binding protein G(I)/G(S)/G(T) subunit beta-2 | 37.3 |  | 8.0E+07 |  |  |
| P31942 | HNRNPH3 | Heterogeneous nuclear ribonucleoprotein H3 | 36.9 |  | 3.2E+07 |  |  |
| Q99729 | HNRNPAB | Heterogeneous nuclear ribonucleoprotein A/B | 36.2 |  | 1.4E+07 |  |  |
| Q15717 | ELAVL1 | ELAV-like protein 1 | 36.1 |  | 4.8E+07 |  |  |
| O15144 | ARPC2 | Actin-related protein 2/3 complex subunit 2 | 34.3 |  | 1.0E+01 |  |  |
| P22087 | FBL | rRNA 2'-O-methyltransferase fibrillarin | 33.8 |  | 1.7E+07 |  |  |
| P07910 | HNRNPC | Heterogeneous nuclear ribonucleoproteins C1/C2 | 33.7 |  | 9.1E+00 |  |  |
| P36542 | ATP5C1 | ATP synthase subunit gamma, mitochondrial | 33.0 |  | 8.7E+06 |  |  |
| P47755 | CAPZA2 | F-actin-capping protein subunit alpha-2 | 32.9 |  | 4.3E+07 |  |  |
| P07951 | TPM2 | Tropomyosin beta chain | 32.8 |  | 8.1E+00 |  |  |
| P09493 | TPM1 | Tropomyosin alpha-1 chain | 32.7 |  | 3.2E+00 |  | Methyl [R160] |
| Q9UKM9 | RALY | RNA-binding protein Raly | 32.4 |  | 8.4E+06 |  |  |
| Q96HS1 | PGAM5 | Serine/threonine-protein phosphatase PGAM5, mitochondrial | 32.0 |  | 5.0E+00 |  |  |
| P27105 | STOM | Erythrocyte band 7 integral membrane protein | 31.7 |  | 5.7E+06 |  |  |
| P47756 | CAPZB | F-actin-capping protein subunit beta | 31.3 |  | 1.2E+01 |  |  |
| Q13151 | HNRNPA0 | Heterogeneous nuclear ribonucleoprotein A0 | 30.8 |  | 2.5E+01 |  |  |
| P09038 | FGF2 | Fibroblast growth factor 2 | 30.8 |  | 1.0E+07 |  |  |
| P15927 | RPA2 | Replication protein A 32 kDa subunit | 29.2 |  | 2.4E+00 |  |  |
| P50402 | EMD | Emerin | 29.0 |  | 2.1E+07 |  |  |
| Q07955 | SRSF1 | Serine/arginine-rich splicing factor 1 | 27.7 |  | 8.2E+06 |  |  |
| P31944 | CASP14 | Caspase-14 | 27.7 |  | 3.2E+00 |  |  |
| P49862 | KLK7 | Kallikrein-7 | 27.5 |  | 5.5E+00 |  |  |
| Q16629 | SRSF7 | Serine/arginine-rich splicing factor 7 | 27.4 |  | 4.0E+07 |  |  |
| P09496 | CLTA | Clathrin light chain A | 27.1 |  | 1.6E+07 |  |  |
| Q9BUP0 | EFHD1 | EF-hand domain-containing protein D1 | 26.9 |  | 3.0E+07 |  |  |
| Q86V81 | ALYREF | THO complex subunit 4 | 26.9 |  | 1.5E+07 |  | Methyl [R58; R63] |
| Q96C19 | EFHD2 | EF-hand domain-containing protein D2 | 26.7 |  | 2.6E+08 |  |  |
| Q13242 | SRSF9 | Serine/arginine-rich splicing factor 9 | 25.5 |  | 5.1E+06 |  |  |
| P08579 | SNRPB2 | U2 small nuclear ribonucleoprotein B'' | 25.5 |  | 3.7E+00 |  |  |
| Q9H444 | CHMP4B | Charged multivesicular body protein 4b | 24.9 |  | 1.9E+07 |  |  |
| O00161 | SNAP23 | Synaptosomal-associated protein 23 | 23.3 |  | 4.1E+07 |  | Methyl [R148] |
| P48047 | ATP5O | ATP synthase subunit O, mitochondrial | 23.3 |  | 1.3E+07 |  |  |
| B9A064 | IGLL5 | Immunoglobulin lambda-like polypeptide 5 | 23.0 |  | 9.7E+06 |  |  |
| P04792 | HSPB1 | Heat shock protein beta-1 | 22.8 |  | 6.5E+00 |  |  |
| P14649 | MYL6B | Myosin light chain 6B | 22.8 |  | 2.7E+00 |  |  |
| Q06830 | PRDX1 | Peroxiredoxin-1 | 22.1 |  | 5.5E+00 |  |  |
| P32119 | PRDX2 | Peroxiredoxin-2 | 21.9 |  | 3.6E+00 |  |  |
| P30086 | PEBP1 | Phosphatidylethanolamine-binding protein 1 | 21.0 |  | 3.1E+00 |  |  |
| O75223 | GGCT | Gamma-glutamylcyclotransferase | 21.0 |  | 1.8E+07 |  |  |
| O15145 | ARPC3 | Actin-related protein 2/3 complex subunit 3 | 20.5 |  | 9.3E+00 |  |  |
| P24844 | MYL9 | Myosin regulatory light polypeptide 9 | 19.8 |  | 1.2E+01 |  | Methyl [R45] |
| P59998 | ARPC4 | Actin-related protein 2/3 complex subunit 4 | 19.7 |  | 7.8E+00 |  |  |
| P84103 | SRSF3 | Serine/arginine-rich splicing factor 3 | 19.3 |  | 1.5E+01 |  |  |
| P31025 | LCN1 | Lipocalin-1 | 19.2 |  | 4.3E+00 |  |  |
| O43447 | PPIH | Peptidyl-prolyl cis-trans isomerase H | 19.2 |  | 2.5E+00 |  |  |
| P23528 | CFL1 | Cofilin-1 | 18.5 |  | 2.5E+07 |  |  |
| Q6IAA8 | LAMTOR1 | Ragulator complex protein LAMTOR1 | 17.7 |  | 2.0E+07 |  |  |
| Q9BPX5 | ARPC5L | Actin-related protein 2/3 complex subunit 5-like protein | 16.9 |  | 1.6E+07 |  |  |
| P60660 | MYL6 | Myosin light polypeptide 6 | 16.9 |  | 7.3E+00 |  |  |
| P62158 | CALM1 | Calmodulin | 16.8 |  | 7.8E+00 |  |  |
| Q71DI3 | HIST2H3A | Histone H3.2 | 15.4 |  | 7.1E+07 |  |  |
| Q01469 | FABP5 | Fatty acid-binding protein, epidermal | 15.2 |  | 2.3E+07 |  |  |
| P13987 | CD59 | CD59 glycoprotein | 14.2 |  | 1.4E+07 |  |  |
| P04908 | HIST1H2AB | Histone H2A type 1-B/E | 14.1 |  | 4.7E+00 |  |  |
| P62314 | SNRPD1 | Small nuclear ribonucleoprotein Sm D1 | 13.3 |  | 1.1E+07 |  |  |
| P31151 | S100A7 | Protein S100-A7 | 11.5 |  | 6.3E+00 |  |  |
| P01040 | CSTA | Cystatin-A | 11.0 |  | 3.8E+00 |  |  |
| P05109 | S100A8 | Protein S100-A8 | 10.8 |  | 3.4E+06 |  |  |
| Q9C0J8 | WDR33 | pre-mRNA 3' end processing protein WDR33 | 145.8 |  |  | 4.0E+06 | Methyl [R1305; R1308] |
| A5A3E0 | POTEF | POTE ankyrin domain family member F | 121.4 |  |  | 1.1E+07 |  |
| Q96H55 | MYO19 | Unconventional myosin-XIX | 109.1 |  |  | 3.4E+00 |  |
| O95782 | AP2A1 | AP-2 complex subunit alpha-1 | 107.5 |  |  | 6.6E+00 | Methyl [R832; R843; R845] |
| P53814 | SMTN | Smoothelin | 99.0 |  |  | 7.2E+06 |  |
| Q9P2I0 | CPSF2 | Cleavage and polyadenylation specificity factor subunit 2 | 88.4 |  |  | 8.0E+06 |  |
| P02788 | LTF | Lactotransferrin | 78.1 |  |  | 5.2E+06 |  |
| O43395 | PRPF3 | U4/U6 small nuclear ribonucleoprotein Prp3 | 77.5 |  |  | 1.4E+06 |  |
| P52272 | HNRNPM | Heterogeneous nuclear ribonucleoprotein M | 77.5 |  |  | 1.2E+06 |  |
| P08107 | HSPA1A | Heat shock 70 kDa protein 1A/1B | 70.0 |  |  | 9.1E+05 |  |
| P00738 | HP | Haptoglobin | 45.2 |  |  | 5.1E+00 |  |
| Q7L273 | KCTD9 | BTB/POZ domain-containing protein KCTD9 | 42.5 |  |  | 1.1E+06 |  |
| P01860 | IGHG3 | Ig gamma-3 chain C region | 41.3 |  |  | 8.0E+00 |  |
| O14893 | GEMIN2 | Gem-associated protein 2 | 31.6 |  |  | 3.7E+06 |  |
| Q6FI13 | HIST2H2AA3 | Histone H2A type 2-A | 14.1 |  |  | 2.7E+00 |  |
| P62805 | HIST1H4A | Histone H4 | 11.4 |  |  | 3.7E+00 |  |

^a^MTAP/Mock; ^b^sgCTRL/sgMTAP

**Appendix Table S6.** Clinicopathologic characteristics of lung adenocarcinoma cohort from Taiwan Cancer Moonshot

| **Characteristic** | **Total patients** |
| --- | --- |
| **Patient No.** | n=89 |
| **Age** |  |
| ＜60 years | 30 (33.71%) |
| ≧60 years | 59 (66.29%) |
| **Gender** |  |
| Male | 35 (39.33%) |
| Female | 54 (60.67%) |
| **Smoking status** |  |
| Non-smoker | 77 (86.52%) |
| Ex-smoker | 9 (10.11%) |
| Current smoker | 3 (3.37%) |
| **Pathology differentiation*** |  |
| Well | 7 (8.05%) |
| Moderate | 56 (64.37%) |
| Poor | 24 (27.59%) |
| **Stage** |  |
| I | 71 (79.78%) |
| II | 6 (6.74%) |
| III-IV | 12 (13.48%) |
| **EGFR status** |  |
| Wild-type | 13 (14.61%) |
| L858R | 35 (39.33%) |
| Exon 19 deletion | 31 (34.83%) |
| Others | 10 (11.24%) |
| **TP53 status** |  |
| Wild-type | 63 (70.79%) |
| Mutations | 26 (29.21%) |

*Two patients without pathology differentiation information

**Appendix Table S7.** Clinicopathologic characteristics of 124 lung cancer patients in relation to MTAP expression

| **Characteristic** | **Total patients** | **High MTAP**  Patient No. (%) | **Low MTAP**  Patient No. (%) | ***P* value** |
| --- | --- | --- | --- | --- |
| **Patient No.** | n=124 | n=44 | n=80 |  |
| **Age (meanSD)**  | 66.5±10.7 | 66.3±10.2 | 66.6±11.1 | 0.876^†^ |
| **Gender** |  |  |  | 0.845^‡^ |
| Male | 55 | 19 (43.2%) | 36 (45.0%) |  |
| Female | 69 | 25 (56.8%) | 44 (55.0%) |  |
| **Race** |  |  |  | 0.786^‡^ |
| White | 73 | 24 (54.5%) | 49 (61.3%) |  |
| Black | 4 | 1 (2.3%) | 3 (3.8%) |  |
| Asian | 6 | 2 (4.5%) | 4 (5.0%) |  |
| Others | 41 | 17 (38.6%) | 24 (30.0%) |  |
| **Grade*** |  |  |  | 0.234^‡^ |
| Well | 16 | 3 (9.1%) | 13 (21.0%) |  |
| Moderate | 40 | 17 (51.5%) | 23 (37.1%) |  |
| Poor | 39 | 13 (39.4%) | 26 (41.9%) |  |
| **Histological subtype** |  |  |  | 0.211^‡^ |
| Adenocarcinoma | 57 | 17 (38.6%) | 40 (50.0%) |  |
| Squamous cell carcinoma | 38 | 16 (36.4%) | 22 (27.5%) |  |
| Large cell carcinoma | 8 | 5 (11.4%) | 3 (3.8%) |  |
| Others | 21 | 6 (13.6%) | 15 (18.8%) |  |

^†^Student *t* test

^‡^Chi-square test

*Some patients without grade information

**Appendix Table S8.** Clinicopathologic characteristics of 124 lung cancer patients in relation to vimentin expression

| **Characteristic** | **Total patients** | **High vimentin**  Patient No. (%) | **Low vimentin**  Patient No. (%) | ***P* value** |
| --- | --- | --- | --- | --- |
| **Patient No.** | n=124 | n=56 | n=68 |  |
| **Age (meanSD)**  | 66.5±10.7 | 67.3±11.0 | 65.8±10.4 | 0.434^†^ |
| **Gender** |  |  |  | 0.251^‡^ |
| Male | 55 | 28 (50.0%) | 27 (39.7%) |  |
| Female | 69 | 28 (50.0%) | 41 (60.3%) |  |
| **Race** |  |  |  | 0.209^‡^ |
| White | 73 | 36 (64.3%) | 37 (54.4%) |  |
| Black | 4 | 3 (5.4%) | 1 (1.5%) |  |
| Asian | 6 | 1 (1.8%) | 5 (7.4%) |  |
| Others | 41 | 16 (28.6%) | 25 (36.8%) |  |
| **Grade*** |  |  |  | 0.947^‡^ |
| Well | 16 | 7 (16.7%) | 9 (17.0%) |  |
| Moderate | 40 | 17 (40.5%) | 23 (43.4%) |  |
| Poor | 39 | 18 (42.9%) | 21 (39.6%) |  |
| **Histological subtype** |  |  |  | 0.298^‡^ |
| Adenocarcinoma | 57 | 27 (48.2%) | 30 (44.1%) |  |
| Squamous cell carcinoma | 38 | 18 (32.1%) | 20 (29.4%) |  |
| Large cell carcinoma | 8 | 1 (1.8%) | 7 (10.3%) |  |
| Others | 21 | 10 (17.9%) | 11 (16.2%) |  |

^†^Student *t* test

^‡^Chi-square test

*Some patients without grade information
