# Supplementary material for: Reduced symmetric dimethylation stabilizes vimentin and promotes metastasis in MTAP‐deficient lung cancer
Source: EMBO Rep. 2022 Jun 29;23(8):e54265. doi: 10.15252/embr.202154265 (PMC9346486; doi:10.15252/embr.202154265)
Supplement: Supplementary file 2 — Expanded View Figures PDF [file EMBR-23-e54265-s003.pdf]

Expanded View Figures

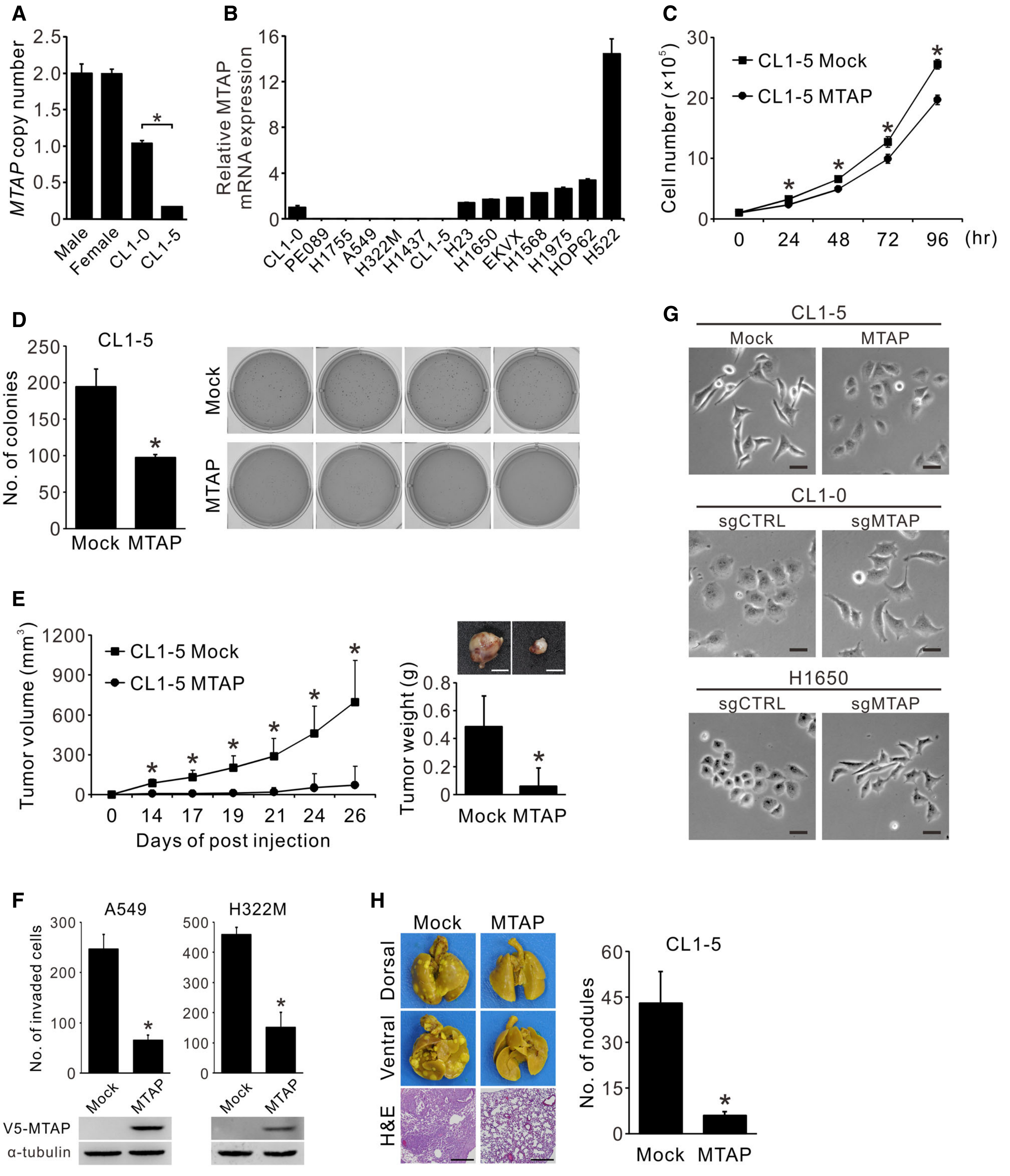

Figure EV1.

Figure EV1. Expression and effect of MTAP on cell proliferation, colony forming, tumorigenesis, invasion, morphology, and metastasis in lung cancer cells.

- A Comparison of *MTAP* DNA copy number between PBMCs from healthy donors, CL1-0, and CL1-5 cells detected by real-time genomic PCR (mean ± SD, Student t test, $n = 3$, biological replicates, $*P < 0.05$). Ribonuclease P (*RNaseP*) served as the internal control.
- B Relative mRNA expression of *MTAP* was detected in 14 lung cancer cell lines assayed by RT-qPCR (mean ± SD, $n = 3$, biological replicates). TATA-binding protein (*TBP*) served as the internal control.
- C Effect of MTAP on cell proliferation was determined by cell collection and counting at indicated time points (mean ± SD, Student t test, $n = 3$, technical replicates, $*P < 0.05$). Data shown are representative of three independent experiments.
- D Effect of MTAP on anchorage-independent colony formation. Left: quantification of colonies stained by crystal violet and counted under phase microscopy (mean ± SD, Student t test, $n = 4$, biological replicates, $*P < 0.05$). Right: images of anchorage-independent colony formation assays of CL1-5 Mock control and MTAP-overexpressing cells.
- E CL1-5 Mock and MTAP-overexpressing transfectants were subcutaneously injected into the NOD/SCID mice to determine the tumorigenesis ability. Left: the tumor volumes measured at indicated days (mean ± SD, Student t test, $n = 8$, biological replicates, $*P < 0.05$). Right: representative images of tumors and the tumor weights measured at 27 days postinjection (mean ± SD, Student t test, $n = 8$, biological replicates, $*P < 0.05$). Scale bar, 5 mm.
- F Cell invasion abilities of MTAP-overexpressing A549 and H322M were determined by Matrigel invasion assays (mean ± SD, Student t test, $n = 3$, biological replicates, $*P < 0.05$). Bottom: the protein expression levels of V5-MTAP were detected by Western blots.
- G Effect of MTAP expression on cell morphology. Cells were examined by a phase contrast microscope. Scale bar, 50 μm. Data shown are representative of three independent experiments.
- H Micrometastatic analysis of MTAP-overexpressing cells. NOD/SCID mice were intravenously injected with CL1-5 Mock control and MTAP-overexpressing cells, and sacrificed at 10 weeks post injection. Left: the appearances and the representative hematoxylin and eosin (H&E) staining of the lung sections from mice injected with CL1-5 Mock and MTAP transfectants. Scale bar, 50 μm. Right: the gross pulmonary metastasis nodules were quantified under dissecting microscope (Mock, $n = 11$; MTAP, $n = 9$; biological replicates, mean ± SE, Student t test, $*P < 0.05$).

 ©2022 The Authors

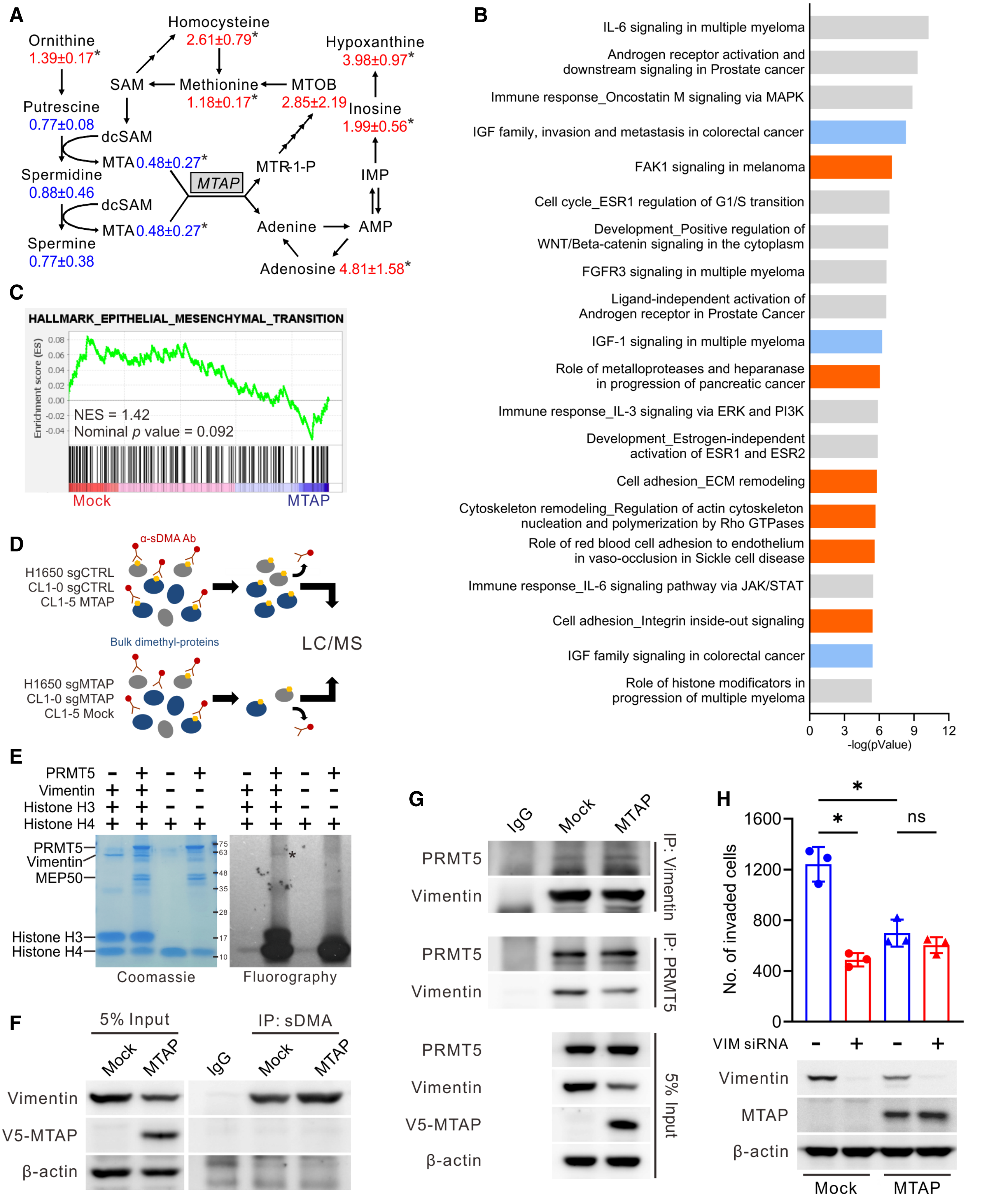
A
Ornithine 1.39±0.17*
Homocysteine 2.61±0.79*
Hypoxanthine 3.98±0.97*
SAM
Methionine 1.18±0.17*
MTOB 2.85±2.19
Putrescine 0.77±0.08
dcSAM
Inosine 1.99±0.56*
MTA 0.48±0.27*
MTR-1-P
IMP
Spermidine 0.88±0.46
MTAP
Adenine
AMP
Spermine 0.77±0.38
Adenosine 4.81±1.58*
B
IL-6 signaling in multiple myeloma
Androgen receptor activation and downstream signaling in Prostate cancer
Immune response_Oncostatin M signaling via MAPK
IGF family, invasion and metastasis in colorectal cancer
FAK1 signaling in melanoma
Cell cycle_ESR1 regulation of G1/S transition
Development_Positive regulation of WNT/Beta-catenin signaling in the cytoplasm
FGFR3 signaling in multiple myeloma
Ligand-independent activation of Androgen receptor in Prostate Cancer
IGF-1 signaling in multiple myeloma
Role of metalloproteases and heparanase in progression of pancreatic cancer
Immune response_IL-3 signaling via ERK and PI3K
Development_Estrogen-independent activation of ESR1 and ESR2
Cell adhesion_ECM remodeling
Cytoskeleton remodeling_Regulation of actin cytoskeleton nucleation and polymerization by Rho GTPases
Role of red blood cell adhesion to endothelium in vaso-occlusion in Sickle cell disease
Immune response_IL-6 signaling pathway via JAK/STAT
Cell adhesion_Integrin inside-out signaling
IGF family signaling in colorectal cancer
Role of histone modificators in progression of multiple myeloma
0 3 6 9 12
-log(pValue)
C
HALLMARK_EPITHELIAL_MESENCHYMAL_TRANSITION
Enrichment score (ES)
NES = 1.42
Nominal p value = 0.092
Mock
MTAP
D
α-sDMA Ab
H1650 sgCTRL
CL1-0 sgCTRL
CL1-5 MTAP
Bulk dimethyl-proteins
LC/MS
H1650 sgMTAP
CL1-0 sgMTAP
CL1-5 Mock
E
PRMT5
Vimentin
Histone H3
Histone H4
PRMT5
Vimentin
MEP50
Histone H3
Histone H4
Coomassie
Fluorography
F
5% Input
IP: sDMA
Mock MTAP IgG Mock MTAP
Vimentin
V5-MTAP
β-actin
G
IgG Mock MTAP
PRMT5
Vimentin
IP: Vimentin
IP: PRMT5
V5-MTAP
β-actin
5% Input
H
No. of invaded cells
ns
VIM siRNA
Vimentin
MTAP
β-actin
Mock
MTAP

Figure EV2.

Figure EV2. MTAP-mediated metabolism, signaling pathways, and methylproteome.

A MTAP-mediated metabolic alterations in polyamine, methionine, and adenine salvage pathways. The numbers are the average fold change intensities and associated errors for metabolites of CL1-5 MTAP/CL1-5 Mock (Student *t* test, $n = 3$, biological replicates, $*P < 0.05$).

B Top 20 ranking MTAP-altered pathways and cellular processes identified by the MetaCore analytical suite (version 20.4 build 70,300). Blue bars: IGF-related pathways; Orange bars: cell adhesion/cytoskeleton remodeling and invasion-related pathways.

C Gene Set Enrichment Analysis (GSEA) of expression microarray data from CL1-5 Mock and MTAP-overexpressing cells were performed using the gene set of HALLMARK_EPITHELIAL_MESENCHYMAL_TRANSITION.

D Schematic diagram of the identification of differentially symmetrically dimethylated proteins.

E *In vitro* methylation of vimentin by PRMT5. Tritiated proteins were separated by SDS-PAGE, stained with Coomassie blue (left), dried and analyzed by fluorography (right). Histone H3 and H4 proteins were used as positive controls. *: tritiated vimentin.

F Immunoprecipitation analysis for vimentin dimethylation in CL1-5 Mock and MTAP-overexpressing cells. Data shown are representative of three independent experiments.

G Immunoprecipitation analysis of the association between endogenous vimentin and endogenous PRMT5 in CL1-5 Mock and MTAP-overexpressing cells. Data shown are representative of three independent experiments.

H CL1-5 MTAP-overexpressing and Mock cells were transfected with vimentin siRNAs for 72 h and analyzed by Boyden chamber invasion assays (mean ± SD, Student *t* test, $n = 3$, biological replicates, $*P < 0.05$). The silence efficiency of vimentin was examined by Western blot.

Source data are available online for this figure.

 ©2022 The Authors

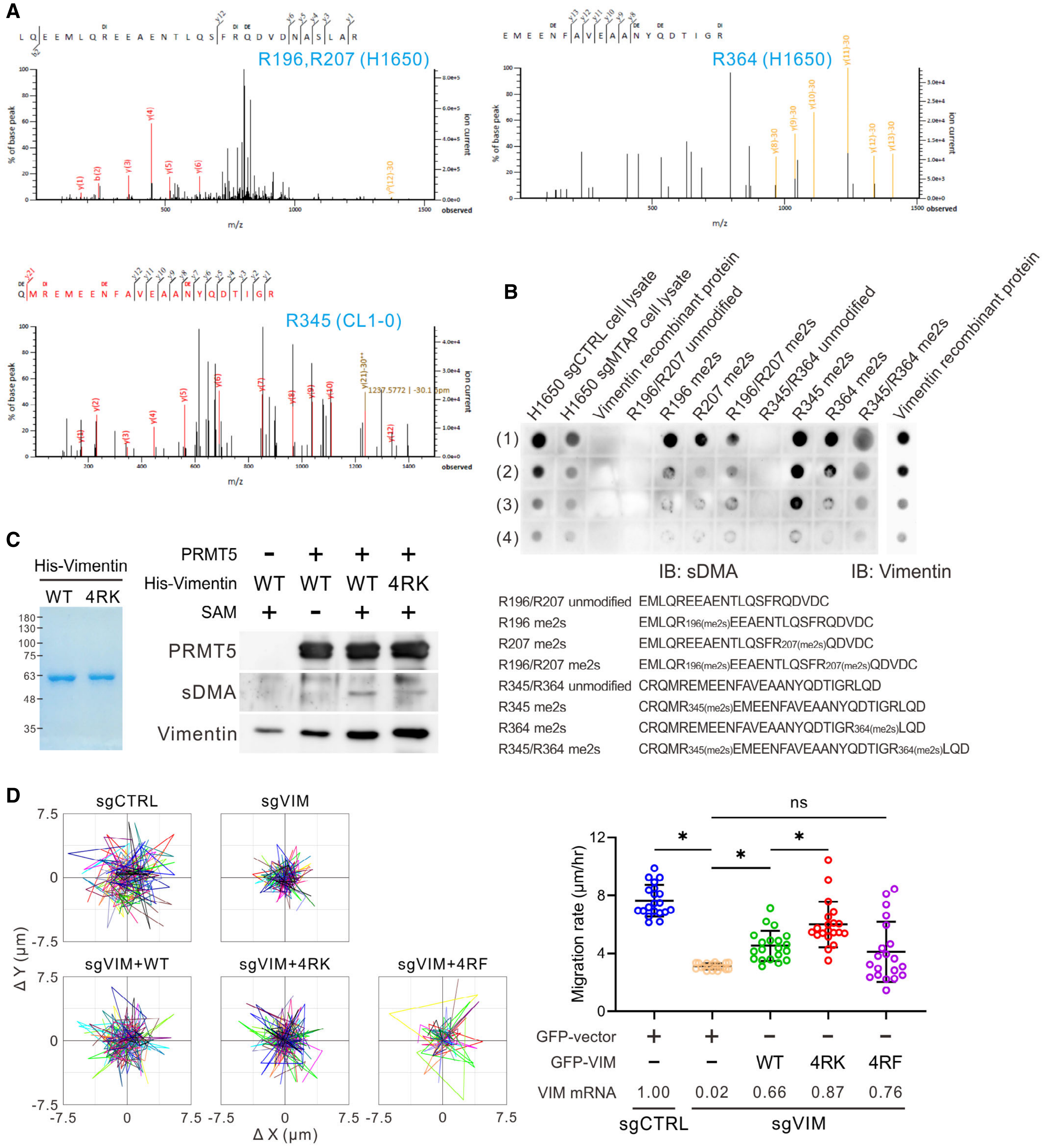
A
R196,R207 (H1650)
R364 (H1650)
R345 (CL1-0)
% of base peak
ion current
m/z
observed
B
H1650 sgCTRL cell lysate
H1650 sgMTAP cell lysate
Vimentin recombinant protein
R196/R207 unmodified
R196 me2s
R207 me2s
R196/R207 me2s
R345/R364 unmodified
R345 me2s
R364 me2s
R345/R364 me2s
Vimentin recombinant protein
(1)
(2)
(3)
(4)
IB: sDMA
IB: Vimentin
R196/R207 unmodified EMLQREEAENTLQSFRQDVDC
R196 me2s EMLQR196(me2s)EEAENTLQSFRQDVDC
R207 me2s EMLQREEAENTLQSFR207(me2s)QDVDC
R196/R207 me2s EMLQR196(me2s)EEAENTLQSFR207(me2s)QDVDC
R345/R364 unmodified CRQMREMEENFAVEAANYQDTIGRLQD
R345 me2s CRQMR345(me2s)EMEENFAVEAANYQDTIGRLQD
R364 me2s CRQMREMEENFAVEAANYQDTIGR364(me2s)LQD
R345/R364 me2s CRQMR345(me2s)EMEENFAVEAANYQDTIGR364(me2s)LQD
C
His-Vimentin
WT 4RK
180
130
100
75
63
48
35
PRMT5 - + + +
His-Vimentin WT WT WT 4RK
SAM + - + +
PRMT5
sDMA
Vimentin
D
sgCTRL
sgVIM
sgVIM+WT
sgVIM+4RK
sgVIM+4RF
Δ Y (μm)
Δ X (μm)
ns
Migration rate (μm/hr)
GFP-vector + + - - -
GFP-VIM - - WT 4RK 4RF
VIM mRNA 1.00 0.02 0.66 0.87 0.76
sgCTRL
sgVIM

Figure EV3.

◀ **Figure EV3. Effect of PRMT5-mediated sDMA on vimentin-governed cell motility.**

A MS/MS spectra evidence of sDMA at R196, R207, and R364 from H1650 vimentin and R345 from CL1-0 vimentin.

B Dot blot analysis showing specificity of sDMA antibody. H1650 cell lysates were used as positive controls. The sequences of non-methylated (unmodified) and dimethylated (me2s) vimentin peptides were shown in the bottom. H1650 cell lysates: (1) 6.5 μg, (2) 3.2 μg, (3) 1.6 μg, (4) 0.8 μg. Vimentin recombinant protein: (1) 0.5 μg, (2) 0.25 μg, (3) 0.125 μg, (4) 0.0625 μg. Vimentin peptides: (1) 5 μg, (2) 2.5 μg, (3) 1.25 μg, (4) 0.625 μg.

C *In vitro* methyltransferase assays of vimentin wild-type and 4RK mutant by PRMT5 analyzed by Western blots with anti-sDMA antibody (right). Left: purified His-vimentin proteins were stored in methylation reaction buffer, fractionated by SDS-PAGE, and visualized by Coomassie blue stain. Data shown are representative of two independent experiments.

D Single-cell tracking migration assays. CL1-5 control and vimentin-knockout cells were transfected with GFP vector or GFP-vimentin for 48 h. Cells expressing GFP fluorescence were tracked by the time-lapse video microscopy system. Representative trajectories and quantification of averaged velocity of cells (mean ± SD, Student *t* test, $n = 20$, technical replicates, $^*P < 0.05$). 4RK indicates R196/207/345/364K; 4RF indicates R196/207/345/364F. Data shown are representative of three independent experiments.

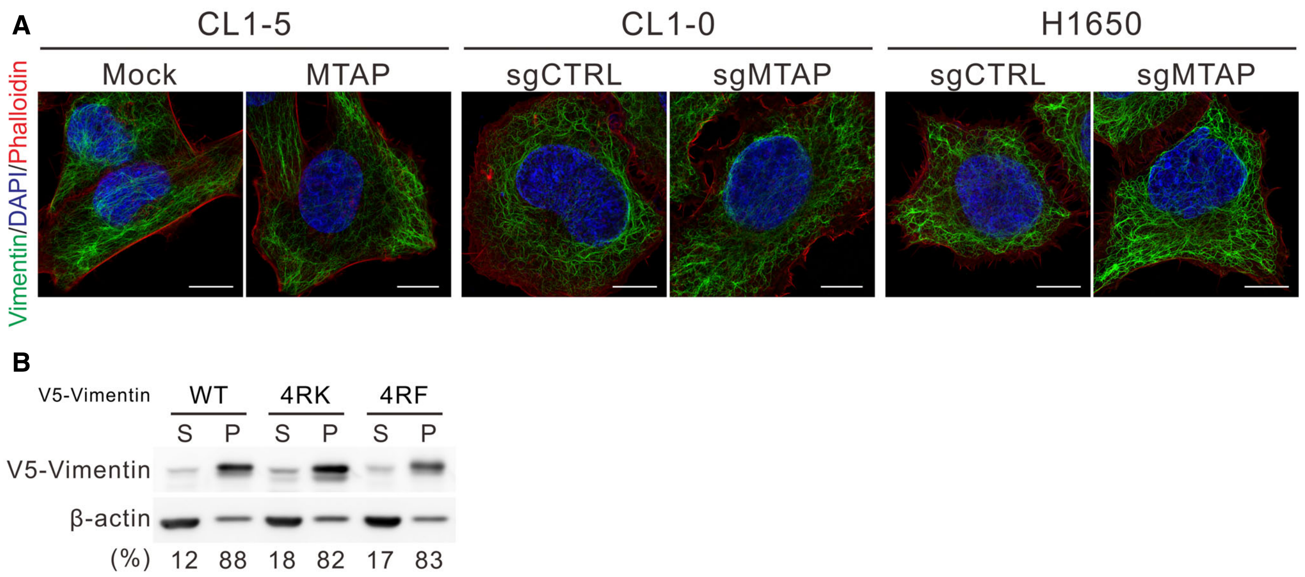

Figure EV4. Effect of MTAP-dependent sDMA on vimentin polymerization.

A Immunofluorescence staining of vimentin in indicated cell lines. F-actin was stained with phalloidin, and nucleus was stained with DAPI. Scale bar, 10 μm. Data shown are representative of three independent experiments.

B Fractionation of soluble and insoluble V5-vimentin. CL1-0 cells were transiently expressed with V5-vimentin wild-type, 4RK and 4RF mutants for 48 h. The cells were fractionated to supernatants (S) and pellets (P). After pellets were resolved by sonication, equal portions of lysates were subjected to immunoblotting. The ratio of V5-vimentin in the supernatant and pellet fractions was measured by ImageJ software. Data shown are representative of three independent experiments.

 ©2022 The Authors

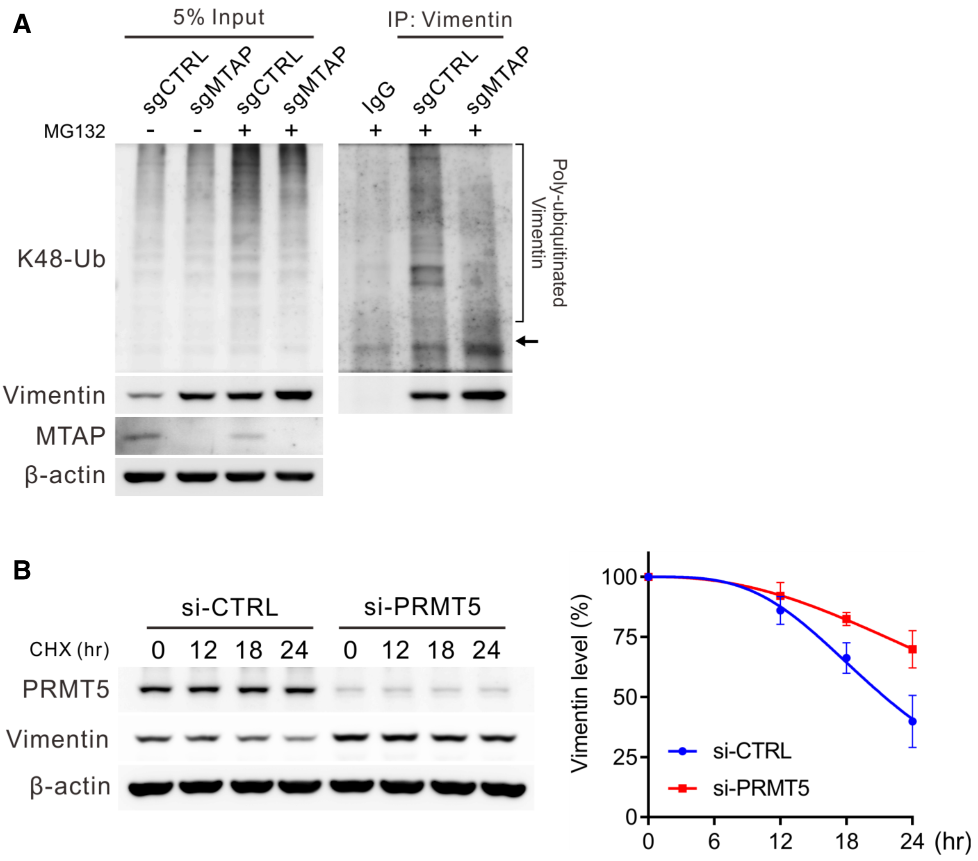

Figure EV5. Effect of sDMA on vimentin ubiquitination and protein stability.

A Immunoprecipitation analysis for K48-linked polyubiquitination of vimentin in CL1-0 control and MTAP-knockout cells pretreated with 10 μM of MG132 for 3 h. Arrow marks the site of vimentin. Data shown are representative of three independent experiments.

B Western blot analysis of endogenous vimentin levels in H1650 control and PRMT5-silencing cells treated with 50 μg/ml cycloheximide (CHX). Right: the intensity of vimentin signals was quantified by ImageJ software, normalized to the internal control β-actin, then normalized to zero time point, and plotted against time points (mean ± SE, $n = 4$, biological replicates).
