# Supplementary figures and images for: Reduced symmetric dimethylation stabilizes vimentin and promotes metastasis in MTAP‐deficient lung cancer
Source: EMBO Rep. 2022 Jun 29;23(8):e54265. doi: 10.15252/embr.202154265 (PMC9346486; doi:10.15252/embr.202154265)

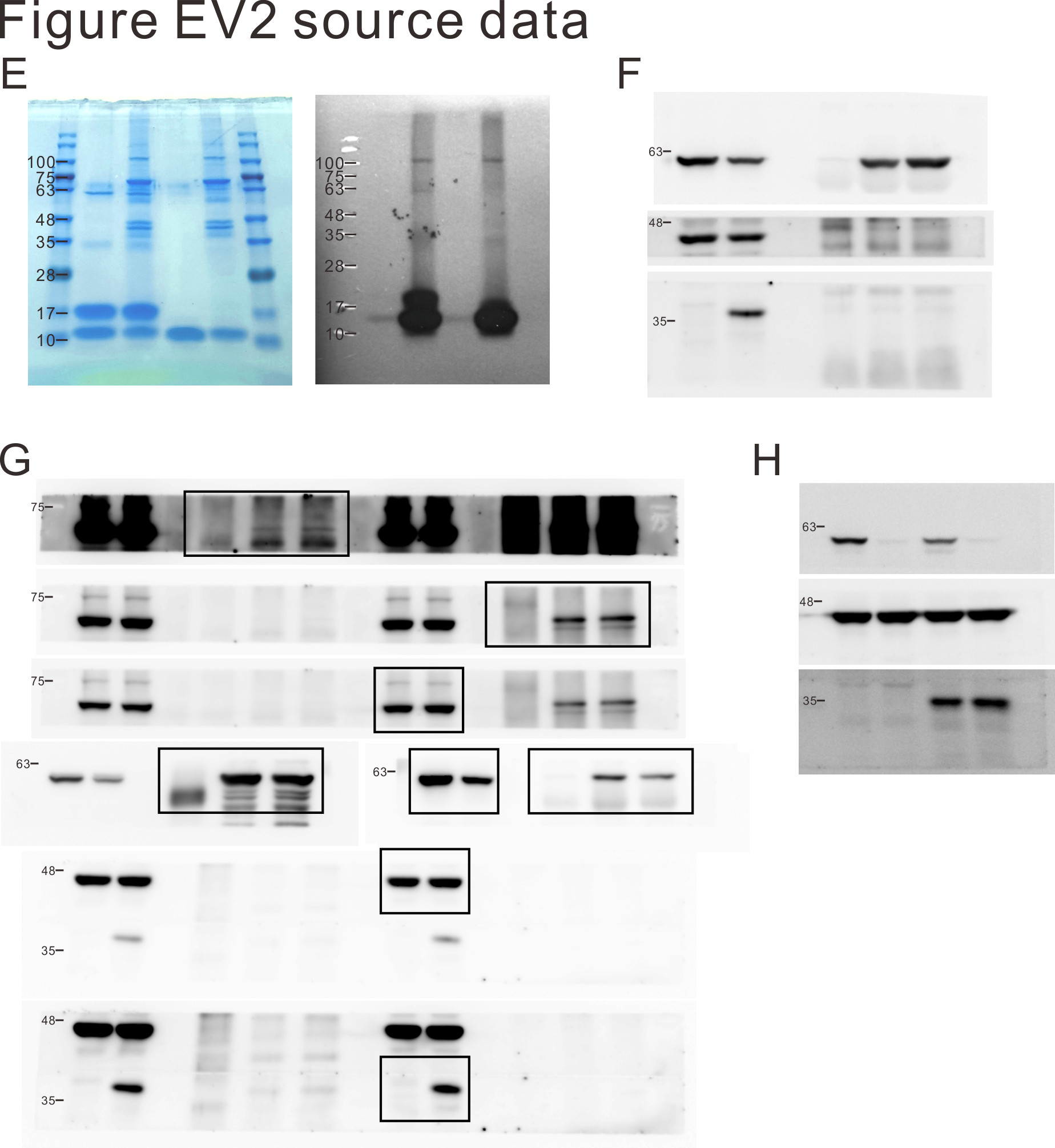

Supplement: Supplementary file 3 — Source Data for Expanded View [file EMBR-23-e54265-s001.tif]
