# Supplementary material for: Reduced symmetric dimethylation stabilizes vimentin and promotes metastasis in MTAP‐deficient lung cancer
Source: EMBO Rep. 2022 Jun 29;23(8):e54265. doi: 10.15252/embr.202154265 (PMC9346486; doi:10.15252/embr.202154265)
Supplement: Supplementary file 4 [file EMBR-23-e54265-s004.pdf]

EMBO reports

Reduced symmetric dimethylation stabilizes vimentin and promotes metastasis in MTAP deficient lung cancer

Wen-Hsin Chang, Yi-Ju Chen, Yi-Jing Hsiao, Ching-Cheng Chiang, Chia-Yu Wang, Ya-Ling Chang, Qi-Sheng Hong, Chien-Yu Lin, Shr-Uen Lin, Gee-Chen Chang, Hsuan-Yu Chen, Yu-Ju Chen, Ching-Hsien Chen, Pan-Chyr Yang, and Sung-Liang Yu
DOI: 10.15252/embr.202154265

Corresponding author(s): Sung-Liang Yu (slyu@ntu.edu.tw) , Pan-Chyr Yang (pcyang@ntu.edu.tw), Ching-Hsien Chen (jchchen@ucdavis.edu)

Review Timeline:

Submission Date:	3rd Nov 21
Editorial Decision:	9th Dec 21
Revision Received:	9th Apr 22
Editorial Decision:	9th May 22
Revision Received:	23rd May 22
Accepted:	8th Jun 22

Editor: Deniz Senyilmaz Tiebe

Transaction Report:

(Note: With the exception of the correction of typographical or spelling errors that could be a source of ambiguity, letters and reports are not edited. Depending on transfer agreements, referee reports obtained elsewhere may or may not be included in this compilation. Referee reports are anonymous unless the Referee chooses to sign their reports.)

1st Editorial Decision 9th Dec 2021

Dear Dr. Yu,

Thank you for the submission of your research manuscript to our journal, which was now seen by three referees, whose reports are copied below.

We concur with the referees that the proposed role of PRMT5 mediated vimentin demethylation in MTAP deficient cancer cells is in principle very interesting. However, referees, especially referee #1, raise significant concerns that need to be addressed to consider publication here.

I find the reports informed and constructive, and believe that addressing the concerns raised will significantly strengthen the manuscript. As the reports are below, and I think all points need to be addressed, I will not detail them here.

Should you be able to address all referee concerns satisfactorily, we would like to invite you to revise your manuscript with the understanding that the referee concerns (as in their reports) must be fully addressed and their suggestions taken on board. Please address all referee concerns in a complete point-by-point response. Acceptance of the manuscript will depend on a positive outcome of a second round of review. It is EMBO reports policy to allow a single round of revision only and acceptance or rejection of the manuscript will therefore depend on the completeness of your responses included in the next, final version of the manuscript.

We generally allow three months as standard revision time. As a matter of policy, competing manuscripts published during this period will not negatively impact on our assessment of the conceptual advance presented by your study. However, we request that you contact the editor as soon as possible upon publication of any related work, to discuss how to proceed. Should you foresee a problem in meeting this three-month deadline, please let us know in advance and we may be able to grant an extension.

*** Temporary update to EMBO Press scooping protection policy:
We are aware that many laboratories cannot function at full efficiency during the current COVID-19/SARS-CoV-2 pandemic and have therefore extended our 'scooping protection policy' to cover the period required for a full revision to address the experimental issues highlighted in the editorial decision letter. Please contact the scientific editor handling your manuscript to discuss a revision plan should you need additional time, and also if you see a paper with related content published elsewhere.***

IMPORTANT NOTE: we perform an initial quality control of all revised manuscripts before re-review. Your manuscript will FAIL this control and the handling will be DELAYED if the following APPLIES:
1. A data availability section providing access to data deposited in public databases is missing (where applicable).
2. Your manuscript contains statistics and error bars based on n=2. Please use scatter plots in these cases.

You can submit the revision either as a Scientific Report or as a Research Article. For Scientific Reports, the revised manuscript can contain up to 5 main figures and 5 Expanded View figures. If the revision leads to a manuscript with more than 5 main figures it will be published as a Research Article. In this case the Results and Discussion section should be separate. If a Scientific Report is submitted, these sections have to be combined. This will help to shorten the manuscript text by eliminating some redundancy that is inevitable when discussing the same experiments twice. In either case, all materials and methods should be included in the main manuscript file

Supplementary/additional data: The Expanded View format, which will be displayed in the main HTML of the paper in a collapsible format, has replaced the Supplementary information. You can submit up to 5 images as Expanded View. Please follow the nomenclature Figure EV1, Figure EV2 etc. The figure legend for these should be included in the main manuscript document file in a section called Expanded View Figure Legends after the main Figure Legends section. Additional Supplementary material should be supplied as a single pdf labeled Appendix. The Appendix includes a table of content on the first page with page numbers, all figures and their legends. Please follow the nomenclature Appendix Figure Sx throughout the text and also label the figures according to this nomenclature. For more details please refer to our guide to authors.

Please note that for all articles published beginning 1 July 2020, the EMBO Reports reference style will change to the Harvard style for all article types. Details and examples are provided at
https://www.embopress.org/page/journal/14693178/authorguide#referencesformat

When submitting your revised manuscript, please carefully review the instructions that follow below. Failure to include requested items will delay the evaluation of your revision.

1) a .docx formatted version of the manuscript text (including legends for main figures, EV figures and tables). Please make sure that the changes are highlighted to be clearly visible.

2) individual production quality figure files as .eps, .tif, .jpg (one file per figure).

3) a .docx formatted letter INCLUDING the reviewers' reports and your detailed point-by-point responses to their comments. As part of the EMBO Press transparent editorial process, the point-by-point response is part of the Review Process File (RPF), which will be published alongside your paper. For more details on our Transparent Editorial Process, please visit our website: https://www.embopress.org/page/journal/14693178/authorguide#transparentprocess
You are able to opt out of this by letting the editorial office know (emboreports@embo.org). If you do opt out, the Review Process File link will point to the following statement: "No Review Process File is available with this article, as the authors have chosen not to make the review process public in this case."

4) a complete author checklist, which you can download from our author guidelines (<http://embor.embopress.org/authorguide>). Please insert information in the checklist that is also reflected in the manuscript. The completed author checklist will also be part of the RPF.

5) Please note that all corresponding authors are required to supply an ORCID ID for their name upon submission of a revised manuscript (<https://orcid.org/>). Please find instructions on how to link your ORCID ID to your account in our manuscript tracking system in our Author guidelines (<http://embor.embopress.org/authorguide>).

6) We replaced Supplementary Information with Expanded View (EV) Figures and Tables that are collapsible/expandable online. A maximum of 5 EV Figures can be typeset. EV Figures should be cited as 'Figure EV1, Figure EV2" etc... in the text and their respective legends should be included in the main text after the legends of regular figures.

- For the figures that you do NOT wish to display as Expanded View figures, they should be bundled together with their legends in a single PDF file called *Appendix*, which should start with a short Table of Content. Appendix figures should be referred to in the main text as: "Appendix Figure S1, Appendix Figure S2" etc. See detailed instructions regarding expanded view here: <http://embor.embopress.org/authorguide#expandedview>.

- Additional Tables/Datasets should be labeled and referred to as Table EV1, Dataset EV1, etc. Legends have to be provided in a separate tab in case of .xls files. Alternatively, the legend can be supplied as a separate text file (README) and zipped together with the Table/Dataset file.

7) We would also encourage you to include the source data for figure panels that show essential data.

Numerical data should be provided as individual .xls or .csv files (including a tab describing the data). For blots or microscopy, uncropped images should be submitted (using a zip archive if multiple images need to be supplied for one panel). Additional information on source data and instruction on how to label the files are available <http://embor.embopress.org/authorguide#sourcedata>.

8) Our journal encourages inclusion of *data citations in the reference list* to directly cite datasets that were re-used and obtained from public databases. Data citations in the article text are distinct from normal bibliographical citations and should directly link to the database records from which the data can be accessed. In the main text, data citations are formatted as follows: "Data ref: Smith et al, 2001" or "Data ref: NCBI Sequence Read Archive PRJNA342805, 2017". In the Reference list, data citations must be labeled with "[DATASET]". A data reference must provide the database name, accession number/identifiers and a resolvable link to the landing page from which the data can be accessed at the end of the reference. Further instructions are available at <http://embor.embopress.org/authorguide#datacitation>.

9) Please make sure to include a Data Availability Section before submitting your revision - if it is not applicable, make a statement that no data were deposited in a public database. Primary datasets (and computer code, where appropriate) produced in this study need to be deposited in an appropriate public database (see <http://embor.embopress.org/authorguide#dataavailability>).

Please remember to provide a reviewer password if the datasets are not yet public.

The accession numbers and database should be listed in a formal "Data Availability " section (placed after Materials & Method) that follows the model below. Please note that the Data Availability Section is restricted to new primary data that are part of this study.

Data availability

The datasets (and computer code) produced in this study are available in the following databases:

- RNA-Seq data: Gene Expression Omnibus GSE46843 (https://www.ncbi.nlm.nih.gov/geo/query/acc.cgi?acc=GSE46843)
- [data type]: [name of the resource] [accession number/identifier/doi] ([URL or identifiers.org/DATABASE:ACCESSION])

*** Note - All links should resolve to a page where the data can be accessed. ***

10) Regarding data quantification, please ensure to specify the name of the statistical test used to generate error bars and P values, the number (n) of independent experiments underlying each data point (not replicate measures of one sample), and the test used to calculate p-values in each figure legend. Discussion of statistical methodology can be reported in the materials and methods section, but figure legends should contain a basic description of n, P and the test applied.
Please note that error bars and statistical comparisons may only be applied to data obtained from at least three independent biological replicates.
Please also include scale bars in all microscopy images.

We would also welcome the submission of cover suggestions, or motifs to be used by our Graphics Illustrator in designing a cover.

I look forward to seeing a revised version of your manuscript when it is ready. Please let me know if you have questions or comments regarding the revision.

Kind regards,

Deniz Senyilmaz Tiebe

Deniz Senyilmaz Tiebe, PhD
Editor
EMBO Reports

Referee #1:

Summary:
In this study, the group led by Sung-Liang Yu show that MTAP loss promotes invasion and metastasis in long cancer. Mass Spec. studies identified vimentin as a PRMT5 substrate that displays decreased SDMA in the absence of MTAP. MTAP-loss or PRMT5 knockdown results in vimentin stabilization, suggesting that the SDMA marking of vimentin promotes its destabilization. Indeed, the authors go on so show that vimentin becomes highly ubiquitinated in a SDMA-dependent manner. Finally, they show that MTAP/PRMT5 levels negatively correlated with vimentin levels in lung cancer samples.

Critique:
The key findings in this study are; First, is that MTAP loss promotes metastasis. Second, PRMT5 methylates vimentin. Third, vimentin is destabilized by PRMT5 methylation. Each of these findings are significant, but a number of points are still preliminary and addition experiments are needed to strengthen the overall hypothesis, before this study is suitable for publication in EMBO Reports.

The following needs to be addressed:

1. In Figure 2, the Western blot evidence showing vimentin is a PRMT5 substrate is very weak. Figure 2C needs to be repeated. This is a key figure, and all you see is a smudge using the SYM10 antibody. Also, the decrease in SDMA signal after PRMT5 siRNA (shown in Figure 2D) is not convincing. Ideally, the in vitro methylation experiment should be performed using radioactive AdoMet. Known substrates for PRMT5 (like SmB or histones) should be used as a positive control, which will allow us to determine how good a PRMT5 substrate vimentin is, compared to know PRMT5 targets.

2. According the "Material and Methods" section, the SDMA antibody used in the study was Sym10. This antibody was raised against a GAR motif (an RGG repeat). The 4 vimentin methylation sites identified in Figure 3 do not harbor GAR motifs. Thus, additional studies are needed to confirm that these four sites are indeed key PRMT5 targets. Two independent approaches should be taken here: 1) does the Sym10 antibody recognize peptides that harbor SDMA peptides for R196, R207, R345 and R364? The non-methylated peptide controls should not be recognized by this antibody. This experiment can be performed as a dot-blot. 2) Ideally, the authors will raise specific antibodies to the four PRMT5 sites.

3. In Figures 4 & 5, the authors introduce different mutations (K & F) into the vimentin methylation motifs. They refer to the R-to-F mutation as a methyl-memetic. However, the R-to-F mutation is not a methyl-memetic, even though there are a few publications that refer to it as such. Here I will highlight the reasons why the R-to-F mutation is not a methyl-memetic:
a. In the first place, methylated arginine residues retain a full positive charge on the side chain - there are no charges on the phenylalanine side chain.

b. In the MMA side chain there are four hydrogen bond donors and in the ADMA/SDMA side chain there are three hydrogen bond donors - there are none in the phenylalanine side chain.
c. Just in terms of carbon atoms, the phenylalanine side chain has seven; the MMA side chain has only five and the ADMA/SDMA side chain has only six.
d. In terms of the overall number of non-hydrogen atoms, the phenylalanine side chain has seven, the MMA side chain has eight and the ADMA/SDMA has nine.
e. There is no evidence that R-to-F mutation can be read by Tudor domains. So, the docking properties of the Rme reader domains are also not generated by this R-to-F mutation.

There is no need to remove the data that includes experiments with the R-to-F mutation as this mutant often behaves differently to the R-to-G and R-to-K mutants and does provide interesting data. However, all reference to the R-to-F mutation being a methyl-memetic should be removed from the text.

4. The emerging role of PRMT/proteosome crosstalk should be addressed in the discussion. There are a number of published studies related to this topic. Some works show that the SDMA PTM promotes protein stability, and others show that it stimulates ubiquitination.

5. The data in Figure 6 cannot be viewed. The authors should not use a black background for this figure.

6. Finally, is there a mechanistic explanation for the SDMA mark being a signal for protein degradation? Is there an E3 ligase that can read the SDMA marks on vimentin. This is a critical missing link in this study.

Referee #2:

The authors identify the critical role MTAP/PRMT5 axis in post-transcriptional regulation of vimentin, which is a novel target for this post-translational modification/dimethylation. MTAP is responsible for catalyzing phosphorylation of MTA, so MTAP deficiency results in MTA accumulation, which reduces the level of PRMT5 (an arginine methyltransferase), which is required for demethylation of vimentin. This study uses methylproteomic screening to identify vimentin as novel dimethyl protein and cell and animal based assays with genetic knockout and post-transcriptional modification to test prove this mechanism of vimentin modification and its relevance to lung cancer progression. Overall, this study is very well written and the data very clearly illustrates the importance of their findings. In the abstract the authors say sDMA modification trivially affects the filamentous structure of vimentin and I would say this has not been fully proven, since only molecular methods were used to study vimentin structure. This is a small point that can be addressed through high resolution confocal microscopy experiments or other high-resolution imaging or structural analysis.

Referee #3:

In this well-written and insightful manuscript by Chang et al., the authors investigated the repressive role MTAP plays in aggressive lung cancer and tumour models. It has been established that PRMT5 activity is negatively impacted by buildup of MTA when MTAP levels are low or absent. Through proteomics, they identified vimentin as a PRMT5 substrate; when vimentin is symmetrically methylated by PRMT5 on four unique residues, vimentin is degraded in the proteasome and is less likely to form filaments that promote cell invasion. The authors use several methods described in detail as to how MTAP/PRMT5 axis affects vimentin stability, and they highlight the importance of vimentin as a target and corroborate their findings by comparing overall survival in patients that are either MTAP high/ vimentin low or MTAP low/ vimentin high. This reviewer rates this manuscript as very high and impactful to PRMT and cancer fields as it represents a significant advance.

Very Minor:
(1) Associated error for average fold change intensities for metabolites in Fig. S2a?
(2) Bottom of page 11: A more accurate statement would be that the residue identity at these methylation sites governs the migration promotion of vimentin since sDMA is not being directly measured but correlated.
(3) "... nutrient-responsive PTMs on proteins or chromatins modulate..." chromatin without an "s" on page 18.
(4) Top of page 19: "... the major type II PRMTs" PRMT without an "s"

Comments from the referees:

Referee #1:

Summary:
In this study, the group led by Sung-Liang Yu show that MTAP loss promotes invasion and metastasis in long cancer. Mass Spec. studies identified vimentin as a PRMT5 substrate that displays decreased SDMA in the absence of MTAP. MTAP-loss or PRMT5 knockdown results in vimentin stabilization, suggesting that the SDMA marking of vimentin promotes its destabilization. Indeed, the authors go on so show that vimentin becomes highly ubiquitinated in a SDMA-dependent manner. Finally, they show that MTAP/PRMT5 levels negatively correlated with vimentin levels in lung cancer samples.

Critique:
The key findings in this study are; First, is that MTAP loss promotes metastasis. Second, PRMT5 methylates vimentin. Third, vimentin is destabilized by PRMT5 methylation. Each of these findings are significant, but a number of points are still preliminary and addition experiments are needed to strengthen the overall hypothesis, before this study is suitable for publication in EMBO Reports.

The following needs to be addressed:

C1. *In Figure 2, the Western blot evidence showing vimentin is a PRMT5 substrate is very weak. Figure 2C needs to be repeated. This is a key figure, and all you see is a smudge using the SYM10 antibody. Also, the decrease in SDMA signal after PRMT5 siRNA (shown in Figure 2D) is not convincing. Ideally, the in vitro methylation experiment should be performed using radioactive AdoMet. Known substrates for PRMT5 (like SmB or histones) should be used as a positive control, which will allow us to determine how good a PRMT5 substrate vimentin is, compared to know PRMT5 targets.*

R1: We would like to thank the reviewer for the constructive and helpful suggestions. In accordance with the suggestions, we have included histone H3 and H4 proteins as positive controls and utilized tritiated AdoMet as methyl donor in *in vitro* methylation assays. The fluorography data showed that vimentin can be methylated by PRMT5 even in the presence of dominant substrates, histone H3 and H4, albeit the signal is relatively weak due to the less amount of its input. These results suggest that vimentin serves as a favorable substrate of PRMT5. The corresponding Results (page 9) and Materials and Methods (page 27) were revised and the changes were indicated in blue.

<u>Page 9, line 16 to line 22</u>

To determine whether vimentin is a substrate of PRMT5, we performed an *in vitro*

methyltransferase assays in which the recombinant PRMT5 and vimentin were incubated with methyl donor S-adenosylmethionine (SAM). We found that vimentin was directly methylated by PRMT5 and the sDMA level of vimentin was mostly abolished by MTA, which is an analog of SAM and competitive inhibitor of methyltransferases (Kryukov et al., 2016) (Figs 2C and EV2E).

Revised Figure EV2E

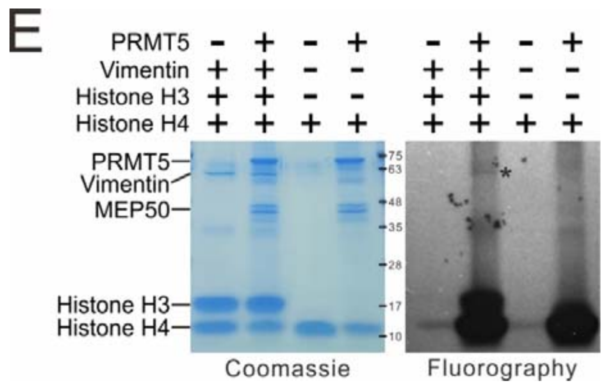

E. *In vitro* methylation of vimentin by PRMT5. Tritiated proteins were separated by SDS-PAGE, stained with Coomassie blue (left), dried and analyzed by fluorography (right). Histone H3 and H4 proteins were used as positive controls. *: tritiated vimentin.

Page 27, line 23 to Page 28, line 5

For fluorography, an *in vitro* methyltransferase assay using radioactive SAM (NET155V250UC, Perkin Elmer) was also carried out as follows. 1 μg PRMT5 and 1 μg substrate proteins (Histone H3, ab198757; Histone H4, ab198115; Abcam) were incubated in the presence of 3 μCi tritiated SAM (18.0 Ci/mmol from a 1.0 mCi/ml stock solution) for 1 hour at 30°C in a final volume of 30 μl methylation reaction buffer. Subsequently, tritiated protein was separated by SDS-PAGE and then the gel was treated with EN^{3}HANCE (6NE9701, Perkin Elmer) and exposed to a film at -80°C for 7 days.

C2. *According the "Material and Methods" section, the SDMA antibody used in the study was Sym10. This antibody was raised against a GAR motif (an RGG repeat). The 4 vimentin methylation sites identified in Figure 3 do not harbor GAR motifs. Thus, additional studies are needed to confirm that these four sites are indeed key PRMT5 targets. Two independent approaches should be taken here: 1) does the Sym10 antibody recognize peptides that harbor SDMA peptides for R196, R207, R345 and R364? The non-methylated peptide controls should*

not be recognized by this antibody. This experiment can be performed as a dot-blot. 2) Ideally, the authors will raise specific antibodies to the four PRMT5 sites.

R2: We thank the reviewer for the insightful comments. To alleviate the concerns raised by the reviewer, we used synthetic di-methylated and non-methylated vimentin peptides to clarify the specificity of the SYM10 antibody by performing dot blot assays. We found that the dot intensity of sDMA from MTAP-knockout cell lysates (H1650 sgMTAP cell lysate) is weaker than that of the one from control cell lysates (H1650 sgCTRL cell lysate), consistent with the Western blot data (Figure 2B). In addition, the full length vimentin recombinant protein used in *in vitro* methylation assays and non-methylated peptides (R196/R207 unmodified and R345/R364 unmodified) were not recognized by the SYM10 antibody, whereas synthetic di-methylated peptides (R196 me2s, R207 me2s, R196/R207 me2s, R345 me2s, R364 me2s, R345/R364 me2s) were reacted with the SYM10 antibody, supporting our notion that these four sites are key PRMT5 targets (Figure 3). Indeed, we agree with the reviewer that generating specific methyl-vimentin antibodies would further confirm the four PRMT5 sites; however, it usually takes 4-6 months to produce a new antibody, and, in particular, we received synthetic peptides in the end of February in the wake of COVID-19; therefore, performing this additional study would have delayed this resubmission beyond the allotted time. The dot blot data were included in the Results section (page 11) and marked in blue.

Page 11, line 1 to line 2

The dimethylated forms of the four arginine residues can be recognized by an anti-sDMA antibody (Fig EV3B).

Revised Figure EV3B

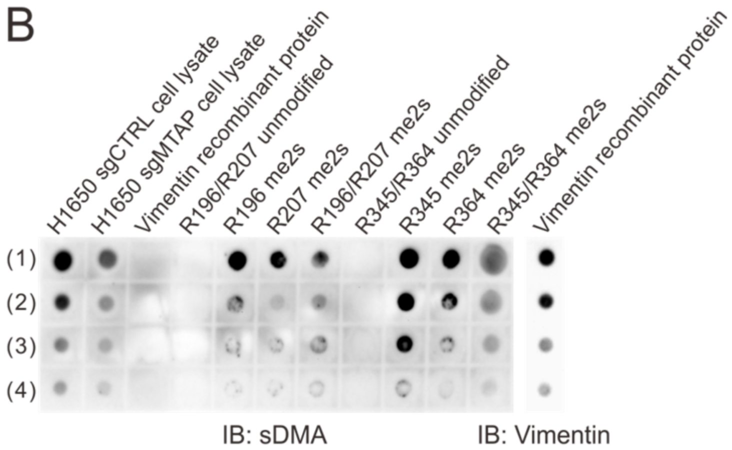

R196/R207 unmodified	EMLQREEAENTLQSFRQDVDC
R196 me2s	EMLQ$R_{196(me2s)}$EEAENTLQSFRQDVDC
R207 me2s	EMLQREEAENTLQSF$R_{207(me2s)}$QDVDC
R196/R207 me2s	EMLQ$R_{196(me2s)}$EEAENTLQSF$R_{207(me2s)}$QDVDC
R345/R364 unmodified	CRQMREMEENFAVEAANYQDTIGRLQD
R345 me2s	CRQM$R_{345(me2s)}$EMEENFAVEAANYQDTIGRLQD
R364 me2s	CRQMREMEENFAVEAANYQDTIG$R_{364(me2s)}$LQD
R345/R364 me2s	CRQM$R_{345(me2s)}$EMEENFAVEAANYQDTIG$R_{364(me2s)}$LQD

B. Dot blot analysis showing specificity of sDMA antibody. H1650 cell lysates were used as positive controls. The sequences of non-methylated (unmodified) and dimethylated (me2s) vimentin peptides were shown in the bottom. H1650 cell lysates: (1) 6.5 ug, (2) 3.2 ug, (3) 1.6 ug, (4) 0.8 ug. Vimentin recombinant protein: (1) 0.5 ug, (2) 0.25 ug, (3) 0.125 ug, (4) 0.0625 ug. Vimentin peptides: (1) 5 ug, (2) 2.5 ug, (3) 1.25 ug, (4) 0.625 ug.

C3. In Figures 4 & 5, the authors introduce different mutations (K & F) into the vimentin methylation motifs. They refer to the R-to-F mutation as a methyl-memetic. However, the R-to-F mutation is not a methyl-memetic, even though there are a few publications that refer to it as such. Here I will highlight the reasons why the R-to-F mutation is not a methyl-memetic:

a. In the first place, methylated arginine residues retain a full positive charge on the side chain - there are no charges on the phenylalanine side chain.

b. In the MMA side chain there are four hydrogen bond donors and in the ADMA/SDMA side chain there are three hydrogen bond donors - there are none in the phenylalanine side chain.

c. Just in terms of carbon atoms, the phenylalanine side chain has seven; the MMA side chain has only five and the ADMA/SDMA side chain has only six.

d. In terms of the overall number of non-hydrogen atoms, the phenylalanine side chain has seven, the MMA side chain has eight and the ADMA/SDMA has nine.

e. There is no evidence that R-to-F mutation can be read by Tudor domains. So, the docking

properties of the Rme reader domains are also not generated by this R-to-F mutation.

There is no need to remove the data that includes experiments with the R-to-F mutation as this mutant often behaves differently to the R-to-G and R-to-K mutants and does provide interesting data. However, all reference to the R-to-F mutation being a methyl-memetic should be removed from the text.

R3: We would like to thank the reviewer for the comments. Yes, we agree that R-to-F mutant is not a perfect candidate to be a methyl-mimetic. The reason why researchers in previous studies used phenylalanine (F) is that phenylalanine (F) mutation introduced hydrophobicity, partially mimicking the bulky hydrophobic properties of methylated arginine residues. To better reflect the functionality of R-to-F mutant, we have removed the term "methylation-mimic" and revised the description for 4RF mutant in the Results section (pages 11 and 14), and have marked these changes in blue.

Page 11, line 3 to line 5

To verify whether these four arginines are the key dimethylation sites of vimentin, these arginines were individually or entirely mutated to lysines (non-arginine methylatable).

Page 11, line 15 to line 19

To investigate if the functionality of vimentin is mediated by its sDMA level, endogenous vimentin in H1650 and CL1-5 cells was knocked out by CRISPR/Cas9 approaches and the effect of wild-type, 4RK and 4RF (R196/207/345/364F, mimicking the bulky hydrophobic properties of methylated arginines) mutant vimentin on cell motility was evaluated.

Page 14, line 11 to line 13

As shown in Fig 5F, the unmethylatable 4RK mutant vimentin had a prolonged half-life compared with wild-type and 4RF mutant.

C4. The emerging role of PRMT/proteosome crosstalk should be addressed in the discussion. There are a number of published studies related to this topic. Some works show that the SDMA PTM promotes protein stability, and others show that it stimulates ubiquitination.

R4: We would like to thank the reviewer for the constructive comments. We have accordingly revised our discussion to address this issue. Please see changes in the Discussion section indicated in blue (page 19).

Page 19, line 1 to line 9

In addition to the interplay with metabolites, the crosstalk between PTMs also influences the signal transduction and biological functions. Recently, there are a number of studies focusing on protein methylation-dependent ubiquitination and degradation. After being methylated by methyltransferase, the stability of substrate proteins would be either upregulated or downregulated, thereby strengthening or alleviating the involved pathways and functions such as alterations in lipogenesis, angiogenesis, metastasis, genome stability and carcinogenesis (Hu et al, 2015; Kim et al, 2016; Leng et al, 2018; Li et al, 2020; Liu et al, 2020; Liu et al, 2016). Therefore, protein methylation appears to be a new hallmark for protein stability.

C5. The data in Figure 6 cannot be viewed. The authors should not use a black background for this figure.

R5: We thank the reviewer for bringing this to our attention. We downloaded the files we previously submitted from the EMBO submission system and checked the content. The Figure 6 can be viewed as a TIF file. To address the concerns, we have re-exported the Figure 6 and pasted a copy below.

Figure 6

Figure 6

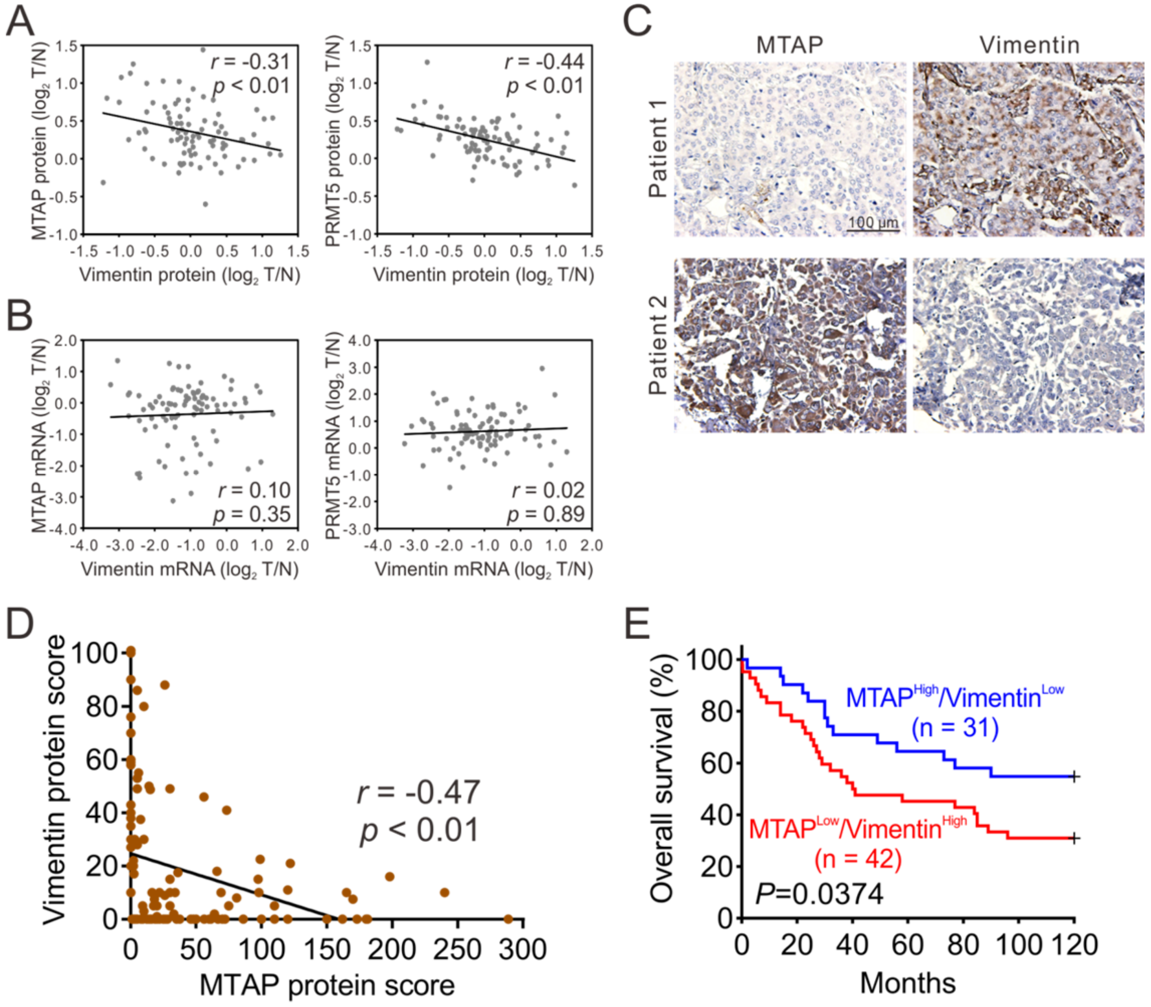

Figure 6. Negative association between MTAP/PRMT5 and vimentin is confirmed in lung cancer patients

A-B. The correlation of MTAP and vimentin or PRMT5 and vimentin at protein (A) and mRNA (B) levels from 89 lung adenocarcinoma patients. *r* is the Spearman's rank correlation coefficient.

C. Representative images of IHC staining.

D. An association between MTAP and vimentin levels in 124 lung cancer patients was examined by Spearman's rank correlation.

E. Kaplan-Meier analyses of overall survival for lung cancer patients grouped by MTAP and vimentin levels from IHC staining. *p* value was obtained by log-rank test.

C6. Finally, is there a mechanistic explanation for the SDMA mark being a signal for protein degradation? Is there an E3 ligase that can read the SDMA marks on vimentin. This is a

critical missing link in this study.

R6: The reviewer's suggestion is well taken and we have included the potential explanation in the Discussion section. The related descriptions were included in the Discussion section (page 19) and marked in blue.

<u>Page 19, line 9 to line 20</u>

How can methylation modification be a signal for protein degradation? Since PTMs can lead to protein conformational change, the interaction accessibility of methylated proteins to E3 ligases or deubiquitinases would be altered (Yang et al, 2009). Two E3 ligases, TRIM16 and RNF208, were previously reported as negative regulators of vimentin (Marshall et al, 2010; Pang et al, 2019; Tian et al, 2020), but it is unclear whether their interaction with vimentin would be interfered by sDMA modification. Moreover, their targeting sites for ubiquitin conjugation on vimentin is outside of the coiled-coil rod domain, which is the major dimethylated region and responsible for arginine methylation-dependent ubiquitination in this study. The detailed regulation mechanisms could be elucidated by exploring the ubiquitome in the context of wild-type or 4RK vimentin.

Referee #2:

The authors identify the critical role MTAP/PRMT5 axis in post-transcriptional regulation of vimentin, which is a novel target for this post-translational modification/dimethylation. MTAP is responsible for catalyzing phosphorylation of MTA, so MTAP deficiency results in MTA accumulation, which reduces the level of PRMT5 (an arginine methyltransferase), which is required for demethylation of vimentin. This study uses methylproteomic screening to identify vimentin as novel dimethyl protein and cell and animal based assays with genetic knockout and post-transcriptional modification to test prove this mechanism of vimentin modification and its relevance to lung cancer progression. Overall, this study is very well written and the data very clearly illustrates the importance of their findings.

C1. In the abstract the authors say sDMA modification trivially affects the filamentous structure of vimentin and I would say this has not been fully proven, since only molecular methods were used to study vimentin structure. This is a small point that can be addressed through high resolution confocal microscopy experiments or other high-resolution imaging or structural analysis.

R1: We would like to thank the reviewer for the constructive comments. In accordance with the suggestions, we have repeated the immunofluorescence experiments in order to confirm our findings and have revised Figures 4A and EV4A accordingly. The related methodology in the Materials and Methods section (page 27) has also been revised.

Page 29, line 6 to line 8

Fluorescent images were taken by a confocal microscope (Carl Zeiss LSM880, Oberkochen, Germany) with Airyscan detector and were processed by ZEISS ZEN2 image software.

Revised Figure 4A

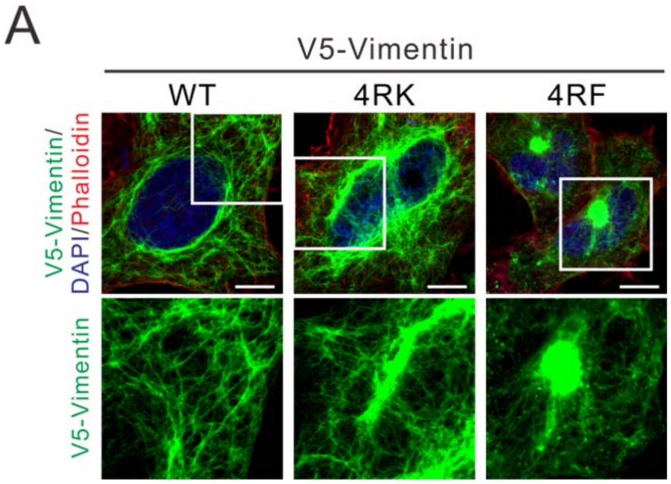

A. Immunofluorescence staining of V5-vimentin wild-type, 4RK and 4RF mutants expressed in CL1-0 cells. F-actin was stained with phalloidin, and nucleus was stained with DAPI. Scale bars, 10 μm. Data shown are representative of three independent experiments.

<u>Revised Figure EV4A</u>

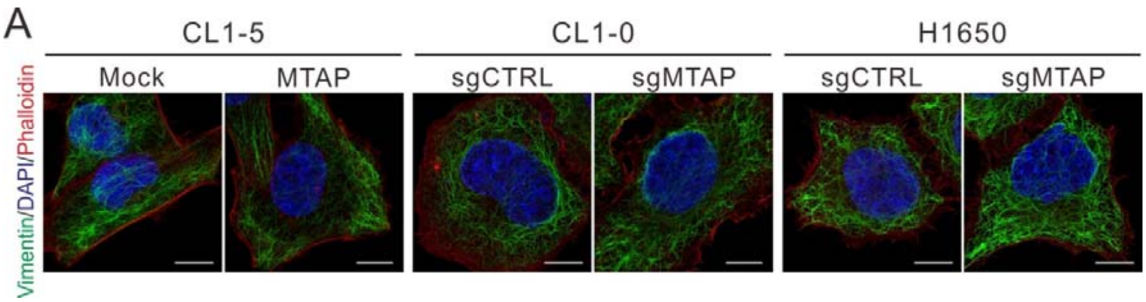

A. Immunofluorescence staining of vimentin in indicated cell lines. F-actin was stained with phalloidin, and nucleus was stained with DAPI. Scale bar, 10 μm. Data shown are representative of three independent experiments.

Referee #3:

In this well-written and insightful manuscript by Chang et al., the authors investigated the repressive role MTAP plays in aggressive lung cancer and tumour models. It has been established that PRMT5 activity is negatively impacted by buildup of MTA when MTAP levels are low or absent. Through proteomics, they identified vimentin as a PRMT5 substrate; when vimentin is symmetrically methylated by PRMT5 on four unique residues, vimentin is degraded in the proteasome and is less likely to form filaments that promote cell invasion. The authors use several methods described in detail as to how MTAP/PRMT5 axis affects vimentin stability, and they highlight the importance of vimentin as a target and corroborate their findings by comparing overall survival in patients that are either MTAP high/ vimentin low or MTAP low/ vimentin high. This reviewer rates this manuscript as very high and impactful to PRMT and cancer fields as it represents a significant advance.

Very Minor:

C1. Associated error for average fold change intensities for metabolites in Fig. S2a?

R1: We thank the reviewer for the helpful suggestion. We have revised the Figure EV2A and related figure legends and marked in blue (page 49).

Revised Figure EV2A

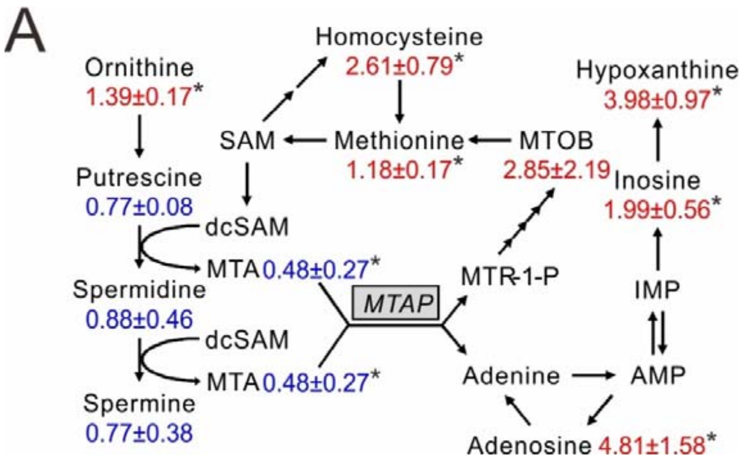

A. MTAP-mediated metabolic alterations in polyamine, methionine and adenine salvage pathways. The numbers are the average fold change intensities and associated errors for metabolites of CL1-5 MTAP/CL1-5 Mock (Student *t* test, n=3, biological replicates, $*p < 0.05$).

C2. Bottom of page 11: A more accurate statement would be that the residue identity at these methylation sites governs the migration promotion of vimentin since sDMA is not being directly measured but correlated.

R2: The reviewer's suggestion is well taken and we have revised the corresponding descriptions in the Results section (page 11, Figs 3G and EV3D) and marked in blue.

<u>Page 11, line 19 to line 24</u>

Data from single-cell tracking migration assays showed that the migration rate of vimentin-knockout cells was reduced and rescued by restoration of wild-type vimentin. Surprisingly, 4RF mutant failed to rescue cell migration, while 4RK mutant exhibited greater migration ability than wild-type vimentin (Figs 3G and EV3D), indicating that the four arginine residues identified govern the migration promotion of vimentin.

C3. "... nutrient-responsive PTMs on proteins or chromatins modulate..." chromatin without an "s" on page 18.

R3: We thank the reviewer for reminding us the oversight. We have removed the "s" in the Discussion section (page 18).

<u>Page 18, line 15 to line 18</u>

On the other hand, nutrient-responsive PTMs on proteins or chromatin modulate the expression of genes and proteins involved in metabolism, forming a feedback or feedforward loop (Campbell & Wellen, 2018).

C4. Top of page 19: "... the major type II PRMTs" PRMT without an "s"

R4: We thank the reviewer for pointing out the oversight. We have removed the "s" in the Discussion section (page 19).

<u>Page 19, line 21 to line 23</u>

PRMT5, the major type II PRMT, plays an indispensable role in the regulation of developmental and physiological processes as the global deletion of PRMT5 in mice results in embryonic lethality (Guccione & Richard, 2019).

1st Revision - Editorial Decision **9th May 2022**

Dear Prof. Yu,

Thank you for submitting your revised manuscript. It has now been seen by one of the original referees.

As you can see, the referee finds that the study is significantly improved during revision and recommends publication. However, the referee has remaining concerns that need to be addressed prior to publication. Please repeat the experiment in Figure 2C and replace it with a higher quality blot as requested by the referee.

Moreover, I need you to address the editorial points below.

• We updated our journal's competing interests policy in January 2022 and request authors to consider both actual and perceived competing interests. Please review the policy https://www.embopress.org/competing-interests and update your competing interests if necessary. Also, please rename the 'Conflict of Interests' section as 'Disclosure statement and competing interests'.
• Regarding the Author Contributions, we now use CRediT to specify the contributions of each author in the journal submission system. CRediT replaces the author contribution section. Please use the free text box to provide more detailed descriptions. See also guide to authors https://www.embopress.org/page/journal/14693178/authorguide#authorshipguidelines.
• Please make the datasets GSE160522 and PXD031192 publicly available and remove the reviewer passwords from the manuscript.
• Papers published in EMBO Reports include a 'synopsis' and 'bullet points' to further enhance discoverability. Both are displayed on the html version of the paper and are freely accessible to all readers. The synopsis includes a short standfirst summarizing the study in 1 or 2 sentences that summarize the paper and are provided by the authors and streamlined by the handling editor. I would therefore ask you to include your synopsis blurb and 3-5 bullet points listing the key experimental findings.
• Our production/data editors have asked you to clarify several points in the figure legends (see attached document). Please incorporate these changes in the attached word document and return it with track changes activated.

Thank you again for giving us to consider your manuscript for EMBO Reports, I look forward to your minor revision.

Kind regards,

Deniz Senyilmaz Tiebe

--
Deniz Senyilmaz Tiebe, PhD
Editor
EMBO Reports

Referee #1:

Some of my original concerns were addressed by the authors, but not all.

1) The experiment depicted in Figure 2C was not repeated and replaced. As I said before, this is a key figure, and the quality of the data is extremely poor. There is a dark smudge over the part of the blot that they highlight as a SDMA signal on vimentin. Again, this experiment needs to be repeated and the quality of the blot improved.

2) Also, point #3 was not fully addressed. I asked the authors to remove all indication that F is a mimic for Rme2s. They have still included this misleading term on page11, line17. This must be removed.

The SYM10 antibody validation using vimentin peptides was nicely performed.

Comments from the Referee #1:

C1. The experiment depicted in Figure 2C was not repeated and replaced. As I said before, this is a key figure, and the quality of the data is extremely poor. There is a dark smudge over the part of the blot that they highlight as a SDMA signal on vimentin. Again, this experiment needs to be repeated and the quality of the blot improved.

R1: We would like to thank the reviewer for the suggestion. In accordance with the suggestion, we have repeated the *in vitro* methyltransferase assays and replaced Figure 2C with a higher quality blot as shown below.

Revised Figure 2C

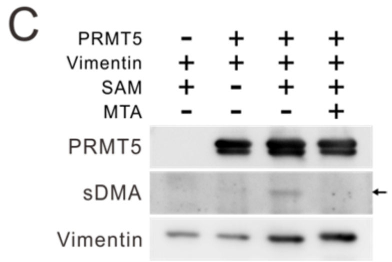

C. *In vitro* methyltransferase assays of vimentin by PRMT5 analyzed by Western blots with anti-sDMA antibody. Arrow marks dimethyl-vimentin.

C2. Also, point #3 was not fully addressed. I asked the authors to remove all indication that F is a mimic for Rme2s. They have still included this misleading term on page11, line17. This must be removed.

R2: We thank the reviewer for the comment. We have accordingly revised the description for the 4RF mutant in the Results section (page 11) and marked in blue.

Page 11, line 15 to line 18

To investigate if the functionality of vimentin is mediated by its sDMA level, endogenous vimentin in H1650 and CL1-5 cells was knocked out by CRISPR/Cas9 approaches and the effect of wild-type, 4RK and 4RF (R196/207/345/364F, bulky hydrophobic form) mutant vimentin on cell motility was evaluated.

2nd Revision - Editorial Decision 8th Jun 2022

Dear Sung-Liang,

Thank you for submitting your revised manuscript. I have now looked at everything and all is fine. Therefore, I am very pleased to accept your manuscript for publication in EMBO Reports.

Congratulations on a nice work!

Of note, I would like to remind you once again to make the dataset PXD031192 publicly available as soon as possible, which is prerequisite for publication. Thank you.

Kind regards,

Deniz
--
Deniz Senyilmaz Tiebe, PhD
Editor
EMBO Reports

--
Please note that you will be contacted by Wiley Author Services to complete licensing and payment information. The required 'Page Charges Authorization Form' is available here: https://www.embopress.org/pb-assets/embo-site/er_apc.pdf

--
At the end of this email I include important information about how to proceed. Please ensure that you take the time to read the information and complete and return the necessary forms to allow us to publish your manuscript as quickly as possible.

As part of the EMBO publication's Transparent Editorial Process, EMBO reports publishes online a Review Process File to accompany accepted manuscripts. As you are aware, this File will be published in conjunction with your paper and will include the referee reports, your point-by-point response and all pertinent correspondence relating to the manuscript.

If you do NOT want this File to be published, please inform the editorial office within 2 days, if you have not done so already, otherwise the File will be published by default [contact: emboreports@embo.org]. If you do opt out, the Review Process File link will point to the following statement: "No Review Process File is available with this article, as the authors have chosen not to make the review process public in this case."

Thank you again for your contribution to EMBO reports and congratulations on a successful publication. Please consider us again in the future for your most exciting work.

****

THINGS TO DO NOW:

Please note that you will be contacted by Wiley Author Services to complete licensing and payment information. The required 'Page Charges Authorization Form' is available here: https://www.embopress.org/pb-assets/embo-site/er_apc.pdf

You will receive proofs by e-mail approximately 2-3 weeks after all relevant files have been sent to our Production Office; you should return your corrections within 2 days of receiving the proofs.

Please inform us if there is likely to be any difficulty in reaching you at the above address at that time. Failure to meet our deadlines may result in a delay of publication, or publication without your corrections.

All further communications concerning your paper should quote reference number EMBOR-2021-54265V3 and be addressed to emboreports@wiley.com.

Should you be planning a Press Release on your article, please get in contact with emboreports@wiley.com as early as possible, in order to coordinate publication and release dates.

EMBO Press Author Checklist

Corresponding Author Name: Sung-Liang Yu	
Journal Submitted to: EMBO Reports	
Manuscript Number: EMBOR-2021-54265	

USEFUL LINKS FOR COMPLETING THIS FORM
The EMBO Journal - Author Guidelines
EMBO Reports - Author Guidelines
Molecular Systems Biology - Author Guidelines
EMBO Molecular Medicine - Author Guidelines

Reporting Checklist for Life Science Articles (updated January 2022)
This checklist is adapted from Materials Design Analysis Reporting (MDAR) Checklist for Authors. MDAR establishes a minimum set of requirements in transparent reporting in the life sciences (see Statement of Task: 10.31222/osf.io/9sm4x). Please follow the journal's guidelines in preparing your manuscript.
Please note that a copy of this checklist will be published alongside your article.

Abridged guidelines for figures
1. Data
The data shown in figures should satisfy the following conditions:
- the data were obtained and processed according to the field's best practice and are presented to reflect the results of the experiments in an accurate and unbiased manner.
- ideally, figure panels should include only measurements that are directly comparable to each other and obtained with the same assay.
- plots include clearly labeled error bars for independent experiments and sample sizes. Unless justified, error bars should not be shown for technical replicates.
- if n<5, the individual data points from each experiment should be plotted. Any statistical test employed should be justified.
- Source Data should be included to report the data underlying figures according to the guidelines set out in the authorship guidelines on Data Presentation.

2. Captions
Each figure caption should contain the following information, for each panel where they are relevant:
- a specification of the experimental system investigated (eg cell line, species name).
- the assay(s) and method(s) used to carry out the reported observations and measurements.
- an explicit mention of the biological and chemical entity(ies) that are being measured.
- an explicit mention of the biological and chemical entity(ies) that are altered/varied/perturbed in a controlled manner.
- the exact sample size (n) for each experimental group/condition, given as a number, not a range;
- a description of the sample collection allowing the reader to understand whether the samples represent technical or biological replicates (including how many animals, litters, cultures, etc.).
- a statement of how many times the experiment shown was independently replicated in the laboratory.
- definitions of statistical methods and measures:
  - common tests, such as t-test (please specify whether paired vs. unpaired), simple χ2 tests, Wilcoxon and Mann-Whitney tests, can be unambiguously identified by name only, but more complex techniques should be described in the methods section;
  - are tests one-sided or two-sided?
  - are there adjustments for multiple comparisons?
  - exact statistical test results, e.g., P values = x but not P values < x;
  - definition of 'center values' as median or average;
  - definition of error bars as s.d. or s.e.m.

Please complete ALL of the questions below.
Select "Not Applicable" only when the requested information is not relevant for your study.

Materials

Newly Created Materials	Information included in the manuscript?	In which section is the information available? (Reagents and Tools Table, Materials and Methods, Figures, Data Availability Section)
New materials and reagents need to be available; do any restrictions apply?	Yes	MATERIALS AND METHODS

Antibodies	Information included in the manuscript?	In which section is the information available? (Reagents and Tools Table, Materials and Methods, Figures, Data Availability Section)
For **antibodies** provide the following information:<br>- Commercial antibodies: RRID (if possible) or supplier name, catalogue number and or/clone number<br>- Non-commercial: RRID or citation	Yes	MATERIALS AND METHODS

DNA and RNA sequences	Information included in the manuscript?	In which section is the information available? (Reagents and Tools Table, Materials and Methods, Figures, Data Availability Section)
**Short novel DNA or RNA including primers, probes**: provide the sequences.	Yes	MATERIALS AND METHODS

Cell materials	Information included in the manuscript?	In which section is the information available? (Reagents and Tools Table, Materials and Methods, Figures, Data Availability Section)
**Cell lines:** Provide species information, strain. Provide accession number in repository **OR** supplier name, catalog number, clone number, and/**OR** RRID.	Yes	MATERIALS AND METHODS
**Primary cultures:** Provide species, strain, sex of origin, genetic modification status.	Not Applicable	
Report if the cell lines were recently **authenticated** (e.g., by STR profiling) and tested for mycoplasma contamination.	Yes	MATERIALS AND METHODS

Experimental animals	Information included in the manuscript?	In which section is the information available? (Reagents and Tools Table, Materials and Methods, Figures, Data Availability Section)
**Laboratory animals or Model organisms:** Provide species, strain, sex, age, genetic modification status. Provide accession number in repository **OR** supplier name, catalog number, clone number, **OR** RRID.	Yes	MATERIALS AND METHODS
**Animal observed in or captured from the field:** Provide species, sex, and age where possible.	Not Applicable	
Please detail **housing and husbandry conditions**.	Yes	MATERIALS AND METHODS

Plants and microbes	Information included in the manuscript?	In which section is the information available? (Reagents and Tools Table, Materials and Methods, Figures, Data Availability Section)
**Plants:** provide species and strain, ecotype and cultivar where relevant, unique accession number if available, and source (including location for collected wild specimens).	Not Applicable	
**Microbes:** provide species and strain, unique accession number if available, and source.	Not Applicable	

Human research participants	Information included in the manuscript?	In which section is the information available? (Reagents and Tools Table, Materials and Methods, Figures, Data Availability Section)
If collected and within the bounds of privacy constraints report on age, sex and gender or ethnicity for all study participants.	Yes	Appendix Table

Core facilities	Information included in the manuscript?	In which section is the information available? (Reagents and Tools Table, Materials and Methods, Figures, Data Availability Section)
If your work benefited from core facilities, was their service mentioned in the acknowledgments section?	Yes	ACKNOWLEDGEMENTS

Design

Study protocol	Information included in the manuscript?	In which section is the information available? (Reagents and Tools Table, Materials and Methods, Figures, Data Availability Section)
If study protocol has been **pre-registered, provide DOI in the manuscript**. For clinical trials, provide the trial registration number **OR** cite DOI.	Not Applicable	
Report the **clinical trial registration number** (at ClinicalTrials.gov or equivalent), where applicable.	Not Applicable	

Laboratory protocol	Information included in the manuscript?	In which section is the information available? (Reagents and Tools Table, Materials and Methods, Figures, Data Availability Section)
Provide DOI OR other citation details if **external detailed step-by-step protocols** are available.	Not Applicable	

Experimental study design and statistics	Information included in the manuscript?	In which section is the information available? (Reagents and Tools Table, Materials and Methods, Figures, Data Availability Section)
Include a statement about **sample size** estimate even if no statistical methods were used.	Not Applicable	
Were any steps taken to minimize the effects of subjective bias when allocating animals/samples to treatment (e.g. **randomization procedure**)? If yes, have they been described?	Not Applicable	
Include a statement about **blinding** even if no blinding was done.	Not Applicable	
Describe **inclusion/exclusion criteria** if samples or animals were excluded from the analysis. Were the criteria pre-established?<br><br>If sample or data points were omitted from analysis, report if this was due to attrition or intentional exclusion and provide justification.	Not Applicable	
For every figure, are **statistical tests** justified as appropriate? Do the data meet the assumptions of the tests (e.g., normal distribution)? Describe any methods used to assess it. Is there an estimate of variation within each group of data? Is the variance similar between the groups that are being statistically compared?	Not Applicable	

Sample definition and in-laboratory replication	Information included in the manuscript?	In which section is the information available? (Reagents and Tools Table, Materials and Methods, Figures, Data Availability Section)
In the figure legends: state number of times the experiment was **replicated** in laboratory.	Yes	FIGURE LEGENDS
In the figure legends: define whether data describe **technical or biological replicates**.	Yes	FIGURE LEGENDS

Ethics

Ethics	Information included in the manuscript?	In which section is the information available? (Reagents and Tools Table, Materials and Methods, Figures, Data Availability Section)
Studies involving **human participants**: State details of **authority granting ethics approval** (IRB or equivalent committee(s), provide reference number for approval.	Yes	MATERIALS AND METHODS
Studies involving **human participants**: Include a statement confirming that **informed consent** was obtained from all subjects and that the experiments conformed to the principles set out in the WMA Declaration of Helsinki and the Department of Health and Human Services Belmont Report.	Yes	MATERIALS AND METHODS
Studies involving **human participants:** For publication of **patient photos**, include a statement confirming that consent to publish was obtained.	Not Applicable	
Studies involving experimental **animals**: State details of **authority granting ethics approval** (IRB or equivalent committee(s), provide reference number for approval. Include a statement of compliance with ethical regulations.	Yes	MATERIALS AND METHODS
Studies involving **specimen and field samples:** State if relevant **permits** obtained, provide details of authority approving study; if none were required, explain why.	Not Applicable	

Dual Use Research of Concern (DURC)	Information included in the manuscript?	In which section is the information available? (Reagents and Tools Table, Materials and Methods, Figures, Data Availability Section)
Could your study fall under dual use research restrictions? Please check biosecurity documents and list of **select agents and toxins** (CDC): https://www.selectagents.gov/sat/list.htm	Not Applicable	
If you used a select agent, is the security level of the lab appropriate and reported in the manuscript?	Not Applicable	
If a study is subject to dual use research of concern regulations, is the name of the **authority granting approval and reference number** for the regulatory approval provided in the manuscript?	Not Applicable	

Reporting

The MDAR framework recommends adoption of discipline-specific guidelines, established and endorsed through community initiatives. Journals have their own policy about requiring specific guidelines and recommendations to complement MDAR.

Adherence to community standards	Information included in the manuscript?	In which section is the information available? (Reagents and Tools Table, Materials and Methods, Figures, Data Availability Section)
State if relevant guidelines or checklists (e.g., **ICMJE, MIBBI, ARRIVE, PRISMA**) have been followed or provided.	Not Applicable	
For **tumor marker prognostic studies,** we recommend that you follow the **REMARK** reporting guidelines (see link list at top right). See author guidelines, under 'Reporting Guidelines'. Please confirm you have followed these guidelines.	Not Applicable	
For **phase II and III randomized controlled trials**, please refer to the **CONSORT** flow diagram (see link list at top right) and submit the CONSORT checklist (see link list at top right) with your submission. See author guidelines, under 'Reporting Guidelines'. Please confirm you have submitted this list.	Not Applicable	

Data Availability

Data availability	Information included in the manuscript?	In which section is the information available? (Reagents and Tools Table, Materials and Methods, Figures, Data Availability Section)
Have **primary datasets** been deposited according to the journal's guidelines (see 'Data Deposition' section) and the respective accession numbers provided in the Data Availability Section?	Yes	DATA AVAILABILITY
Were **human clinical and genomic datasets** deposited in a public access-controlled repository in accordance to ethical obligations to the patients and to the applicable consent agreement?	Not Applicable	
Are **computational models** that are central and integral to a study available without restrictions in a machine-readable form? Were the relevant accession numbers or links provided?	Not Applicable	
If publicly available data were reused, provide the respective **data citations in the reference list**.	Not Applicable	
